# Artificial Intelligence in Gastrointestinal Surgery: A Systematic Review of Its Role in Laparoscopic and Robotic Surgery

**DOI:** 10.3390/jpm15110562

**Published:** 2025-11-19

**Authors:** Ludovica Gorini, Roberto de la Plaza Llamas, Daniel Alejandro Díaz Candelas, Rodrigo Arellano González, Wenzhong Sun, Jaime García Friginal, María Fra López, Ignacio Antonio Gemio del Rey

**Affiliations:** 1 Department of General and Digestive Surgery, Hospital Universitario de Guadalajara, Calle del Donante de Sangre s/n, 19002 Guadalajara, Spain; lgorini@sescam.jccm.es (L.G.); ddcandelas@sescam.jccm.es (D.A.D.C.); rarellanogonzalez@sescam.jccm.es (R.A.G.); wsun@sescam.jccm.es (W.S.); jgfriginal@gmail.com (J.G.F.); mfra@sescam.jccm.es (M.F.L.); ignacio.gemio@uah.es (I.A.G.d.R.); 2 Department of Surgery, Medical and Social Sciences, Faculty of Medicine and Health Sciences, University of Alcalá, Alcalá de Henares, 28871 Madrid, Spain

**Keywords:** artificial intelligence, laparoscopic surgery, robotic surgery, general surgery, deep learning, surgical training, intraoperative guidance

## Abstract

**Background**: Artificial intelligence (AI) is transforming surgical practice by enhancing training, intraoperative guidance, decision-making, and postoperative assessment. However, its specific role in laparoscopic and robotic general surgery remains to be clearly defined. The objective is to systematically review the current applications of AI in laparoscopic and robotic general surgery and categorize them by function and surgical context. **Methods**: A systematic search of PubMed and Web of Science was conducted up to 22 June 2025, using predefined search terms. Eligible studies focused on AI applications in laparoscopic or robotic general surgery, excluding urological, gynecological, and obstetric fields. Original articles in English or Spanish were included. Data extraction was performed independently by two reviewers and synthesized descriptively by thematic categories. **Results**: A total of 152 original studies were included. Most were conducted in laparoscopic settings (*n* = 125), while 19 focused on robotic surgery and 8 involved both. The majority were technical evaluations or retrospective observational studies. Seven thematic categories were identified: surgical decision support and outcome prediction; skill assessment and training; workflow recognition and intraoperative guidance; object or structure detection; augmented reality and navigation; image enhancement; technical assistance; and surgeon perception and preparedness. Most studies applied deep learning, for classification, prediction, recognition, and real-time guidance in laparoscopic cholecystectomies, colorectal and gastric surgeries. **Conclusions**: AI has been widely adopted in various domains of laparoscopic and robotic general surgery. While most studies remain in early developmental stages, the evidence suggests increasing maturity and integration into clinical workflows. Standardization of evaluation and reporting frameworks will be essential to translate these innovations into widespread practice.

## 1. Introduction

Artificial intelligence (AI) is the center of modern technological innovation. Rapidly growing in many fields, it opens us up to opportunities that were unthinkable a decade ago, giving space to creativity and engineering innovation as it expands. Since 1956, when the term was coined, this new technological revolution has not stopped growing, making its way into medicine [1].

General Surgery is a fertile land for AI to grow into a powerful tool for surgeons, and the introduction of AI has had a crucial role, especially in laparoscopic and robotic procedures, where synergy between surgeons and machines has already gone far, implementing technological innovation into surgical tradition.

The role of AI in surgery has many facets, including pattern recognition in standard procedures to improve safety, tissue or tool identification [2], anatomical structure mapping, integration of radiologic imaging during surgery, the use of predictive models to guide instrumentation, surgical training and skill assessment [3,4]. Additionally, it is utilized for surgical decision support, optimization, and outcome prediction [5,6].

AI in surgery is evolving beyond isolated image task-based applications, integrating data from other fields such as radiology or pathology, aiming to a personalized and integrated intraoperative decision-making [7].

The future seems to point toward the integration of AI throughout the patient journey, from preoperative screening, intraoperative assistance, and postoperative monitoring to follow-up [8].

The integration of AI in surgical robotics is paving the way for autonomous systems, with increased precision and more consistent surgical outcomes, reducing reliance on human factors such as fatigue and variability [9].

As has happened in the past when a new element was added to the clinical practice, ethical considerations have been raised, and AI is no exception. These concerns include privacy, potential bias in the systems and algorithms, autonomy and explainability in the decision-making processes, as well as legal responsibility and institutional readiness [9,10,11].

Although previous reviews have examined AI in robotic surgery or focused narrowly on technical skills assessment, few have captured the full clinical spectrum of AI integration in both laparoscopic and robotic general surgery [2,6,12,13].

In this context, the present review provides an integrated overview of how AI is being applied across these two minimally invasive modalities, summarizing diverse technical and clinical perspectives. Unlike previous reviews that have addressed specific subspecialties or technical domains, it brings together evidence from diverse areas to outline current trends, practical applications, and methodological gaps. By doing so, this study contributes to a broader understanding of the current state and potential directions for the clinical translation of AI within minimally invasive surgery.

The primary aim of this review was to address this gap, synthesizing current applications of AI in laparoscopic and robotic general surgery. Secondary objectives were to assess methodological trends and gaps in validation and to actualize the available knowledge in a rapidly growing field.

## 2. Materials and Methods

A systematic review was conducted following the PRISMA 2020 Guidelines [14].

The search was performed on PubMed and Web of Science databases using the following Boolean string: (“artificial intelligence” OR AI) AND (surgery) AND (“laparoscopic surgery” OR laparoscopy OR “robotic surgery”) NOT (urology OR gynecology OR gynaecology OR urological OR obstetrics). No date restrictions were applied. The last search was conducted on 22 June 2025. Only articles published in English or Spanish and related to the field of surgery were considered.

The research question followed a modified PICO framework, defining the Population as studies in laparoscopic and robotic general surgery, the Intervention as AI-based applications, and the Outcomes as thematic objectives and validation approaches. A formal Comparator was not applicable due to the descriptive scope of the review.

Eligibility criteria included:

- Articles focusing on laparoscopic or robotic procedures performed in General Surgery settings (Under the term “General Surgery”, the following fields were included: abdominal surgery, including colorectal, upper gastrointestinal, bariatric, endocrine, abdominal wall, oncologic, hepatopancreaticobiliary, minimally invasive surgeries and transplant; trauma and breast surgeries).
- AI as the focus of the research, applied in a clinical context.
- Articles presenting original data.

The exclusion criteria applied were:

- Studies focused on specialties outside General Surgery (e.g., Urology or Gynecology).
- AI not applied in a clinical setting (e.g., theoretical models or models without a specific surgical application).
- Reviews, meta-analyses, abstracts.
- Non-human studies (studies were included if an animal model was employed, for instance, in a training setting, but the final objective was human surgery).

Two reviewers completed the screening process, followed by data extraction, first performing title and abstract screening and second a full text review. If consensus was not reached, a third author was consulted.

We extracted key data from each article including year of publication, country of origin, type of surgery (laparoscopic, robotic, or both), study design, AI methodology, type of AI application (e.g., classification, prediction, segmentation), surgical procedure, and thematic category.

Given the methodological heterogeneity and descriptive scope of this review, a formal risk of bias assessment was not conducted. The aim was to provide a broad overview of current applications rather than to evaluate comparative effectiveness.

A meta-analysis was not possible due to the variability in study designs, outcomes, and AI applications. Results were synthesized narratively by thematic category, and heterogeneity was described qualitatively rather than statistically.

Institutional Review Board approval was not required, as this study analysed only previously published data and did not involve human subjects or confidential patient information.

### Studies Classification

According to their type, studies were classified under the following categories:

- Technical evaluations (evaluation of new AI-based tools or algorithms, often with proof-of-concept validation).
- Retrospective observational studies.
- Prospective observational studies.
- Feasibility studies (preliminary testing of AI applications in real or simulated surgical settings).
- Clinical trials.
- Dataset descriptions (description of publicly available or novel surgical datasets annotated for AI development and benchmarking).
- Simulation-based training assessments.
- Surveys or expert opinion studies.

Articles were also classified into seven thematic categories according to their primary focus:

- Surgical decision support and outcome prediction: studies that used AI to assist in preoperative or intraoperative clinical decision-making, risk stratification, or prediction of postoperative outcomes.
- Skill assessment and training: research focused on evaluating or improving surgical performance, through AI-based performance metrics, simulation platforms, or feedback systems.
- Workflow recognition and intraoperative guidance: articles addressing the use of AI to identify surgical phases, provide context-aware support, or optimize procedural flow during surgery.
- Object or structure detection: studies aimed at recognizing anatomical structures, surgical instruments, or landmarks within the operative field.
- Augmented reality (AR) and navigation: research involving the integration of preoperative imaging and real-time intraoperative views.
- Image enhancement: studies focused on improving the visual quality of surgical imaging.
- Surgeon perception, preparedness, and attitudes: Studies exploring surgeons’ knowledge, acceptance, and perceived challenges regarding the integration of AI and digital technologies into surgical practice.

The methodological quality of the included studies was evaluated using validated, design-specific tools: PROBAST for predictive and prognostic models, ROBINS-I for non-randomized comparative studies, QUADAS-AI for intraoperative computer-vision and guidance systems, MINORS for experimental or feasibility studies, and AXIS for survey-based research.

Each study was independently appraised across the main bias domains and then harmonized into a three-level classification (low, moderate, or high risk of bias) to enable cross-domain synthesis.

## 3. Results

**Figure 1:**
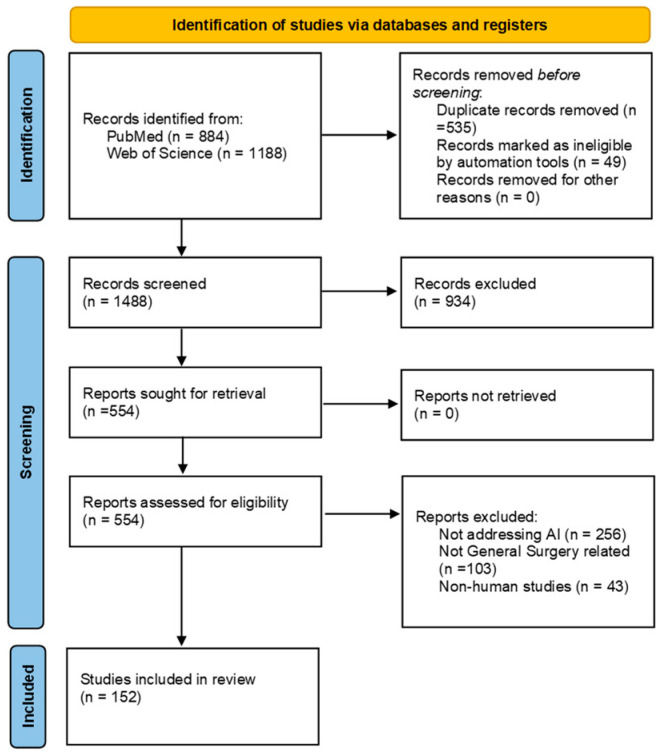
PRISMA diagram detailing the review process.

A total of 152 original studies were included in this review. The selection process is detailed in the PRISMA diagram shown in Figure 1.

The number of publications has increased over time, with 49 articles published in 2024, 34 in 2023, and 28 in 2022. Most studies were conducted in laparoscopic surgery settings (*n* = 125), while 19 focused on robotic surgery and 8 involved both approaches.

Most studies were classified as technical evaluations (*n* = 65) or retrospective observational studies (*n* = 35). Feasibility studies (*n* = 14) and prospective observational studies (*n* = 13) were reported in similar numbers. Clinical trials, dataset descriptions, simulation-based training assessments, and survey-based studies were less common, each represented by fewer than ten publications.

Most studies employed deep learning-based approaches, particularly convolutional neural networks (CNNs). AI methods were applied for classification, prediction, real-time guidance, and anatomical recognition. Specifically, 31 studies focused on recognition, 22 involved real-time AI applications, 20 addressed prediction, and 18 focused on classification. Detection tasks and segmentation were less frequently studied, appearing in 16 and 4 studies, respectively.

Regarding the clinical focus, the most frequently studied procedures or surgical fields were laparoscopic cholecystectomy (*n* = 27), gastrectomy (*n* = 12), and rectal surgery (*n* = 12), followed by other procedures in colorectal surgery (*n* = 14) and hernia repair (*n* = 5).

**Figure 2:**
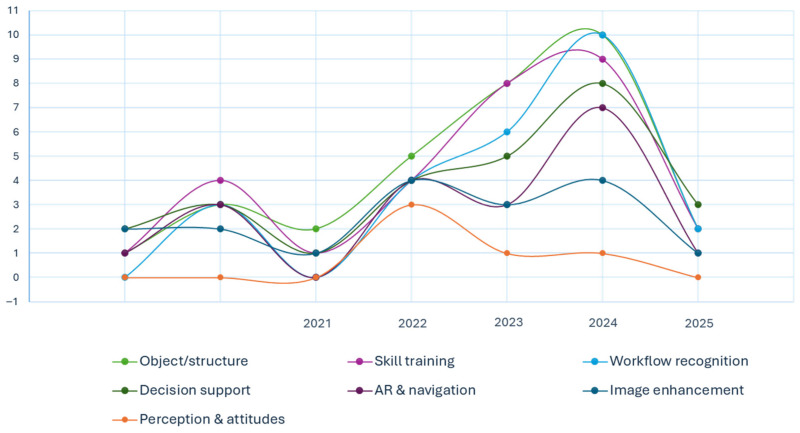
Time-series distribution of included studies by category from 2015 to 2025.

Descriptive data are shown in Table 1 and Figure 2.

Articles were categorized into seven thematic groups based on their primary focus. The number of studies and a brief description of each category are detailed below.

### 3.1. AI for Object or Structure Detection

A total of 51 studies focused on the use of artificial intelligence for the identification and localization of anatomical structures or surgical tools within the operative field. This category included 37 technical evaluations, 7 feasibility studies, 6 prospective observational studies and one dataset description. Most studies were conducted in laparoscopic surgery (*n* = 45, 84%), while only a small minority focused exclusively on robotic approaches (*n* = 2). Four studies included both laparoscopic and robotic data, mainly in colorectal and liver resections.

The most frequent applications were related to laparoscopic cholecystectomy (*n* = 17), particularly for the automatic identification of the critical view of safety, and recognition of biliary or vascular anatomy. Colorectal surgery (*n* = 8) and gastrectomy (*n* = 2) focused on vascular or autonomic nerve identification to reduce intraoperative injury. More recent studies (2023–2025) have increasingly explored bleeding detection, nerve recognition, and real-time safety checks in laparoscopic colectomy and rectal cancer surgery, reflecting a shift toward intraoperative decision support beyond static anatomical segmentation.

The applications primarily leveraged on computer vision techniques (CNNs), to detect and segment organs, vessels, anatomical landmarks, and instruments in laparoscopic or robotic video frames. Several studies developed real-time models capable of highlighting key structures such as anatomical elements or nerves during surgery to prevent complications, with reported accuracies between 80–90%. Other investigations aimed to improve instrument tracking, recognize energy device activation, or monitor tool presence outside the camera view, achieving detection accuracies of 85–95% in most reports. These AI-based detection tools aimed to enhance intraoperative safety, support training, and enable automated annotation of surgical videos for post-operative analysis.

Importantly, only 6 studies evaluated algorithms in intraoperative conditions, while the majority relied on retrospective datasets or simulation environments. External validation was reported in 3 studies, underscoring the limited generalizability of current models.

Several studies have demonstrated AI’s utility in real-time anatomical recognition and intraoperative guidance, such as automatic identification of the critical view of safety during laparoscopic cholecystectomy [15], detection of peritoneal surface metastases in staging laparoscopy [16], and recognition of perigastric vessels [17], intrahepatic vascular structures [18] or autonomic nerves during colorectal surgery [19] to reduce intraoperative injury.

### 3.2. AI for Surgical Skill Assessment and Training

A total of 37 studies were included in this category, focusing on the development and application of AI tools to evaluate technical performance and facilitate surgical training. These studies predominantly utilized video recordings of simulated or real procedures to assess skills such as suturing, dissection, and instrument handling, aiming to automate performance scoring based on standardized metrics and provide personalized feedback for trainees. CNNs and other deep learning frameworks were frequently applied to extract features from motion trajectories, tool usage, or procedural steps.

Twenty-one studies were technical evaluations, 6 were observational studies, 7 were simulation-based training assessments, and the rest included feasibility studies (*n* = 1), clinical trials (*n* = 1), and dataset descriptions (*n* = 1). Most studies were performed in laparoscopic settings (*n* = 26, 70%), while robotic applications accounted for a smaller but substantial proportion (*n* = 11, 30%), primarily focusing on suturing skills, task classification, and stress prediction.

Most of these relied on simulators or retrospective video data, while only about five studies (15%) assessed intraoperative material in real surgical procedures, such as laparoscopic gastrectomy, sigmoidectomy, or hernia repair. External or multicenter validation was rare, reported in only 3 studies.

In this context, Wu et al. [20] proposed video-based AI systems to objectively assess surgical performance, during laparoscopic cholecystectomy. Other studies like Halperin et al. [21] and Chen et al. [22] proposed real-time feedback on intracorporeal suture for laparoscopic and robotic training.

Overall, reported accuracies for automated skill classification ranged from 75–90%, with some systems distinguishing between novice, intermediate, and expert performance levels. More recent studies increasingly focused on real-time feedback tools and multicenter datasets, suggesting a gradual move toward clinically applicable systems.

### 3.3. AI for Workflow Recognition and Intraoperative Guidance

This category included 28 studies that explored the use of AI to recognize surgical workflows, identify procedural phases, and provide intraoperative assistance.

Twenty-two studies were technical evaluations, three were dataset descriptions and one was a feasibility study.

These studies relied on video-based inputs from laparoscopic or robotic procedures, annotated for phases, actions, or tool usage. Deep learning models (CNNs) were trained to recognize distinct steps of procedures such as cholecystectomy, gastrectomy, and colorectal resections as well as to estimate procedure duration or detect deviations from standard workflows.

Most studies (*n* = 23, 82%) were conducted in laparoscopic settings, while only three focused exclusively on robotic surgery (*n* = 11%) and one included both approaches. Six studies (21%) tested their models intraoperatively, while the majority remained retrospective or simulation based. External or multicenter validation was reported in four studies, often using large cholecystectomy datasets.

The procedures most frequently studied were laparoscopic cholecystectomy (*n* = 9), followed by other hepatopancreatobiliary procedures (*n* = 7), gastric surgery (*n* = 6), colorectal surgery (*n* = 2), and hernia repair (*n* = 1). The remaining studies focused on general laparoscopic tasks without procedure-specific targeting.

A subset of studies focused on real-time phase recognition to support context-aware surgical systems. All studies aimed to enable advanced decision support and integration into autonomous or semi-autonomous surgical platforms.

Some examples of AI models applied to surgical workflow recognition are related to enabling automatic phase identification, operative step classification and predict complexity in procedures such as laparoscopic sleeve gastrectomy [23] or robotic distal gastrectomy [24], supporting intraoperative guidance and process optimization.

### 3.4. AI for Surgical Decision Support and Outcome Prediction

This category encompassed 14 studies focused on leveraging AI to support preoperative or intraoperative decision-making and to predict clinical outcomes. The most targeted outcomes included postoperative complications, surgical complexity, and length of hospital stay. Most of these studies (*n* = 11) were retrospective and centered on model development using clinical data, imaging, or a combination of both. The rest of the studies included an algorithm development study, a feasibility study, and a comparative model analysis. Only two studies incorporated intraoperative evaluations, while three reported multicenter or multi-institutional validation, mainly in liver and gastric surgery. Deep learning methods, particularly CNNs, were employed to develop prediction algorithms. The models used integrated multimodal data sources, such as operative videos and radiological imaging, to enhance predictive accuracy. Reported predictive performance varied, with sensitivity and specificity ranging from 70% to 90% when predicting complications, conversion, or anastomotic risk.

These tools were primarily designed to assist in stratifying risks and optimizing surgical planning or intraoperative decisions. Most studies (*n* = 11, 79%) were laparoscopic, while one was robotic, and two included both approaches. Colorectal surgery was the most represented subspecialty (*n* = 7), followed by gastric (*n* = 2), liver (*n* = 2), and fewer isolated studies in cholecystectomy and appendectomy.

Notable examples include models that predict surgical complexity and postoperative outcomes in laparoscopic liver surgery [25] and tools to assess the risk of postoperative complications in patients undergoing laparoscopic radical gastrectomy [26].

### 3.5. AI for Augmented Reality and Navigation

A total of 11 studies explored the integration of artificial intelligence with AR systems or intraoperative navigation tools. Six studies were technical evaluations, four were feasibility studies and one a dataset description. All studies in this category were performed in laparoscopic settings.

These studies primarily addressed the fusion of preoperative imaging, such as computed tomography (CT) or magnetic resonance imaging (MRI), with real-time laparoscopic video to enhance anatomical orientation during surgery. AI methods were used to automate landmark detection, register 3D anatomical models of the intraoperative view, and improve surgeon perception of depth and spatial relationships. Four studies included intraoperative feasibility testing, but none performed external or multicenter validation. Several investigations also proposed AR-based systems to support surgical planning or intraoperative decision-making, particularly in liver, pancreatic, and colorectal surgery. Liver resection and portal mapping (*n* = 2) and colorectal applications (*n* = 2) were the most frequent, followed by single studies in gastrectomy and other procedures. These tools aimed to assist with tumor localization, vascular mapping, or dissection plane guidance, improving precision and potentially reducing intraoperative risks.

Recent developments include the integration of AI-enhanced AR systems for intraoperative navigation, such as real-time anatomical overlay during laparoscopic liver resection [27] and the identification of anatomical landmarks associated with postoperative pancreatic fistula during laparoscopic gastrectomy [28].

### 3.6. AI for Image Enhancement

A total of 8 studies focused on improving the visual quality of laparoscopic or robotic video through artificial intelligence-based image enhancement techniques. Four technical evaluation studies were included, along with 3 feasibility studies and 1 prospective observational study. These approaches aimed to address common intraoperative visibility challenges such as surgical smoke, lens fogging, image blur, and poor lighting. AI models were developed to process and clarify real-time video feeds, remove visual artifacts, and enhance image quality. Most studies were conducted in laparoscopic settings (*n* = 8), while only one targeted robotic surgery. Three studies evaluated their systems in intraoperative conditions, but none included multicenter validation.

Some studies proposed context-aware systems capable of selectively displaying relevant structures or reducing visual overload by adapting output to the surgeon’s needs. Procedure-specific applications included vascular detection during laparoscopic cholecystectomy, perfusion assessment in colorectal surgery, and image enhancement during robotic gastric surgery. These enhancements aimed to improve intraoperative safety, efficiency, and diagnostic accuracy during minimally invasive procedures.

Some examples include real-time arteries detection using image-guided techniques during laparoscopic cholecystectomy [29] and a self-learning cognitive robotic camera system that improved camera guidance efficiency with continued use [30].

### 3.7. Surgeon Perception, Preparedness, and Attitudes

Three studies explored the perspectives, knowledge, and preparedness of surgeons regarding the integration of artificial intelligence in surgical practice. These works used survey-based methodologies to assess attitudes toward AI, perceived benefits and limitations, and the level of familiarity with digital surgery concepts. The themes investigated included the knowledge gap between surgeons actively engaged in robotic surgery and those with less exposure to technology, with the former group demonstrating higher awareness and interest [31]. Research was conducted regarding cognitive and psychomotor load during training or simulated tasks, using biosignals such as EEG or eye-tracking to understand mental workload [32]. This thematic area highlights the importance of addressing human factors in the adoption of AI tools and the need for structured educational interventions.

A summary of the articles included is shown in Table 2.

### 3.8. Risk of Bias Assessment

Methodological quality assessment showed that across the included studies, 31.4% were rated at low risk of bias, 58% at moderate, and 10.6% at high.

External validation was reported in 20% of studies, and a multicenter design in 27.1%.

Among thematic domains, Workflow Recognition and Intraoperative Guidance and Augmented Reality and Navigation achieved the highest methodological rigor, frequently incorporating multicentric datasets and external testing.

Conversely, Skill Assessment and Training and Image Enhancement presented the greatest variability and limited validation, reflecting their predominantly pre-clinical or simulation-based nature.

Overall, the quality appraisal indicated a maturing yet still heterogeneous evidence base for artificial intelligence in minimally invasive surgery.

These results are shown in Table 3.

### 3.9. Quantitative Synthesis by Thematic Domain

To enhance comparability across heterogeneous studies, standardized quantitative metrics were extracted and summarized by thematic category (Table 4). For each domain, representative median values, validation characteristics, and methodological features were compiled. Overall, reported performance metrics were consistently high, with median accuracies ranging between 0.84 and 0.90 for classification tasks and median AUC values of 0.86 for predictive models.

However, external validation remained limited (20–30% of studies) and standardized reporting of evaluation metrics was inconsistent, particularly in pre-clinical or simulation-based works.

Detailed study-level information, including the risk-of-bias assessment tool and overall judgment, as well as AI model characteristics, dataset properties, validation type, and key performance metrics for each included study, are provided in the Supplementary Materials (Supplementary Tables S1 and S2).

## 4. Discussion

AI is reshaping minimally invasive surgery by addressing key challenges in training, performance assessment, and intraoperative decision-making. The increasing number of studies highlights growing interest, though the prevalence of technical and retrospective studies reflects the field’s exploratory stage rather than mature clinical integration.

Despite encouraging technical and performance metrics, few studies have demonstrated a measurable impact on real clinical outcomes such as postoperative complication rates, conversion to open surgery, operative time, or length of stay. Most available reports remain pre-clinical, retrospective, or simulation-based, which limits external validity and generalizability to diverse surgical environments. Moreover, intraoperative AI systems are rarely integrated into decision-making processes in real time, meaning their potential to enhance safety or efficiency has not yet been fully verified in patient populations. Translational progress will require robust prospective and multicenter studies explicitly designed to evaluate not only algorithmic accuracy but also clinical endpoints and workflow integration. Only through such validation can AI move from proof-of-concept to demonstrable improvement in surgical outcomes and patient care.

Standardized training and validation protocols are essential for safe and effective integration, as emphasized in recent literature [11,163].

Most AI applications have been developed in laparoscopic surgery, while robotic procedures remain less explored. This gap reflects the wider global use and data availability of laparoscopy, suggesting that as robotic adoption expands, future work should address its specific integration with AI.

In surgical skill assessment and training, AI has shown potential to automate evaluation and improve objectivity. Several models successfully classify surgical expertise levels or offer real-time feedback on instrument movements [20,21,99]. However, most studies rely on simulators and retrospective video analysis, which constrains external validity and limits translation into routine practice [164].

Intraoperative workflow recognition and guidance studies focus on phase detection, action recognition, or remaining surgery duration. While some models demonstrate real-time application potential [23,24,156], substantial variability in annotation standards, surgical procedures, and evaluation metrics continues to limit comparability and widespread clinical adoption.

Artificial intelligence applied to object and structure recognition has yielded encouraging results, particularly in enhancing intraoperative safety through the identification of anatomical landmarks and surgical instruments. Recent systems can recognize critical anatomy during cholecystectomy or identify nerves and vessels in complex procedures [15,16,17,19,56,57,58,59,123]. Despite these advances, generalizability across institutions, patient populations, and imaging modalities remains limited, and external validation is needed [165].

AR and navigation tools have incorporated AI to align preoperative imaging with intraoperative video and improve spatial orientation. Studies demonstrate feasibility in laparoscopic liver and colorectal surgery [28,148], though robust, real-time accuracy under diverse intraoperative conditions has yet to be achieved.

Efforts to enhance image quality show improved clarity and performance using AI-based systems in robotic and laparoscopic settings [29,30]. Still, real-world adoption remains limited by challenges in hardware integration, workflow disruption, and regulatory approval pathways.

AI applications for decision support and outcome prediction remain limited but are growing with few models supporting intraoperative decisions such as resection margins or anastomotic risk, while others integrate perioperative data to predict complications or long-term outcomes [25,26]. Nevertheless, these remain largely retrospective modeling efforts, underscoring the need for prospective validation in heterogeneous patient cohorts.

Finally, studies exploring surgeons’ perceptions toward AI reveal varying levels of knowledge, with robotic-trained surgeons showing greater awareness. Surgeons generally support AI’s integration but stress the importance of explainability and training [163,166,167]. These findings emphasize that end-user perspectives must inform system design and implementation strategies to facilitate acceptance and adoption.

From a clinical perspective, AI systems show the greatest immediate utility in recognition of anatomical landmarks, skill assessment in training programs, and workflow optimization, especially in the most common procedures, such as cholecystectomies and colorectal and gastric surgeries. Areas requiring further supervision and refinement include outcome prediction models, AR-guided navigation, and autonomous camera control, where evidence remains preliminary.

Together, these findings suggest that while AI in surgery is advancing rapidly, its routine clinical adoption depends on standardization of reporting frameworks, prospective multicenter validation, seamless integration into surgical workflows, and responsiveness to surgeons’ practical needs.

The risk-of-bias assessment revealed a field that is technically advanced but methodologically uneven. Most studies demonstrated moderate risk, primarily due to limited dataset diversity, lack of prospective design, and incomplete external validation.

Encouragingly, the proportion of multicenter and prospectively validated works has grown in recent years, indicating a gradual shift toward clinically oriented and reproducible research. Nevertheless, progress toward clinical adoption will depend on standardization of performance metrics, transparent model reporting, and the design of multicenter prospective trials evaluating true patient-level outcomes.

These findings underscore that while AI in minimally invasive surgery is rapidly evolving, robust methodological frameworks remain essential to translate innovation into measurable improvements in surgical safety and effectiveness.

Beyond technical validation, the implementation of AI raises important ethical and legal considerations. These include issues of informed consent for AI-assisted decision-making, accountability in case of adverse outcomes, algorithmic bias, and equitable access. Establishing clear regulatory pathways and institutional policies will be essential to ensure safe and ethical adoption.

Although most studies remain in an early exploratory stage, the evidence summarized in this review already outlines what can be expected from the integration of artificial intelligence in surgical practice. The impact on intraoperative decision-making is particularly evident, as AI systems demonstrate the capacity to enhance safety through the recognition of critical anatomy, anticipation of adverse events, and real-time guidance during complex manoeuvres. Beyond the operating room, the studies analysed suggest that AI will likely become an integral part of the entire patient pathway, supporting preoperative risk assessment, individualized surgical planning, and postoperative monitoring. By linking these stages, AI has the potential to establish a continuous feedback cycle that informs surgical judgment, optimizes patient outcomes, and advances the overall quality and consistency of care.

This study has several limitations. The included studies exhibited considerable heterogeneity in design, outcome metrics, and validation strategies, which precluded quantitative synthesis. Most were single-center, retrospective, and lacked external or prospective validation. Laparoscopic procedures, particularly cholecystectomy, were also overrepresented compared with other surgical domains, which may limit generalizability. In addition, publication bias may have favoured technically successful or positive studies. Finally, the review was not pre-registered. Nevertheless, these limitations reflect the current state of the literature rather than methodological flaws of this review and underscore the need for future prospective, standardized, and multicenter investigations.

## 5. Conclusions

As AI continues to evolve, its successful implementation will depend not only on technological advancement but also on surgeon training and trust in these systems.

AI in robotic and laparoscopic surgery has moved beyond the experimental stage of prototypes and theoretical models; it is beginning to find practical clinical applications. Even if its use is still uneven, this review shows that current applications already coexist with numerous emerging opportunities for investigation. It also stresses the importance for surgeons to remain engaged with ongoing advances in AI, while critically addressing ethical, legal, and regulatory challenges that may arise.

## Figures and Tables

**Table 1:** Descriptive analysis of the reviewed articles.

Characteristic	Number of Studies (Total *n* = 152)
Year of Publication	
Articles published before 2015	7
Articles published in between 2015 and 2020	18
Articles published in 2021	5
Articles published in 2022	28
Articles published in 2023	34
Articles published in 2024	49
Articles published in 2025	11
Type of surgery	
Laparoscopic surgery	125
Robotic surgery	19
Laparoscopic and Robotic surgery	8
Study Type	
Technical evaluations	65
Retrospective observational studies:	35
Prospective observational studies	14
Feasibility studies	13
Clinical trials	8
Dataset descriptions	7
Simulation-based training assessments	6
Surveys or expert opinion studies	4
Study Categories	
Object or structure detection	51
Skill assessment and training	37
Workflow recognition and intraoperative guidance	28
Surgical decision support and outcome prediction	14
Augmented reality and navigation	11
Image enhancement	8
Surgeon perception, preparedness, and attitudes	3

**Table 2:** Included articles, summarized. TME: Total Mesorectal Excision; TaTME: Transanal Total Mesorectal Excision; CVS: Critical View of Safety; LLR: Laparoscopic Liver Resection; TAPP: Transabdominal Preperitoneal hernia repair; LC: Laparoscopic Cholecystectomy; AR: Augmented Reality; TEP: Totally Extraperitoneal; VR: Virtual Reality; RALIHR: Robotic-Assisted Laparoscopic Inguinal Hernia Repair; NIR: Near-Infrared; MIS: Minimally Invasive Surgery.

First Author and Year	DOI	Type of Surgery	Study Type	Study Objective
Object or Structure Detection				
Khalid 2023 [33]	10.1007/s00464-023-10403-4	Laparoscopic	Retrospective validation study	Prediction of safe and unsafe dissection zones during laparoscopic cholecystectomy.
Ward 2022 [34]	10.1007/s00464-022-09009-z	Laparoscopic	Retrospective model	To classify inflammation based on gallbladder images.
Orimoto 2025 [35]	10.1007/s00464-024-11514-2	Laparoscopic	Retrospective model	Identify intraoperative scarring in laparoscopic cholecystectomy for acute cholecystitis.
Kolbinger 2023 [36]	10.1097/JS9.0000000000000595	Laparoscopic	Algorithm development + comparison study	Segmentation of abdominal anatomy.
Sato 2022 [37]	10.1007/s00595-022-02508-5	Laparoscopic and Robotic	Feasibility study	Pancreas contouring for navigation in lymphadenectomy.
Igaki 2022 [38]	10.1097/DCR.0000000000002393	Laparoscopic	Single-center feasibility study	Segmentation-based image-guided navigation system for TME dissection.
Jearanai 2023 [39]	10.1007/s00464-023-10309-1	Laparoscopic	Technical development + validation	Detect abdominal wall layers during trocar insertion.
Oh 2024 [40]	10.1038/s41598-024-73434-4	Laparoscopic	Algorithm development + intraoperative support	Identify biliary structures.
Benavides 2024 [41]	10.3390/s2413419	Laparoscopic	Technical development	Localization of surgical tools in laparoscopic surgery.
Gazis 2022 [42]	10.3390/bioengineering9120737	Laparoscopic	Technical development	To recognize surgical gestures.
Tomioka 2023 [43]	10.21873/anticanres.16725	Laparoscopic	Technical development	Recognition of hepatic veins and Glissonean pedicle.
Cui 2021 [44]	10.1155/2021/5578089	Laparoscopic	Technical development	To detect vas deferens in laparoscopic inguinal hernia repair.
Memida 2023 [45]	10.1109/EMBC40787.2023.10341025	Laparoscopic	Technical development	To identify surgical instruments in laparoscopic procedures.
Wesierski 2018 [46]	10.1016/j.media.2018.03.012	Robotic	Technical development	To estimate the pose of multiple non-rigid and robotic surgical tools.
Jurosch 2024 [47]	10.1007/s11548-024-03220-0	Laparoscopic	Technical development	To detect trocars and assess their occupancy.
Sánchez-Brizuela 2022 [48]	10.3390/s22145180	Laparoscopic	Technical development	To identify surgical gauze in real time.
Lai 2023 [49]	10.1007/s10439-022-03033-9	Laparoscopic	Technical development	To detect surgical gauze in real time.
Ehrlich 2022 [50]	10.3390/s22155808	Robotic	Technical development	To detect energy events from electrosurgical tools.
Nwoye 2019 [51]	10.1007/s11548-019-01958-6	Laparoscopic	Methodological innovation	To enable real-time tool tracking.
Carstens 2023 [52]	10.1038/s41597-022-01719-2	Laparoscopic	Dataset publication	Semantic segmentations of abdominal organs and vessels.
Yin 2024 [53]	10.1016/j.neunet.2023.11.055	Laparoscopic	Technical development	Segmentation model for TaTME procedures.
Tashiro 2024 [54]	10.1002/jhbp.1422	Laparoscopic	Retrospective analysis	To recognize and color-code loose connective tissue.
Petracchi 2024 [15]	10.1016/j.gassur.2024.03.018	Laparoscopic	Prospective observational study	To detect the critical view of safety during elective LC.
Schnelldorfer 2024 [16]	10.1097/SLA.0000000000006294	Laparoscopic	Prototype development	Guidance system for identifying peritoneal metastases.
Kitaguchi 2023 [55]	10.1093/bjs/znad249	Laparoscopic	Prospective observational study	Organ recognition models.
Chen 2025 [17]	10.1093/bjsopen/zrae158	Laparoscopic	Retrospective study	Perigastric vessel recognition.
Han 2024 [56]	10.1097/DCR.0000000000003547	Laparoscopic	Model development	Neurorecognition during total mesorectal excision.
Tashiro 2024 [18]	10.1002/jhbp.1388	Laparoscopic and Robotic	Proof-of-concept demonstration	Identification of intrahepatic vascular structures during liver resection.
Frey 2025 [57]	10.1007/s11701-025-02284-7	Laparoscopic and Robotic	Model evaluation	Detecting instruments in robot-assisted abdominal surgeries.
El Moaqet 2025 [58]	10.3390/s25103017	Laparoscopic	Model development and evaluation	To classify and localize surgical tools.
Korndorffer 2020 [59]	10.1097/SLA.0000000000004207	Laparoscopic	Observational study	Assessing critical view of safety and intraoperative events during LC.
Shunjin Ryu 2023 [19]	10.1007/s11605-023-05819-1	Laparoscopic	Prospective observational study	Recognition of nerves during colorectal surgery.
Park 2020 [60]	10.3748/wjg.v26.i44.6945	Laparoscopic	Feasibility study	Analysis of microperfusion for predicting anastomotic complications in laparoscopic colorectal cancer surgery.
Ryu 2024 [61]	10.1007/s00464-023-10524-w	Laparoscopic	Model development	Recognition and visualization of major blood vessels during laparoscopic right hemicolectomy.
Zygomalas 2024 [62]	10.1177/15533506241226502	Laparoscopic	Feasibility study	Recognition of anatomical landmarks and tools in TAPP hernia repair.
Mita 2024 [63]	10.1007/s10029-024-03223-5	Laparoscopic	Validation study	Anatomical recognition during TAPP hernia repair.
Horita 2024 [64]	10.1007/s00464-024-10874-z	Laparoscopic	Model development	To detect active intraoperative bleeding during laparoscopic colectomy.
Kinoshita 2024 [65]	10.1007/s00464-024-10939-z	Laparoscopic and Robotic	Validation study	Nerve recognition in rectal cancer surgery.
Takeuchi 2023 [66]	10.1007/s00464-023-09934-7	Laparoscopic	Model development	Landmarks recognition in TAPP hernia repair.
Une 2024 [67]	10.1007/s00464-023-10637-2	Laparoscopic	Feasibility study	Liver vessel recognition during parenchymal dissection in LLR.
Kojima 2023 [68]	10.1097/JS9.0000000000000317	Laparoscopic	Model development	Segmentation of autonomic nerves during colorectal surgery.
Nakanuma 2023 [69]	10.1007/s00464-022-09678-w	Laparoscopic	Clinical feasibility study	Detecting landmarks during laparoscopic cholecystectomy.
Loukas 2022 [70]	10.1002/rcs.2445	Laparoscopic	Model development	To classify vascularity of gallbladder wall.
Endo 2023 [71]	10.1007/s00464-023-10224-5	Laparoscopic	Prospective experimental study	Anatomical landmark identification during laparoscopic cholecystectomy.
Fried 2024 [72]	10.1097/SLA.0000000000006377	Laparoscopic	Implementation study	Monitoring superior vena cava in laparoscopic cholecystectomy.
Mascagni 2022 [73]	10.1097/SLA.0000000000004351	Laparoscopic	Model development	To segment hepatocystic anatomy.
Fujinaga 2023 [74]	10.1007/s00464-023-10097-8	Laparoscopic	Clinical feasibility study	Landmark recognition to reduce bile duct injury.
Kawamura 2023 [75]	10.1007/s00464-023-10328-y	Laparoscopic	Model development	Automatically score CVS criteria.
Tokuyasu 2021 [76]	10.1007/s00464-020-07548-x	Laparoscopic	Model development and validation	Anatomical landmarks during laparoscopic cholecystectomy.
Zhang 2024 [77]	10.3760/cma.j.cn441530-20240125-00041	Laparoscopic	Model development and validation	Detect organs and instruments in laparoscopic radical gastrectomy.
Skill Assessment and Training				
Ortenzi 2023 [78]	10.1007/s00464-023-10375-5	Laparoscopic	Model development and validation	Surgical steps recognition in totally extraperitoneal (TEP) inguinal hernia repairs.
Wu 2024 [20]	10.1097/JS9.0000000000001798	Laparoscopic	Multicenter randomized controlled trial	AI-based surgical coaching program for laparoscopic cholecystectomy.
Belmar 2023 [79]	10.1007/s00464-022-09576-1	Laparoscopic	Validation study	Assessing basic laparoscopic simulation training exercises.
Halperin 2023 [21]	10.1007/s11548-023-02963-6	Laparoscopic	Prospective validation study (simulator-based)	Automated feedback on intracorporeal suture performance.
Chen 2023 [22]	10.1007/s11701-023-01713-9	Robotic	Prospective observational study (simulator-based)	To evaluate robotic suturing skills.
Fawaz 2019 [80]	10.1007/s11548-019-02039-4	Robotic	Retrospective model development	Classify robotic surgical skill levels and personalized feedback.
Nguyen 2019 [81]	10.1016/j.cmpb.2019.05.008	Robotic	Model development	Objective surgical skill assessment.
Wang 2025 [82]	10.1109/EMBC.2018.8512575	Robotic	Model development	Recognize surgical tasks and assess surgeon skill levels in robot-assisted training.
Funke 2019 [83]	10.1007/s11548-019-01995-1	Robotic	Model development	Surgical skill assessment system.
Partridge 2014 [84]	10.1089/lap.2014.0015	Laparoscopic	Tool development and validation	To track instrument movement for performance feedback in laparoscopic simulators.
Dereathe 2025 [85]	10.1038/s41597-025-04588-7	Laparoscopic	Dataset development and evaluation	To assess quality of field exposure in sleeve gastrectomy.
Bogar 2024 [86]	10.1038/s41598-024-67435-6	Laparoscopic	Simulation study	Custom VR simulator and AI-based peg transfer evaluator.
Matsumoto 2024 [87]	10.1038/s41598-024-63388-y	Laparoscopic	Kinematic analysis	To analyze kinematic differences in laparoscopic distal gastrectomy by surgical skill level.
Gilliani 2024 [88]	10.1016/j.jss.2024.07.103	Robotic	Skill classification study	To distinguish expert, intermediate, and novice surgeons in robotic right colectomy.
Yang 2023 [89]	10.1007/s00464-022-09781-y	Robotic	Algorithm validation	Surgical skill grading in colorectal robotic surgery.
Caballero 2024 [90]	10.1007/s11548-024-03218-8	Robotic	Observational study	To predict surgeon stress levels during robotic surgery using ergonomic and physiological parameters.
Yanik 2024 [91]	10.1007/s44186-023-00223-4	Laparoscopic	Learning curve analysis	To predict surgical skill acquisition through self-supervised video-based learning.
Nakajima 2024 [92]	10.1007/s00464-024-11208-9	Laparoscopic	Retrospective analysis	To validate automated surgical skill assessment in sigmoidectomy.
Yamazaki 2022 [93]	10.1007/s11605-021-05161-4	Laparoscopic	Retrospective analysis	To compare surgical device usage patterns during laparoscopic gastrectomy by surgeon skill level.
Allen 2010 [94]	10.1007/s00464-009-0556-6	Laparoscopic	Experimental study	Automatic evaluation of laparoscopic skills.
Fukuta 2024 [95]	10.1007/s11548-024-03253-5	Laparoscopic	Development study	To assess laparoscopic surgical skills.
Moglia 2022 [96]	10.1007/s00464-021-08999-6	Robotic	Observational study	To predict proficiency acquisition rates in robotic-assisted surgery trainees.
Ju 2025 [97]	10.1007/s00464-025-11730-4	Laparoscopic	Development study	Automatic gesture recognition model for laparoscopic training.
Cruz 2025 [98]	10.1007/s44186-025-00355-9	Laparoscopic	Validation study	Skill assessment in laparoscopic simulation training.
Chen 2024 [99]	10.1097/JS9.0000000000000975	Laparoscopic	Development study	Evaluate surgical skills based on surgical gestures.
Erlich-Feingold 2025 [100]	10.1007/s00464-025-11715-3	Laparoscopic	Development study	To classify basic laparoscopic skills (precision cutting tasks).
Power 2025 [101]	10.1038/s41598-025-96336-5	Laparoscopic	Development study	Evaluate laparoscopic surgical skill across expertise levels.
Alonso-Silverio 2018 [102]	10.1177/1553350618777045	Laparoscopic	Development and evaluation study	Affordable laparoscopic trainer with AI, CV, and AR for online surgical skills assessment.
Pan 2011 [103]	10.1002/rcs.399	Laparoscopic	Development study	Laparoscopic rectal surgery training.
Ershad 2019 [104]	10.1007/s11548-019-01920-6	Robotic	Development study	Automatic stylistic behaviour recognition using joint position data in robotic surgery.
Kowalewski 2019 [105]	10.1007/s00464-019-06667-4	Laparoscopic	Experimental study	Skill level assessment and phase detection.
St John A 2024 [106]	10.1007/s00464-024-11068-3	Laparoscopic	Validation study	AI-powered mobile game for learning safe dissection in LC.
Yen 2025 [107]	10.1007/s00464-025-11663-y	Laparoscopic	Development and validation study	To assess surgical actions and develop automated models for competency assessment in LC.
Nakajima 2025 [108]	10.1007/s00423-025-03641-8	Laparoscopic	Retrospective multicenter study	To assess surgical dissection skill.
Igaki 2023 [109]	10.1001/jamasurg.2023.1131	Laparoscopic	Development and validation study	To recognize standardized surgical fields and assess skill.
Smith 2022 [110]	10.1007/s11701-021-01284-7	Robotic	Validation study	Classification of surgical skill level.
Workflow Recognition and Intraoperative Guidance				
Loukas 2024 [111]	10.1002/rcs.2632	Laparoscopic	Model development	To predict the remaining surgery duration.
Wagner 2023 [112]	10.1016/j.media.2023.102770	Laparoscopic	Multicenter dataset development and benchmark study	To evaluate generalizability of workflow, instrument, action, and skill recognition models in laparoscopic cholecystectomy.
Zhang 2023 [113]	10.1007/s11548-022-02811-z	Laparoscopic and Robotic	Model development and validation	Automatic surgical workflow recognition.
Park 2023 [114]	10.1016/j.compbiomed.2023.107453	Laparoscopic	Multimodal model development	Surgical phase recognition by integrating tool interaction and visual modality in laparoscopic surgery.
Twinanda 2019 [115]	10.1109/TMI.2018.2878055	Laparoscopic	Model development	To estimate remaining surgery duration intraoperatively.
Zang 2023 [116]	10.3390/bioengineering10060654	Robotic	Model comparison	Surgical phase recognition in RALIHR.
Cartucho 2024 [117]	10.1016/j.media.2023.102985	Laparoscopic	Model development and validation	To track soft tissue movement during laparoscopic procedures.
Zheng 2022 [118]	10.1007/s11548-022-02568-5	Laparoscopic	Experimental study	To detect stress in surgical motion.
Zhai 2024 [119]	10.1007/s11548-023-03027-5	Laparoscopic	Model development and validation	Surgical phase recognition in gastric cancer surgery.
Takeuchi 2022 [23]	10.1007/s10029-022-02621-x	Laparoscopic	Model development	Phase recognition in TAPP and assess links to surgical skill.
Hashimoto 2020 [23]	10.1097/SLA.0000000000003460	Laparoscopic	Algorithm development and validation	To identify operative steps in laparoscopic sleeve gastrectomy.
You 2024 [120]	10.1007/s00464-024-10916-6	Laparoscopic	Model development and validation	Automated surgical phase recognition in laparoscopic pancreaticoduodenectomy.
Takeuchi 2023 [24]	10.1007/s00464-023-09924-9	Robotic	Model development	Surgical phases recognition and prediction of complexity in robotic distal gastrectomy
Zheng 2023 [121]	10.1002/rcs.2449	Laparoscopic	Model development	Better intraoperative field visualization.
Dayan 2024 [122]	10.1007/s11695-023-07043-x	Laparoscopic	External validation study	AI model for identifying sleeve gastrectomy safety milestones.
Kitaguchi 2020 [123]	10.1016/j.ijsu.2020.05.015	Laparoscopic	Model development	AI to recognize surgical phase, action and tools.
Yoshida 2024 [124]	10.1007/s00423-024-03411-y	Laparoscopic	Model development and evaluation	Surgical step recognition in laparoscopic distal gastrectomy.
Fer 2023 [125]	10.1007/s00464-023-09870-6	Laparoscopic	Model development	Step labelling in Roux-en-Y gastric bypass.
Liu 2023 [126]	10.1097/JS9.0000000000000559	Robotic	Model development	Workflow recognition model for robotic left lateral sectionectomy.
Khojah 2025 [127]	10.1007/s00464-025-11694-5	Laparoscopic	Model development and intraoperative validation	Real-time ureter localization during laparoscopic sigmoidectomy.
Lavanchy 2024 [128]	10.1007/s11548-024-03166-3	Laparoscopic	Dataset creation and benchmarking	Improving AI model generalizability.
Komatsu 2024 [129]	10.1007/s10120-023-01450-w	Laparoscopic	Model development and feasibility study	Phase recognition for laparoscopic distal gastrectomy.
Sasaki 2022 [130]	10.1016/j.ijsu.2022.106856	Laparoscopic	Model development	Automated surgical step identification in laparoscopic hepatectomy.
Madani 2022 [131]	10.1097/SLA.0000000000004594	Laparoscopic	Model development and validation	Intraoperative guidance by identifying safe/dangerous zones and anatomical landmarks in cholecystectomy.
Cheng 2022 [132]	10.1007/s00464-021-08619-3	Laparoscopic	Model development and multicenter validation	Phase recognition in laparoscopic cholecystectomy.
Golany 2022 [133]	10.1007/s00464-022-09405-5	Laparoscopic	Model development	Surgical phase recognition.
Shinozuka 2022 [134]	10.1007/s00464-022-09160-7	Laparoscopic	Model development and validation	Surgical phase recognition in laparoscopic cholecystectomy.
Laplante 2022 [25]	10.1007/s00464-022-09439-9	Laparoscopic	Model validation	Identifying safe and dangerous zones in left colectomy.
Surgical decision support and outcome prediction				
López 2024 [25]	10.1007/s00464-024-10681-6	Laparoscopic	Retrospective multicenter study	To predict surgical complexity and postoperative outcomes in laparoscopic liver surgery.
Masum 2022 [135]	10.1007/s12672-022-00472-7	Laparoscopic and robotic	Retrospective study	Prediction of LOS, readmission, and mortality.
López 2022 [136]	10.1007/s11605-022-05398-7	Laparoscopic	Retrospective multi-institutional cohort study	To identify factors associated with successful initial repair of IBDI and predict the success of definitive repair using AI.
Cai 2023 [137]	10.3748/wjg.v29.i3.536	Laparoscopic	Retrospective study	Prediction of the number of stapler cartridges needed to avoid high-risk anastomosis.
Dayan 2024 [138]	10.1007/s00464-024-10847-2	Laparoscopic	Retrospective observational validation study	Grading intraoperative complexity and safety adherence in laparoscopic appendectomy.
Arpaia 2022 [139]	10.1038/s41598-022-16030-8	Laparoscopic	Development and validation study	Assessment of perfusion quality during laparoscopic colorectal surgery.
Gillani 2024 [140]	10.1016/j.surg.2024.08.015	Robotic	Feasibility study	Performance indicators during robotic proctectomy.
Emile 2024 [141]	10.1007/s13304-024-01915-2	Laparoscopic and Robotic	Retrospective case–control study	Predict of conversion from minimally invasive (laparoscopic or robotic) to open colectomy.
Wang 2024 [26]	10.3748/wjg.v30.i43.4669	Laparoscopic	Multicenter retrospective cohort study development	Risk of postoperative complications in laparoscopic radical gastrectomy.
Velmahos 2023 [142]	10.1177/00031348231167397	Laparoscopic	Comparative model analysis	Morbidity prediction after laparoscopic colectomy.
Jo 2025 [143]	10.1016/j.hpb.2025.02.016	Laparoscopic	Retrospective study	Predictors of conversion to open surgery in laparoscopic repeat liver resection.
Li 2025 [144]	10.1016/j.surg.2024.108999	Laparoscopic	Retrospective study	Estimate the risk of duodenal stump leakage in laparoscopic gastrectomy.
Lippenberger 2024 [145]	10.1007/s00384-024-04593-z	Laparoscopic	Retrospective single-center cohort study	To predict procedure duration of laparoscopic sigmoid resections.
Zhou 2024 [146]	10.1016/j.heliyon.2024.e26580	Laparoscopic	Retrospective predictive model	Predicting postoperative intestinal obstruction in laparoscopic colorectal cancer surgery.
Augmented reality and navigation				
Aoyama 2024 [28]	10.1007/s00464-024-11160-8	Laparoscopic	Feasibility study	To identify anatomical landmarks associated with postoperative pancreatic fistula during laparoscopic gastrectomy.
Du 2022 [147]	10.1186/s12893-022-01585-0	Laparoscopic	System development and preclinical evaluation	Intraoperative navigation system.
Kasai 2024 [148]	10.7759/cureus.48450	Laparoscopic	System development	Mapping for portal segment identification in laparoscopic liver surgery.
Ryu 2024 [149]	10.1007/s10895-024-04030-y	Laparoscopic	Feasibility study	Combining AI and NIR fluorescence for anatomical recognition during colorectal surgery.
Garcia-Granero 2023 [150]	10.1016/j.ciresp.2022.10.023	Laparoscopic	Case report	3D image reconstruction system in mesocolic excision and lymphadenectomy.
Guan 2023 [27]	10.1007/s11548-023-02846-w	Laparoscopic	Laboratory/technical study	Mapping using stereo 3D laparoscopy and CT registration for liver resection.
Ali 2024 [151]	10.1016/j.media.2024.103371	Laparoscopic	Challenge dataset + algorithm development	Automate landmark detection for CT-laparoscopic image.
Robu 2017 [152]	10.1007/s11548-017-1584-7	Laparoscopic	Proof-of-concept study	View-planning strategy to improve AR registration using CT and laparoscopic video.
Wei 2022 [153]	10.1109/TBME.2022.3195027	Laparoscopic	Technical/methodological study	3D localization method for anatomical navigation during MIS.
Nicolau 2005 [154]	10.1007/11566489_4	Laparoscopic	Feasibility study	Enhance depth perception.
Calinon 2014 [155]	10.1016/j.cmpb.2013.12.015	Laparoscopic	Technical development	Skill transfer interfaces for soft robotic using context-aware learning.
Image enhancement				
Zheng 2022 [156]	10.1007/s11548-022-02777-y	Laparoscopic	Algorithm development	Remove visual impairments
Cheng 2022 [157]	10.1155/2022/2752444	Robotic	Comparative experimental study	Image edge detection algorithm in robotic gastric surgery.
Akbari 2009 [29]	10.1109/IEMBS.2009.5333766	Laparoscopic	Prospective evaluation	Artery detection in laparoscopic cholecystectomy.
Katic 2013 [158]	10.1016/j.compmedimag.2013.03.003	Laparoscopic	Conceptual/methodological article	Reduce information overload in surgery.
Beyersdorffer 2021 [159]	10.1515/BMT-2020-0106	Laparoscopic	Feasibility study	Detect the presence of dissecting tool within camera field.
Salazar-Colores 2022 [160]	10.24875/CIRU.20000951	Laparoscopic	Algorithm development	Remove surgical smoke.
Wagner 2021 [30]	10.1007/s00464-021-08509-8	Laparoscopic	Prospective experimental study	Autonomous camera-guiding robot.
He 2025 [161]	10.1007/s00464-025-11693-6	Laparoscopic	Prospective feasibility study	Intraoperative perfusion assessment in colorectal surgery.
Surgeon perception, preparedness, and attitudes				
Acosta 2025 [31]	10.1016/j.ciresp.2024.12.003	Robotic	Survey study	Evaluate Spanish surgeons’ knowledge, attitudes, and preparedness toward Digital Surgery and AI, comparing robotic vs. non-robotic users.
Luense 2023 [162]	10.1007/s00423-023-03134-6	Laparoscopic	Survey study	Survey of German surgeons to identify limitations of current laparoscopy and desired AI features in future systems.
Shafiei 2025 [32]	10.1177/00187208241285513	Laparoscopic and Robotic	Experimental study using simulation data	To predict mental workload during surgical tasks.

**Table 3:** Risk of bias assessment.

Category	Low Risk	Moderate Risk	High Risk	External Validation	Multicenter Design
Object/Structure Detection	28.0%	60.0%	12.0%	12.0%	18.0%
Skill Assessment & Training	21.2%	66.7%	12.1%	9.1%	15.2%
Workflow Recognition & Guidance	35.0%	55.0%	10.0%	25.0%	30.0%
Decision Support & Outcome Prediction	31.8%	59.1%	9.1%	27.3%	40.9%
Augmented Reality & Navigation	40.0%	50.0%	10.0%	33.3%	46.7%
Image Enhancement	20.0%	60.0%	20.0%	10.0%	20.0%
Perception/Preparedness	100.0%	0.0%	0.0%	—	100.0%
Global	31.4%	58.0%	10.6%	20.0%	27.1%

**Table 4:** Summary of performance metrics and validation characteristics by thematic category. Median or representative values are shown where available.

Category	Typical Metrics Reported	Median/Representative Values	External Validation (%)	Clinical or Ex Vivo Validation (%)	Multicenter Studies (%)	Main Limitations
Object/Structure Detection	Accuracy, IoU, Dice, F1-score	Accuracy 0.87 (range 0.72–0.96); Dice 0.83 (0.68–0.92)	12%	48%	18%	Lack of standard annotation, limited generalizability
Surgical Skill Assessment & Training	Accuracy, F1-score, correlation with human rating	Accuracy 0.84 (0.70–0.93); r = 0.71 with expert scoring	8%	15%	15%	Mostly simulation-based, subjective reference
Workflow Recognition & Intraoperative Guidance	Phase accuracy, F1-score, mAP	Accuracy 0.89 (0.74–0.95); mAP 0.82 (0.65–0.91)	25%	30%	30%	Variability in labelling and phase definitions
Decision Support & Outcome Prediction	AUC, sensitivity, specificity	AUC 0.86 (0.73–0.93); sens. 0.82 (0.70–0.91)	27%	40%	41%	Retrospective data, poor model interpretability
Augmented Reality & Navigation	Registration error (TRE, mm), overlay latency	TRE 3.5 mm (2.4–5.8); latency < 150 ms	33%	66%	45%	Limited intraoperative usability evaluation
Image Enhancement	PSNR, SSIM, FPS	PSNR 31.5 dB (28–37); SSIM 0.91 (0.84–0.96)	10%	30%	20%	Preclinical data, no clinical outcome measures
Perception, Preparedness & Attitudes	Descriptive survey statistics	Positive perception ≥ 80%; low formal AI training (≤20%)	—	—	100%	Self-reporting bias, uneven response rate

IoU: intersection over union; Dice: Dice similarity coefficient; F1: F1-score; AUC: area under the receiver operating characteristic curve; TRE: target registration error; PSNR: peak signal-to-noise ratio; SSIM: structural similarity index; FPS: frames per second.

## Data Availability

The raw data supporting the conclusions of this article will be made available by the authors on request.

## Supplementary Materials

The following supporting information can be downloaded at: https://www.mdpi.com/article/10.3390/jpm15110562/s1, Table S1: Study-level Risk of Bias Assessment; Table S2: Study-level AI Model Characteristics and Performance Metrics.

## Abbreviations

The following abbreviations are used in this manuscript:

AI	artificial intelligence;
CNN	Convolutional Neural Network;
AR	Augmented Reality;
TME	Total Mesorectal Excision;
TaTME	Transanal Total Mesorectal Excision;
CVS	Critical View of Safety;
LLR	Laparoscopic Liver Resection;
TAPP	Transabdominal Preperitoneal hernia repair;
LC	Laparoscopic Cholecystectomy;
TEP	Totally Extraperitoneal;
VR	Virtual Reality;
RALIHR	Robotic-Assisted Laparoscopic Inguinal Hernia Repair;
NIR	Near-Infrared;
MIS	Minimally Invasive Surgery;
IoU	intersection over union;
Dice	Dice similarity coefficient;
F1	F1-score;
AUC	area under the receiver operating characteristic curve;
TRE	target registration error;
PSNR	peak signal-to-noise ratio;
SSIM	structural similarity index;
FPS	frames per second.
