# Supplementary material for: Artificial Intelligence in Gastrointestinal Surgery: A Systematic Review of Its Role in Laparoscopic and Robotic Surgery
Source: J Pers Med. 2025 Nov 19;15(11):562. doi: 10.3390/jpm15110562 (PMC12653678; doi:10.3390/jpm15110562)
Supplement: Supplementary file 1 [file jpm-15-00562-s001.zip › jpm-3866058-supplementary.pdf]

Supplementary Materials

1. Supplementary Table S1: Study-level Risk of Bias Assessment
2. Supplementary Table S2: Study-level AI Model Characteristics and Performance Metrics
3. References of all included articles

	First Author (Year)	DOI	Type of Surgery	Bias-Assessment Tool	Overall Risk of Bias
**Object Or Structure Detection**					
[1]	Khalid (2023)	10.1007/s00464-023-10403-4	Laparoscopic cholecystectomy	QUADAS-AI	Moderate
[2]	Ward (2022)	10.1007/s00464-022-09009-z	Laparoscopic cholecystectomy	QUADAS-AI	Moderate
[3]	Orimoto (2024)	10.1007/s00464-024-11514-2	Laparoscopic trocar insertion	MINORS	Low
[4]	Kolbinger (2023)	10.1097/JS9.0000000000000595	Laparoscopic colorectal	QUADAS-AI	Low
[5]	Sato (2022)	10.1007/s00595-022-02508-5	Laparoscopic / Robotic gastrectomy	MINORS	Moderate
[6]	Igaki (2022)	10.1097/DCR.0000000000002393	Laparoscopic colorectal	MINORS	Moderate
[7]	Jearanai (2023)	10.1007/s00464-023-10309-1	Laparoscopic (abdominal wall access)	MINORS	Low
[8]	Oh (2024)	10.1038/s41598-024-73434-4	Laparoscopic liver donor hepatectomy	QUADAS-AI	Low
[9]	Benavides (2024)	10.3390/s24134191	Laparoscopic / Robotic (tool tracking)	MINORS	Low
[10]	Gazis (2022)	10.3390/bioengineering9120737	Laparoscopic training / simulation	AXIS	Moderate
[11]	Tomioka (2023)	10.21873/anticanres.16725	Laparoscopic hepatectomy	MINORS	Moderate
[12]	Cui (2021)	10.1155/2021/5578089	Laparoscopic inguinal hernia repair	QUADAS-AI	Low
[13]	Memida (2023)	10.1109/EMBC40787.2023.10341025	Laparoscopic (instrument tracking)	MINORS	Moderate
[14]	Nwoye (2019)	10.1007/s11548-019-01958-6	Laparoscopic (multi-procedure tool tracking)	QUADAS-AI	Low

[15]	Jurosch (2024)	10.1007/s11548-024-03220-0	Laparoscopic (extra-abdominal trocar/instrument tracking)	MINORS	Low
[16]	Sánchez-Brizuela (2022)	10.3390/s22145180	Laparoscopic (gauze detection and segmentation)	MINORS	Moderate
[17]	Lai (2023)	10.1007/s10439-022-03033-9	Laparoscopic colorectal	QUADAS-AI	Low
[18]	Ehrlich (2022)	10.3390/s22155808	Robotic/laparoscopic (electrosurgical cautery detection)	ROBINS-I	Moderate
[19]	Carstens (2023)	10.1038/s41597-022-01719-2	Laparoscopic / Robotic rectal resections	MINORS	Low
[20]	Yin (2023)	10.1016/j.neunet.2023.11.055	Laparoscopic (TaTME procedure image segmentation)	MINORS	Low
[21]	Tashiro (2024)	10.1002/jhbp.1422	Laparoscopic cholecystectomy	MINORS	Moderate
[22]	Petracchi (2024)	10.1016/j.gassur.2024.03.018	Laparoscopic cholecystectomy	MINORS	Low
[23]	Schnelldorfer (2024)	10.1097/SLA.0000000000006294	Staging laparoscopy for gastrointestinal cancers	QUADAS-AI	Moderate
[24]	Kitaguchi (2023)	10.1093/bjs/znad249	Laparoscopic colorectal surgery	QUADAS-AI	Moderate
[25]	Chen (2025)	10.1093/bjsopen/zrae158	Laparoscopic radical gastrectomy (D2 lymphadenectomy)	QUADAS-AI	Low
[26]	Han (2025)	10.1097/DCR.0000000000003547	Laparoscopic total mesorectal excision	MINORS	Moderate
[27]	Yoshihiko (2023)	10.1002/jhbp.1388	Laparoscopic liver resection	MINORS	Moderate
[28]	Frey (2025)	10.1007/s11701-025-02284-7	Robotic and laparoscopic abdominal surgery	QUADAS-AI	Low
[29]	ElMoaqet (2025)	10.3390/s25103017	Laparoscopic cholecystectomy (tool classification/localization, Cholec80)	MINORS	Moderate
[30]	Korndorffer (2020)	10.1097/SLA.0000000000004207	Laparoscopic cholecystectomy	QUADAS-AI	Moderate
[31]	Ryu (2023)	10.1007/s11605-023-05819-1	Laparoscopic left colorectal surgery	MINORS	Moderate
[32]	Park (2020)	10.3748/wjg.v26.i44.6945	Laparoscopic colorectal cancer surgery	MINORS	Moderate
[33]	Ryu (2024)	10.1007/s00464-023-10524-w	Laparoscopic right hemicolectomy	QUADAS-AI	Low

[34]	Zygomalas (2024)	10.1177/155335062412265 02	Laparoscopic TAPP inguinal hernia repair	MINORS	Moderate
[35]	Mita (2025)	10.1007/s10029-024-03223-5	Laparoscopic TAPP inguinal hernia repair	QUADAS-AI	Moderate
[36]	Tomioka (2024)	10.21873/anticanres.16725	Laparoscopic hepatectomy	MINORS	Moderate
[37]	Horita (2024)	10.1007/s00464-024-10874-z	Laparoscopic colectomy (active bleeding detection)	QUADAS-AI	Moderate
[38]	Kinoshita (2024)	10.1007/s00464-024-10939-z	Laparoscopic / robot-assisted rectal cancer surgery (nerve recognition)	MINORS	Moderate
[39]	Takeuchi (2023)	10.1007/s00464-023-09934-7	Laparoscopic TAPP inguinal hernia repair (CVMPO landmark identification)	MINORS	Moderate
[40]	Une (2024)	10.1007/s00464-023-10637-2	Laparoscopic liver resection	QUADAS-AI	Low
[41]	Kojima (2023)	10.1097/JS9.0000000000000317	Laparoscopic colorectal surgery (autonomic nerve segmentation)	QUADAS-AI	Moderate
[42]	Nakanuma (2022)	10.1007/s00464-022-09678-w	LC (AI intraop. landmark detection; feasibility trial)	QUADAS-AI	Moderate
[43]	Loukas (2022)	10.1002/rcs.2445	LC (gallbladder wall vascularity classification from images)	MINORS	Moderate
[44]	Endo (2023)	10.1007/s00464-023-10224-5	LC (impact of AI on landmark recognition; user study)	MINORS	Moderate
[45]	Fried (2024)	10.1097/SLA.0000000000006377	LC (CVS monitoring via surgical-intelligence platform)	ROBINS-I	Moderate
[46]	Mascagni (2022)	10.1097/SLA.0000000000004351	LC (CVS detection with deep learning)	QUADAS-AI	Low
[47]	Fujinaga (2023)	10.1007/s00464-023-10097-8	Laparoscopic cholecystectomy (AI for intraoperative landmark and CVS support)	QUADAS-AI	Moderate
[48]	Kawamura (2023)	10.1007/s00464-023-10328-y	Laparoscopic cholecystectomy (real-time critical view of safety assessment)	QUADAS-AI	Low
[49]	Tokuyasu (2021)	10.1007/s00464-020-07548-x	Laparoscopic cholecystectomy (intraoperative anatomical landmark identification)	QUADAS-AI	Moderate
[50]	Zhang (2024)	10.3760/cma.j.cn441530-20240125-00041	Radical laparoscopic gastrectomy for gastric cancer (multicenter instrument and organ recognition)	QUADAS-AI	Low
**Skill Assessment And Training**					

[51]	Ortenzi (2023)	10.1007/s00464-023-10375-5	Laparoscopic TEP inguinal hernia repair (automatic workflow recognition)	QUADAS-AI	Moderate
[52]	Wu (2024)	10.1097/JS9.0000000000001798	Laparoscopic cholecystectomy (AI-based surgical coaching RCT)	ROBINS-I	Low
[53]	Belmar (2023)	10.1007/s00464-022-09576-1	Laparoscopic simulation (automated skill evaluation)	MINORS	Low
[54]	Halperin (2024)	10.1007/s11548-023-02963-6	Laparoscopic simulation (intracorporeal suturing skill analysis)	MINORS	Moderate
[55]	Chen (2023)	10.1007/s11701-023-01713-9	Robotic suturing (dry-lab skill assessment using trajectory monitoring)	MINORS	Moderate
[56]	Ismail Fawaz (2019)	10.1007/s11548-019-02039-4	Robotic training tasks (JIGSAWS) – kinematic skill classification	MINORS	Moderate
[57]	Nguyen (2019)	10.1016/j.cmpb.2019.05.008	Open/robotic training (IMU platform) + JIGSAWS generalization	MINORS	Moderate
[58]	Wang (2018)	10.1109/EMBC.2018.8512575	Robotic training (JIGSAWS) – motion analytics (SATR-DL)	MINORS	Moderate
[59]	Funke (2019)	10.1007/s11548-019-01995-1	Robotic training (JIGSAWS) – video-only skill assessment	MINORS	Moderate
[60]	Partridge (2014)	10.1089/lap.2014.0015	Laparoscopic box trainer (InsTrac) – construct validity	MINORS	Moderate
[61]	Derathé (2025)	10.1038/s41597-025-04588-7	Dataset descriptor (LapEx: sleeve gastrectomy multimodal annotations)	Not applicable (dataset)	—
[62]	Bogar (2024)	10.1038/s41598-024-67435-6	Peg-transfer training (VR simulator + AI assessment)	MINORS	Moderate
[63]	Matsumoto (2024)	10.1038/s41598-024-63388-y	Laparoscopic distal gastrectomy (video-based skill evaluation via instrument ID)	MINORS	Moderate
[64]	Gillani (2024)	10.1016/j.jss.2024.07.103	Robotic right colectomy (objective performance indicators by ML)	MINORS	Moderate
[65]	Yang (2023)	10.1007/s00464-022-09781-y	Robotic colorectal surgery (AI skill analysis vs GEARS)	MINORS	Moderate
[66]	Caballero (2024)	10.1007/s11548-024-03218-8	Robotic surgery (EDA-based stress prediction with ML)	MINORS	Moderate
[67]	Yanik (2024)	10.1007/s44186-023-00223-4	Laparoscopic suturing simulation (FLS intracorporeal knot-tying)	MINORS	Moderate
[68]	Nakajima (2024)	10.1007/s00464-024-11208-9	Retrospective video study; automated phase recognition to infer skill (lap. sigmoidectomy)	MINORS (feasibility/experimental)	Moderate

[69]	Yamazaki (2022)	10.1007/s11605-021-05161-4	Retrospective analysis of device usage in lap. gastrectomy vs surgeon skill level (YOLOv3 detection)	ROBINS-I (non-randomized comparative between qualified vs non-qualified)	Moderate
[70]	Allen (2009)	10.1007/s00464-009-0556-6	Simulator study (peg transfer/pass rope/cap needle); SVM-based competency classification	MINORS	Moderate
[71]	Fukuta (2025)	10.1007/s11548-024-03253-5	Simulator feasibility: forceps tracking with DeepLabCut to quantify skills	MINORS	Moderate–High
[72]	Moglia (2022)	10.1007/s00464-021-08999-6	Robotic simulator proficiency prediction (DNN ensemble)	MINORS	Moderate
[73]	Ju (2025)	10.1007/s00464-025-11730-4	Laparoscopic peg transfer skill evaluation using gesture recognition	MINORS	Low
[74]	Cruz (2025)	10.1007/s44186-025-00355-9	Laparoscopic simulation training (AI-assisted video-based assessment)	MINORS	Low
[75]	Chen (2024)	10.1097/JS9.0000000000000975	Laparoscopic cholecystectomy – video-based skill classification using Surgestures (multicentre, 5 hospitals)	MINORS	Moderate
[76]	Erlich-Feingold (2025)	10.1007/s00464-025-11715-3	Laparoscopic simulation – FLS “precision cutting” (video AI classifier, single-centre)	MINORS	Moderate
[77]	Power (2025)	10.1038/s41598-025-96336-5	Laparoscopic simulation – new LSPD dataset + 3DCNN skill classification	MINORS	Moderate
[78]	Alonso-Silverio (2018)	10.1177/1553350618777045	Low-cost laparoscopic box trainer + ANN for skill assessment	MINORS	Moderate
[79]	Belmar (2022)	10.1007/s00464-022-09576-1	Basic laparoscopic skills simulation (peg transfer & bean drop tasks)	MINORS	Moderate
[80]	Pan (2010)	10.1002/rcs.399	Laparoscopic rectal surgery training – human phantom model	MINORS	Moderate
[81]	Ershad (2019)	10.1007/s11548-019-01920-6	Robotic surgery – automatic stylistic behavior recognition using joint kinematics	MINORS	Moderate
[82]	Kowalewski (2020)	10.1007/s00464-019-06667-4	Laparoscopic training dataset review – AI and skill assessment overview	AXIS	Low
[83]	St John (2024)	10.1007/s00464-024-11068-3	Laparoscopic cholecystectomy – AI mobile game for safe dissection training	MINORS	Moderate
[84]	Yen (2025)	10.1007/s00464-025-11663-y	Laparoscopic cholecystectomy – automated action recognition and competency assessment	MINORS	Moderate

[85]	Nakajima (2025)	10.1007/s00423-025-03641-8	Laparoscopic colorectal surgery – AI assessment of tissue dissection efficiency	ROBINS-I	Moderate
[86]	Igaki (2023)	10.1001/jamasurg.2023.1131	Laparoscopic sigmoid colon resection – automatic skill assessment based on surgical field standardization	ROBINS-I	Moderate
[87]	Smith (2021)	10.1007/s11701-021-01284-7	Simulation-based robotic training (Ring & Rail and Suture Sponge exercises)	MINORS	Moderate
**Workflow Recognition And Intraoperative Guidance**					
[88]	Loukas (2024)	10.1002/rcs.2632	Laparoscopic cholecystectomy – prediction of remaining surgery duration (RSD) from visual features	QUADAS-AI	Moderate
[89]	Wagner (2023)	10.1016/j.media.2023.102770	Multicenter LC benchmark (HeiChole): phase, action, instrument and skill recognition tasks	Benchmark dataset	Moderate
[90]	Zhang (2023)	10.1007/s11548-022-02811-z	LC (video-based workflow recognition, lap + robotic)	QUADAS-AI	Moderate
[91]	Park (2023)	10.1016/j.compbiomed.2023.107453	Laparoscopic distal gastrectomy + VR simulator – multimodal phase recognition	QUADAS-AI	Moderate
[92]	Twinanda (2018)	10.1109/TMI.2018.2878055	LC + gastric bypass – annotation-free remaining surgery duration estimation (RSDNet)	QUADAS-AI	Moderate
[93]	Zang (2023)	10.3390/bioengineering10060654	Robotic inguinal hernia repair – phase recognition benchmark (RALIHR)	QUADAS-AI	Moderate
[94]	Cartucho (2024)	10.1016/j.media.2023.102985	Laparoscopic cholecystectomy – soft-tissue tracking benchmark (EndoVis 2022 challenge)	MINORS (adapted for non-comparative designs)	High
[95]	Zheng (2022)	10.1007/s11548-022-02568-5	Simulation-based laparoscopic training – stress detection from kinematics	MINORS (experimental study)	Moderate
[96]	Zhai (2024)	10.1007/s11548-023-03027-5	Laparoscopic gastrectomy – workflow phase recognition	MINORS (retrospective video study)	Moderate
[97]	Takeuchi (2022)	10.1007/s10029-022-02621-x	Laparoscopic inguinal hernia (TAPP) — automatic phase recognition (TeCNO+HMM)	QUADAS-AI	Moderate
[98]	Hashimoto (2019)	10.1097/SLA.0000000000003460	Laparoscopic sleeve gastrectomy — automated operative step recognition	QUADAS-AI	Moderate
[99]	You (2024)	10.1007/s00464-024-10916-6	Laparoscopic pancreaticoduodenectomy — phase recognition model validation	QUADAS-AI	Moderate
[100]	Takeuchi (2023)	10.1007/s00464-023-09924-9	Robotic distal gastrectomy — phase recognition & complexity prediction	QUADAS-AI	Moderate

[101]	Zheng (2023)	10.1002/rcs.2449	Mixed laparoscopic (urology) – assistant camera control, instrument segmentation + OR index	MINORS	Moderate
[102]	Dayan (2024)	10.1007/s11695-023-07043-x	Sleeve gastrectomy – real-world AI platform, safety milestones agreement	QUADAS-AI	Moderate
[103]	Kitaguchi (2020)	10.1016/j.ijsu.2020.05.015	Laparoscopic colorectal – multicenter workflow (phase/action/tools)	QUADAS-AI	Moderate
[104]	Yoshida (2024)	10.1007/s00423-024-03411-y	Laparoscopic distal gastrectomy – step recognition (single-center)	QUADAS-AI	Moderate
[105]	Fer (2023)	10.1007/s00464-023-09870-6	Laparoscopic Roux-en-Y gastric bypass – intraoperative workflow recognition using deep CNNs	QUADAS-AI	Moderate
[106]	Liu (2023)	10.1097/JS9.0000000000000559	Robotic left lateral sectionectomy – multilevel workflow recognition	QUADAS-AI	Low
[107]	Khojah (2025)	10.1007/s00464-025-11694-5	Laparoscopic sigmoidectomy – ureter identification with semantic segmentation	QUADAS-AI	Moderate
[108]	Lavanchy (2024)	10.1007/s11548-024-03166-3	LRYGB – multi-centre	QUADAS-AI	Moderate
[109]	Komatsu (2024)	10.1007/s10120-023-01450-w	Laparoscopic distal gastrectomy – phase recognition + skill assessment	QUADAS-AI	Moderate
[110]	Sasaki (2022)	10.1016/j.ijsu.2022.106856	Laparoscopic hepatectomy – automated step identification (real-time)	QUADAS-AI	Moderate
[111]	Madani (2022)	10.1097/SLA.0000000000004594	Laparoscopic cholecystectomy – semantic segmentation (Go/No-Go + anatomy)	QUADAS-AI	Moderate
[112]	Cheng (2021)	10.1007/s00464-021-08619-3	Laparoscopic cholecystectomy — multi-center phase recognition (CNN + LSTM)	QUADAS-AI	Moderate
[113]	Golany (2022)	10.1007/s00464-022-09405-5	Laparoscopic cholecystectomy — phase recognition across complex cases	QUADAS-AI	Moderate
[114]	Shinozuka (2022)	10.1007/s00464-022-09160-7	Laparoscopic cholecystectomy — phase recognition (CNN; device-oriented workflow)	QUADAS-AI	Moderate
[115]	Laplante (2022)	10.1007/s00464-022-09439-9	Laparoscopic cholecystectomy — Go/No-Go safety-zone segmentation (external validation)	QUADAS-AI	Moderate
**Surgical decision support and outcome prediction**					
[116]	Lopez (2024)	10.1007/s00464-024-10681-6	Laparoscopic liver resections (segments 7–8) – multicenter international study predicting surgical complexity and outcomes with explainable AI	PROBAST (predictive model)	Moderate
[117]	Masum (2022)	10.1007/s12672-022-00472-7	Colorectal cancer surgery – population-based, multi-year national registry	PROBAST	Moderate

			(prediction of LOS, readmission, mortality)		
[118]	Lopez (2022)	10.1007/s11605-022-05398-7	Cholecystectomy (iatrogenic bile duct injury) – nationwide multicenter retrospective cohort (ML-based risk model and decision tree)	PROBAST	Moderate
[119]	Zheng (2023)	10.3748/wjg.v29.i3.536	Laparoscopic low anterior resection – MRI-based DL prediction of multiple stapler firings in DST anastomosis	QUADAS-AI	Low-to-Moderate
[120]	Dayan (2024)	10.1007/s00464-024-10847-2	Laparoscopic appendectomy – single-center real-world implementation of AI-based computer-vision model (Theator Inc.) for complexity grading and safety adherence	QUADAS-AI	Moderate
[121]	Arpaia (2022)	10.1038/s41598-022-16030-8	Laparoscopic colorectal surgery – ICG fluorescence perfusion assessment via ML-based video analysis	QUADAS-AI	Low-to-Moderate
[122]	Gillani (2024)	10.1016/j.surg.2024.08.015	Robotic proctectomy – analysis of objective performance indicators (OPIs) in obese vs non-obese patients	PROBAST	Moderate
[123]	Emile (2024)	10.1007/s13304-024-01915-2	Minimally invasive colectomy – AI-designed predictive calculator for conversion to open surgery (NCDB retrospective study)	PROBAST	Moderate
[124]	Wang (2024)	10.3748/wjg.v30.i43.4669	Laparoscopic radical gastrectomy – AI-driven predictive scoring for post-operative complications (multicenter development & validation)	PROBAST	Moderate
[125]	Velmahos (2023)	10.1177/000313482311673 97	Laparoscopic colectomy – 30-day postoperative morbidity prediction using ML vs logistic regression (NSQIP database)	PROBAST	Moderate
[126]	Jo (2025)	10.1016/j.hpb.2025.02.016	Laparoscopic repeat liver resection – indication and open conversion prediction (Samsung Medical Center)	QUADAS-AI	Low-to-Moderate
[127]	Li (2024)	10.1016/j.surg.2024.108999	Laparoscopic colorectal surgery – multicenter development of an interpretable AI system for intraoperative bleeding prediction	QUADAS-AI	Moderate
[128]	Cai (2023)	10.3748/wjg.v29.i3.536	Colorectal surgery – prediction of multiple stapler firings during laparoscopic low anterior resection (LAR)	PROBAST	Low
[129]	Lippenberger (2024)	10.1007/s00384-024-04593-z	Laparoscopic sigmoid resection for diverticular disease – single-center CT-based model predicting surgery duration	QUADAS-AI	Moderate
[130]	Zhou (2024)	10.1016/j.heliyon.2024.e26580	Laparoscopic colorectal cancer surgery – ML and DL models predicting postoperative ileus (POI)	PROBAST	Moderate
**Augmented reality and navigation**					
[131]	Aoyama (2024)	10.1007/s00464-024-11117-x	Laparoscopic gastrectomy for gastric cancer – AI navigation indicating pancreatic–mesenteric landmarks to prevent postoperative pancreatic fistula	QUADAS-AI	Low-to-Moderate

[132]	Du (2022)	10.1186/s12893-022-01585-0	Laparoscopic pancreatic surgery – development of intraoperative 3D virtual model fusion with real-time laparoscopy (preclinical)	QUADAS-AI	Moderate
[133]	Kasai (2023)	10.7759/cureus.48450	Laparoscopic anatomical liver resection – AR + AI projection mapping of portal segments for intraoperative navigation	QUADAS-AI	Moderate
[134]	Ryu (2024)	10.1007/s10895-024-04030-y	Laparoscopic colorectal surgery – feasibility of simultaneous AI-assisted and NIR fluorescence navigation (ureteral and nerve visualization)	QUADAS-AI	Moderate
[135]	Garcia-Granero (2023)	10.1016/j.cireasp.2022.10.023	Colon cancer – laparoscopic complete mesocolic excision and D3 lymphadenectomy planned with pre-operative 3D CT reconstruction and AI-based vascular mapping (2 cases)	QUADAS-AI	Moderate
[136]	Guan (2023)	10.1007/s11548-023-02846-w	Laparoscopic liver resection – deep-learning 3D–3D registration (CT to intraoperative point cloud)	QUADAS-AI	Moderate
[137]	Ali (2024)	10.1016/j.media.2024.103371	Laparoscopic liver resection – international AR challenge (MICCAI 2022) comparing AI registration algorithms	QUADAS-AI	Moderate
[138]	Robu (2017)	10.1007/s11548-017-1584-7	Laparoscopic liver surgery – intelligent viewpoint selection for CT-to-video registration (simulation + clinical pilot)	QUADAS-AI	Moderate
[139]	Wei (2024)	10.1109/TBME.2022.3195027	Minimally invasive / laparoscopic surgery – learning-based stereo dense scene reconstruction and laparoscope localization for navigation (simulation + ex vivo + in vivo datasets)	QUADAS-AI	Low-to-Moderate
[140]	Nicolaou (2005)	10.1007/11566489_4	Laparoscopic surgery (simulated environment) – visual depth perception enhancement via “invisible shadow” navigation aid	QUADAS-AI	Moderate
[141]	Calinon (2014)	10.1016/j.cmpb.2013.12.015	Flexible laparoscopic robotic platform (STIFF-FLOP project) – human-to-robot skill transfer through learning from demonstration	QUADAS-AI	Moderate
**Image enhancement**					
[142]	Zheng (2023)	10.1007/s11548-022-02777-y	Laparoscopic surgery (mainly cholecystectomy and partial nephrectomy) – AI-based real-time video enhancement system (LVQIS) using GAN and CNN for de-blurring, de-smoke/fogging	QUADAS-AI	Low-to-Moderate
[143]	Cheng (2022)	10.1155/2022/2752444	Da Vinci robotic gastrectomy – AI-based video enhancement using thread image edge detection for intraoperative visualization and surgical outcome comparison	ROBINS-I	Moderate
[144]	Akbari (2009)	10.1109/IEMBS.2009.5333766	Laparoscopic cholecystectomy – intraoperative image-guided artery detection in Calot’s triangle using pulsation-based frame subtraction	QUADAS-AI	Low-to-Moderate

[145]	Katic (2013)	10.1016/j.compmedimag.2013.03.003	Laparoscopic liver and cholecystectomy – ontology-based, context-aware augmented reality system for intraoperative visualization	QUADAS-AI	Moderate
[146]	Beyersdorffer (2021)	10.1515/bmt-2020-0106	Laparoscopic cholecystectomy (training) – detection of adverse events (instrument out of view) using CNN classifiers	QUADAS-AI	Moderate
[147]	Salazar-Colores (2022)	10.24875/CIRU.20000951	Laparoscopic surgery – AI-based removal of surgical smoke via GAN + dark channel prior hybrid model	QUADAS-AI	Low-to-Moderate
[148]	Wagner (2021)	10.1007/s00464-021-08509-8	Minimally invasive rectal resection – self-learning robotic system for cognitive camera control	QUADAS-AI	Low
[149]	He (2025)	10.1007/s00464-025-11693-6	Laparoscopic low anterior rectal resection – biophysical modeling + AI for quantitative assessment of anastomotic perfusion	QUADAS-AI	Low
**Surgeon perception, preparedness, and attitudes**					
[150]	Acosta (2025)	10.1016/j.cirsp.2024.12.003	National survey among Spanish surgeons – assessment of knowledge, training, and attitudes toward data governance and digital surgery (including robotic practice)	AXIS (cross-sectional survey)	Moderate
[151]	Luense (2023)	10.1007/s00423-023-03134-6	National survey among German surgeons – perceptions and expectations toward AI-based laparoscopic systems	AXIS (cross-sectional survey)	Moderate
[152]	Shafiei (2024)	10.1177/00187208241285513	Simulated robotic and laparoscopic training tasks – evaluation of mental workload using integrated EEG and eye-tracking data analyzed by machine learning	PROBAST	Low-to-Moderate

Supplementary Table S1. Study-level Risk of Bias Assessment.

	First Author (Year)	DOI	AI Algorithm / Model	Dataset Characteristics	Validation Type	Clinical / ex vivo Validation	Median / Representative Performance Values	Key Performance Metrics	Main Limitation
**Object Or Structure Detection**									
[1]	Khalid (2023)	10.1007/s00464-023-10403-4	CNN classifier for inflammation grading	200 laparoscopic cholecystectomy videos (MGH dataset)	Internal	Clinical	Krippendorff's α = 0.71 (vs surgeon α = 0.82)	Krippendorff's α	No external validation, small dataset
[2]	Ward (2022)	10.1007/s00464-022-09009-z	CNN-based Bayesian model	200 laparoscopic cholecystectomy videos labeled by surgeon	Internal	Clinical	α = 0.71 (95% CI 0.65–0.77)	Krippendorff's α	Single-center, limited dataset
[3]	Orimoto (2024)	10.1007/s00464-024-11514-2	YOLOv8 + alarm system	3 600 frames from 89 laparoscopic videos	Internal	Clinical	mAP 95.8%, Precision 89.8%, Recall 91.7%	mAP, Precision, Recall	Single-institution, limited testing
[4]	Kolbinger (2023)	10.1097/JS9.000000000000595	DeepLabv3 & SegFormer	13 195 annotated laparoscopic frames from 32 rectal resections	Internal	Ex vivo	IoU 0.23–0.85 (dep. on structure)	Mean IoU	Experimental dataset, no clinical validation
[5]	Sato (2022)	10.1007/s00595-022-02508-5	Machine-learning segmentation model	1 242 annotated images from 6 patients	Internal (cross-patient)	Clinical	IoU = 0.71 median	IoU	Very small sample, preliminary work
[6]	Igaki (2022)	10.1097/DCR.0000000000002393	Deep-learning segmentation	600 images from 32 colorectal cases	Internal	Clinical	Dice ≈ 0.8 (high accuracy)	Dice coefficient	Feasibility only, small dataset
[7]	Jearanai (2023)	10.1007/s00464-023-10309-1	YOLOv8 object detector	3 600 images (89 lap cases)	Internal	Ex vivo	mAP 95.8%, Precision 89.8%, Recall 91.7%	mAP, Precision, Recall	Small dataset, single institution
[8]	Oh (2024)	10.1038/s41598-024-73434-4	DeepLabV3+ (ResNet50 encoder)	300 frames from 30 PLDH videos	Internal	Clinical	DSC 0.73 (bile duct), 0.43 (anterior wall)	Dice similarity coefficient	Small sample, single surgeon
[9]	Benavides (2024)	10.3390/s24134191	CNN (Hourglass-based model)	3 datasets (ITAP, Atlas Dione, EndoVis)	Internal + External	Ex vivo	Accuracy 92.9%, FPS 27.6	Accuracy, FPS	Limited generalizability, small external test
[10]	Gazis (2022)	10.3390/bioengin	Transformer + 3D CNN (self-supervised)	80 videos (peg transfer	Internal + External	Ex vivo (simula	Accuracy 88–97%	Accuracy	Simulated environment

		eering9120737		& knot tying tasks)	(JIGSAWS)	ted tasks)			, small dataset
[11]	Tomioka (2023)	10.21873/anticanres.16725	Deep learning segmentation model for tubular structure recognition	>350 annotated frames from laparoscopic hepatectomy videos	Internal	Clinical (intraoperative videos)	IoU 0.42; Dice 0.53	IoU, Dice	Small dataset, no external validation, subjective qualitative assessment
[12]	Cui (2021)	10.1155/2021/5578089	YOLOv4 convolutional neural network	3,800 labeled images from 35 laparoscopic inguinal hernia cases	Internal + External (image & video test sets)	Clinical (intraoperative dataset)	TPR 90.61%, TNR 98.67%, PPV 98.57%, ACC 94.61%, F1 94.42%, AP 92.38% (IoU 0.3)	Accuracy, F1-score, AP	Single-center dataset, small sample size, no multicenter validation
[13]	Memida (2023)	10.1109/EMBC40787.2023.10341025	YOLACT++ instance segmentation model	Experimental videos of laparoscopic forceps under varying illumination conditions	Internal	Ex vivo (simulation and controlled lighting)	Highest accuracy achieved in light-shielded environment	Accuracy, FPS	Limited dataset, non-clinical environment, single experimental setup
[14]	Nwoye (2019)	10.1007/s11548-019-01958-6	Weakly supervised CNN + ConvLSTM	Cholec80 dataset, 80 videos annotated with tool presence	Internal	Clinical	+5% detection, +13.9% localization, +12.6% tracking vs baseline	Detection accuracy, IoU, tracking precision	Weak supervision, no external dataset
[15]	Jurosch (2024)	10.1007/s11548-024-03220-0	CNN + temporal model for trocar detection	4 laparoscopic surgeries with dual extra-abdominal cameras	Internal	Clinical	F1 (detection) = 95.1 ± 0.9%, F1 (occupancy) = 89.3 ± 5.3%	F1 score	Small dataset, preliminary feasibility
[16]	Sánchez-Brizuela (2022)	10.3390/s22145180	U-Net, YOLOv3	4,003 annotated frames from laparoscopic simulator	Internal	Ex vivo (simulated laparoscopic setup)	IoU 0.85, >30 FPS (U-Net baseline)	IoU, FPS	Non-clinical data, limited realism
[17]	Lai (2023)	10.1007/s10439-022-03033-9	YOLOv5x6 deep CNN	14 laparoscopic colorectal surgeries	Internal	Clinical	PPV 0.92, Sensitivity 0.83, mAP 0.88, Accuracy 0.90	PPV, Sensitivity, mAP, Accuracy	Single-center, no external validation
[18]	Ehrlich (2022)	10.3390/s22155808	ML classifier on current-sensor signals	Electrosurgical tool and grounding pad sensor data (varied tissues)	Internal	Ex vivo	Accuracy 95.56%	Accuracy	Limited to sensor-based data, no visual AI

[19]	Carstens (2023)	10.1038/s41597-022-01719-2	Dataset (multi-organ segmentation baseline CNNs)	13,195 annotated frames from 32 robot-assisted rectal resections	Internal	Clinical	Pixel-wise segmentation across 11 structures	Dice, IoU	Dataset only, no external validation
[20]	Yin (2023)	10.1016/j.neunet.2023.11.055	LDCNet (Lightweight Dynamic Convolution Network with DDMF module)	78 TaTME surgical videos (Sun Yat-sen Univ., 1920×1080 px, 30 FPS) + Kvasir dataset	Internal (TaTME) + External (Kvasir)	Clinical	Outperformed SOTA on both datasets; real-time inference	Dice, IoU, FPS	Single-center dataset, limited external validation, no public release of TaTME dataset
[21]	Tashiro (2024)	10.1002/jhbp.1422	AI model (Eureka, Anaut Inc.) for loose connective tissue (LCT) segmentation + fusion with ICG fluorescence imaging	60 laparoscopic cholecystectomy training videos (>30,000 LCT annotations)	Internal	Clinical	IoU = 0.56; Dice = 0.60; <0.12 s inference time	IoU, Dice, Inference time	Limited to normal anatomy; reduced performance in inflamed or bleeding cases
[22]	Petracchi (2024)	10.1016/j.gassur.2024.03.018	YOLOv8-based real-time detection software	40 elective laparoscopic cholecystectomy videos (25 gallstones, 15 cholecystitis)	Internal (prospective single-center)	Clinical	100% agreement with 3 blinded expert surgeons; one early false-positive detection	Accuracy, concordance rate	Small sample size, only elective cases, no external validation
[23]	Schnelldorfer (2024)	10.1097/SLA.0000000000006294	CASL (YOLOv5 detector + ensemble classifier)	132 patients; 4,287 lesions (365 biopsied)	Internal	Clinical	AUC-PR 0.69 (detection); AUC-ROC 0.78 (classification)	AUC, Precision, Recall, IoU	Single-surgeon dataset; sin multicentric validation

[24]	Kitaguchi (2023)	10.1093/bjs/znad249	UreterNet (FPN+EfficientNetB7) & NerveNet (OCRNet/HRNet)	252 laparoscopic colorectal videos (10 711 ureter; 14 577 nerve images) with prospective intraoperative testing	Internal + Prospective	Clinical	DSC ureter = 0.722; hypogastric nerves = 0.579; aortic plexus = 0.628; intra-op recognition ≥ 89–95 %	Dice, Recall, Precision	Single-centre; no external or multicentre validation
[25]	Chen (2025)	10.1093/bjsopen/zrae158	DeepLabv3+ (ResNet-50) for perigastric vases	2 460 annotated images from 116 gastrectomy videos; 15 extra videos for dynamic validation	Internal + External	Clinical	Artery IoU 0.86 / F1 0.93; Vein IoU 0.80 / F1 0.89	IoU, F1, Recall, Precision	Lower performance under smoke/bleeding; retrospective study
[26]	Han (2025)	10.1097/DCR.0000000000003547	DeepLabv3+ (ResNet50_Vd)	1 780 annotated frames from 3D laparoscopic TME procedures	Internal	Clinical	mIoU 0.75; Precision 0.75; Recall 0.66; F1 0.70	IoU, Precision, Recall, F1	Single-centre; limited dataset; no external validation
[27]	Yoshihiko (2023)	10.1002/jhbp.1388	DeepLabv3 (color-coded vessel segmentation)	350 frames from 8 laparoscopic liver resections (1920×1080 px)	Internal	Clinical (preliminar)	IoU 0.42; Dice 0.53; inference <0.12 s	IoU, Dice, speed	Small dataset; early feasibility
[28]	Frey (2025)	10.1007/s11701-025-02284-7	YOLOv8 (detection, classification, segmentation of surgical instruments)	>7,400 frames and 17,175 annotations from 4 datasets (public + private; robotic and laparoscopic tools)	Internal + External (multi-source)	Clinical	mAP (binary) 0.77; mAP (multi-instrument) 0.72; Dice 0.91; IoU 0.86; inference 1.12 ms/frame	mAP, Dice, IoU, inference time	Overrepresentation of robotic tools; no prospective intraoperative testing

[29]	ElMoaq et (2025)	10.3390/s25103017	BEiT (masked image modeling ViT) — classification + weakly supervised localization	Cholec80 (tool presence); Cholec80-Boxes (5 videos, boxes)	Internal (benchmark)	Ex vivo (public laparoscopic dataset)	SOTA in classification/localization	Cholec80	No clinical validation
[30]	Korndorffer (2020)	10.1097/SLA.0000000000004207	3D-CNN + Temporal ConvNet + LSTM (CVS/eventos)	1,051 laparoscopic cholecystectomy videos (31 surgeons, 2 centers)	Internal multicenter	Clinical	AI–surgeon agreement >75% for CVS; 99% event concordance	Accuracy, Agreement rate, Event frequency, AUC	Retrospective; heterogeneous video quality; reduced accuracy in complex cases
[31]	Ryu (2023)	10.1007/s11605-023-05819-1	Eureka AI navigation (Anaut Inc.) for nerve highlighting	10 laparoscopic left colorectal surgeries (prospective 2022)	Internal prospective	Clinical	Correct recognition of 100% lumbar splanchnic and 87% hypogastric nerves (junior trainees)	Recognition rate	Small sample; training-focused; no quantitative accuracy metrics
[32]	Park (2020)	10.3748/wjg.v26.i44.6945	AIRAM (AI-based real-time ICG perfusion analysis using SOM network)	65 laparoscopic colorectal cancer surgeries (50 training + 15 test; 10,000 ICG curves)	Internal (train/test split)	Clinical	F1 ↑ 31% vs T1/2max; tiempo processing ≈48 s	F1, ROC, Accuracy	Single-center; retrospective; limited to ICG perfusion context
[33]	Ryu (2024)	10.1007/s00464-023-10524-w	Deep learning segmentation model (SMV, ICA, ICV)	2,624 images from 104 laparoscopic right hemicolectomy videos	Internal (fivefold cross-validation)	Clinical	SMV Dice >0.75; ICA/ICV Dice 0.53–0.57	Dice, Recall, Precision	Single-center; small dataset; lower accuracy for small vessels

[34]	Zygomalas (2024)	10.1177/15533506241226502	YOLOv8 for landmark and instrument recognition	25 TAPP videos (20 institutional + 5 online); 1,095 images, 2,716 annotations	Internal	Clinical	F1 = 0.82; mAP50 = 0.873	F1, Precision, Recall, mAP	Small dataset; limited classes; retrospective design
[35]	Mita (2025)	10.1007/s10029-024-03223-5	EUREKA (Anaut) — conectivo/vasos/nervios	10 TAPP surgeries; 3,000 annotated images from 150 laparoscopic/robotic cases	Internal	Clinical	IoU: conectivo 0.33, nervio 0.24, vaso 0.50, microvasos 0.30; Dice 0.50–0.66	IoU, Dice	Early feasibility; qualitative validation only; limited generalization
[36]	Tomioka (2024)	10.21873/anticanres.16725	Deep learning segmentation model for hepatic vein and Glissonean pedicle recognition	>350 annotated frames from laparoscopic hepatectomy videos	Internal	Clinical (intraoperative video)	IoU 0.42; Dice 0.53; surgeons' sensitivity 4.24±0.89; misrecognition 0.12±0.33	IoU, Dice, qualitative surgeon scores	Small dataset; qualitative assessment; no external validation
[37]	Horita (2024)	10.1007/s00464-024-10874-z	YOLOv7_6w detector for real-time active-bleeding vs blood-pooling classification	27 colectomy videos (train 21 / val 3 / test 3); 34 117 frames, 254 bleeding events	Internal split	Clinical	AP.50 (active bleeding) = 0.574; 48.5 FPS; surgeon sensitivity 4.92 / 5; over-detection 4.62 / 5	AP.50, FPS, surgeon ratings	Small test set; no external validation
[38]	Kinoshita (2024)	10.1007/s00464-024-10939-z	U-Net-based model for autonomic nerve segmentation and recognition	60 test frames from laparoscopic + robotic rectal cases; surgeon questionnaire	Internal	Clinical	Mean Dice 0.442 (range 0.0465–0.639); IoU 0.292; usefulness score 3.38 / 5	Dice, IoU, subjective usefulness	Limited frames; single centre; no external testing

[39]	Takeuchi (2023)	10.1007/s00464-023-09934-7	Deep neural object detector for CVMPO landmark recognition	160 TAPP videos (train 130, test 30)	Internal (train/test)	Clinical	Single-image mAP 51.2 %; video Accuracy 77.1 %; F1 75.4 %; inter-rater agreement 88.3 %	mAP, Accuracy, F1, Agreement	Single centre; limited landmark scope; no external validation
[40]	Une (2024)	10.1007/s00464-023-10637-2	Feature Pyramid Network + EfficientNetV2-L for hepatic vein and Glissonean pedicle segmentation	2 421 frames from 48 LLR videos (5-fold cross-validation)	Internal	Clinical	2-class Dice 0.789; 3-class Dice 0.631 (hepatic vein), 0.482 (Glissonean); ≈ 0.095 s per frame	Dice, inference speed	Retrospective; single centre; limited dataset
[41]	Kojima (2023)	10.1097/JS9.000000000000317	DeepLabV3+ (Xception backbone) for autonomic nerve segmentation (HGN, SHP)	12 978 HGN and 5 198 SHP frames from 245 + 44 colorectal videos	Internal (5-fold)	Clinical	Dice 0.56 ± 0.03 (HGN); 0.49 ± 0.07 (SHP); early recognition ≈ 50 % of cases	Dice, Precision, Recall	Single centre; limited sample; no external validation
[42]	Nakanuma (2022)	10.1007/s00464-022-09678-w	Intraop. landmark detector (SES); rubric + Dice vs external evaluators	10 LC cases; prospective feasibility	Internal	Clinical (in-OR)	Rubric score for LM-CBD 4.2 ± 0.8/5; AI–evaluator Dice comparable to inter-evaluator Dice	Rubric, Dice	Small n, single-centre; no external test
[43]	Loukas (2022)	10.1002/rcs.2445	Two-stage Multiple-Instance CNN (autoencoder + MIL)	68 LC videos → 234 images; vascularity labels	Internal	Clinical (retrospective images)	Accuracy 92.6–93.2%; F1 93.5–93.9% (image/video level)	Acc., F1	Not real-time; single dataset; no external validation

[44]	Endo (2023)	10.1007/s00464-023-10224-5	Real-time AI landmarks (EHBD, CD, RS, S4); before/after user study (beginners+experts)	160 images; 8 participants	Internal (pre/post with AI overlay)	Clinical videos	26.9% annotation changes; ~70% changes toward safer interpretations; ↑ confidence in RS/S4	% changes, user-reported benefit	Non-randomized user study; subjective endpoints; no external test
[45]	Fried (2024)	10.1097/SLA.0000000000006377	Proprietary surgical-intelligence CV platform	279 LC videos (46 surgeons, 2 hospitals)	Prospective observational	Clinical real-world	CVS adoption 39.2→69.2% ($p<0.001$); time 54→44 min ($p=0.033$); adverse events ↓ ($p=0.011$)	Adoption, time, events	Proprietary; no external validation; follow-up limited
[46]	Mascagni (2022)	10.1097/SLA.0000000000004351	DeepCVS (segmentation + multi-label classification)	2,854 images / 201 LC videos (402 segmented)	5-fold CV	Clinical videos	IoU 66.6%; AP 71.9%; balanced acc. 71.4%	IoU, AP, Bal. Acc.	Static frames; single centre; no live deployment
[47]	Fujinaga (2023)	10.1007/s00464-023-10097-8	AI-based intraoperative assistance system for landmark and CVS identification	Clinical LC videos	Internal	Clinical (retrospective video analysis)	-	Qualitative/diagnostic support	Single-centre; no external validation; descriptive evaluation
[48]	Kawamura (2023)	10.1007/s00464-023-10328-y	EfficientNet-B5 with Sharpness-Aware Minimization for CVS scoring	72 LC videos (71 379 frames; 20 for evaluation)	Internal (cross-validation)	Clinical	Precision 0.971; Recall 0.737; F1 0.832; Specificity 0.966; Accuracy 0.834; 6 fps	Precision, recall, F1, specificity, accuracy	Single-centre; no acute cholecystitis; no external validation

[49]	Tokuyasu (2021)	10.1007/s00464-020-07548-x	YOLOv3 object-detection model for intraoperative landmark recognition	99 LC videos (2339 images; 76 train / 23 test; landmarks: CD, CBD, S4, RS)	Internal	Clinical (prototype verification)	AP = CBD 0.320, CD 0.074, S4 0.314, RS 0.101; 37.2 fps	AP, FPS, surgeon agreement	Small dataset; limited inflammation cases; flicker effect; no external validation
[50]	Zhang (2024)	10.3760/cma.j.cn441530-20240125-00041	YOLOv8m instance-segmentation network for organ and instrument recognition	8 videos from 4 tertiary hospitals (3 PLA General, 2 Liaoning Cancer, 2 Jiangsu Liyang, 1 Fudan); 3 369 frames (1 920 × 1 080 px), 23 categories	Internal (9:1 train/validation split)	Clinical (multicenter surgical videos)	Train mAP = 0.99; Validation mAP = 0.82; AP (ultrasonic knife 0.96, needle holder 0.94, forceps 0.91, gallbladder 0.91, gauze 0.91, stapler 0.91); stable loss curve	mAP, AP, precision, recall	Small sample (8 videos); no external test set; limited intra-patient variability
**Skill Assessment And Training**									
[51]	Ortenzi (2023)	10.1007/s00464-023-10375-5	Video Transformer Network + LSTM for automatic workflow step recognition in TEP hernia repair	619 full-length TEP videos (371 train / 93 val / 155 test)	Internal split	Clinical (intraoperative videos)	Overall accuracy 88.8%; per-step range 72.2–94.3%	Accuracy (overall & per-step)	Single dataset; no external validation; procedure-specific
[52]	Wu (2024)	10.1097/JS9.0000000000001798	SurgSmart AI coaching platform (phase recognition + CVS scoring)	22 surgeons, 10 hospitals, 90 LC videos (RCT)	Prospective randomized controlled	Clinical	LCRF score ↑ from 31→40 ($p=0.008$); CVS completion ↑ 11%→78% ($p=0.021$)	LCRF, CVS completion, procedural metrics	Small RCT; single-country cohort; short follow-up

[53]	Belmar (2023)	10.1007/s00464-022-09576-1	U-Net + YOLOv4 for object detection and segmentation in dry-lab simulation	400 bean-drop + 480 peg-transfer videos	Internal	Simulation	Expert agreement 79.7% (κ=0.59, BD) and 93.0% (κ=0.86, PT)	Cohen's κ, accuracy	Limited exercises; no external validation
[54]	Halperin (2024)	10.1007/s11548-023-02963-6	YOLOv5 + ResNet-18 + U-Net for motion tracking and suture precision	29 intracorporeal suture videos (10 participants: 5 residents, 5 surgeons)	Cross-validation	Simulation (FLS trainer)	mAP@0.5 = 0.995; Dice = 0.72; RMSE = 0.0888; Accuracy = 86%	mAP, Dice, RMSE, accuracy	Small dataset; single-center; preliminary feasibility
[55]	Chen (2023)	10.1007/s11701-023-01713-9	Vision-based trajectory monitoring system using computer-vision tracking of handle motion paths	40 participants (20 experts, 20 novices); dry-lab suturing videos recorded with robot-mounted camera array	Internal (comparative analysis)	Simulation / dry-lab	Experts vs novices: center distance ↓ ($p < 0.001$), proximal distance ↑ ($p < 0.001$), path/volume ratio ↓ ($p < 0.001$); accuracy ↑ in experts	Trajectory metrics (center & proximal distance, path ratio, volume ratio, accuracy)	Small sample (40); simulation only; no AI model generalization tested
[56]	Ismail Fawaz (2019)	10.1007/s11548-019-02039-4	Fully Convolutional Network (FCN) on kinematic time series; CAM for interpretability	JIGSAWS robotic tasks; skill classification + OSATS regression	Internal CV on public dataset	Simulation / training	Reached state-of-the-art on JIGSAWS (classification & regression); interpretable CAM highlights motion segments	Accuracy (skill), OSATS regression, interpretability	Public benchmark only; no clinical videos; overfitting risk on small dataset
[57]	Nguyen (2019)	10.1016/j.cmpb.2019.05.008	CNN-LSTM with SENet block; minimal preprocessing of motion signals	IMU platform (open-surgery core tasks) + JIGSAWS evaluation; 15 participants (4 experts, 4 intermediates, 7 novices)	Internal	Simulation / training	Accuracy 98.2% (own IMU data); on JIGSAWS: 98.4% (suturing), 98.4% (needle-passing), 94.7% (knot-tying)	Accuracy per task	Small cohorts; sensor platform setting; no clinical validation

[58]	Wang (2018)	10.1109/EMBC.2018.8512575	SATR-DL: parallel CNN + GRU; multi-output (skill & task)	JIGSAWS motion sequences; 4-s intervals; ensemble to trial level	Internal	Simulation / training	Trial-level accuracy: 0.960 (skill), 1.000 (task)	Skill & task accuracy (interval & trial)	Conference study; public dataset only; no clinical videos
[59]	Funke (2019)	10.1007/s11548-019-01995-1	3D ConvNet (inflated) + Temporal Segment Network; video-only	JIGSAWS videos (robotic bench-top tasks)	Internal	Simulation / training	Skill classification accuracy 95.1–100.0% across tasks	Accuracy (video-only)	Video benchmark only; needs larger annotated video sets
[60]	Partridge (2014)	10.1089/lap.2014.0015	Color thresholding CV (InsTrac) to track instrument tips; real-time metrics	23 participants: 10 novices vs 13 trainees; eoSim take-home box	Internal (construct validity)	Simulation	Significant differences novices vs trainees: time 9:53 vs 2:33 ($p=0.01$); distance 11.38 m vs 3.29 m ($p=0.01$); smoothness 0.06 vs 0.15 mm/s^3 ($p<0.01$); handedness difference 2.43 m vs 0.55 m ($p=0.03$)	Time, distance, smoothness, handedness	Small sample; 2D tracking; limited tasks; no external validation
[61]	Derathé (2025)	10.1038/s41597-025-04588-7	Dataset (no model): multimodal annotations for sleeve gastrectomy (LapEx)	30 videos; fundus dissection step; procedural activities (quadruplet), skill “quality of exposure” (1–3) with 735 assessments, full-scene segmentation with 11 classes on 735 images	—	Clinical (video dataset)	Not applicable (dataset descriptor; provides baselines/variability, not model performance)	Dataset scope & annotation variability	Dataset only; monocentric acquisition; class imbalance; requires DTA for access

[62]	Bogar (2024)	10.1038/s41598-024-67435-6	AI evaluator for peg-transfer on a low-fidelity VR simulator (objective scoring)	60 students, RCT VR vs box trainer; 240 peg-transfer tasks	Prospective randomized	Simulation	AI score correlated with expert ratings; significant training effect in VR arm (per paper)	Agreement with experts; learning effect	Low-fidelity task; student cohort; no surgical videos.
[63]	Matsumoto (2024)	10.1038/s41598-024-63388-y	Instrument-tip identification (AI) → kinematic fluctuation analysis (LDG)	18 LDG videos (3 experts, 3 novices; ~1.25M frames)	Internal	Clinical videos	Dice 0.837, TPR 0.873, FPR 0.0041 for Harmonic detection; β-index cutoff 1.4 → sensitivity/specificity 77.8%	Dice, TPR, FPR, β-index	Small sample; single-centre; no external set.
[64]	Gillani (2024)	10.1016/j.jss.2024.07.103	ML-derived Objective Performance Indicators (OPIs) from Da Vinci logs for skill stratification	Robotic right colectomies; experts vs intermediates vs novices (Emory cohort)	Prospective observational	Clinical	OPIs differed significantly across skill levels (e.g., movement/arm-kinematics metrics; p-values reported in paper)	OPI differences, p-values	Single centre; procedure-specific; industry co-author.
[65]	Yang (2023)	10.1007/s00464-022-09781-y	Computer vision on robotic colorectal videos; tool tracking/segmentation → correlate with GEARS	92 clips of peritoneal closure; 6 surgeons GEARS scoring	Internal	Clinical	Reported tool mAP (segmentation/detection) and significant correlations (e.g., efficiency vs movement) with GEARS	mAP, r (GEARS domains)	Moderate N; partial manual labels; single institution.
[66]	Caballero (2024)	10.1007/s11548-024-03218-8	MLR / SVM / MLP on EDA + kinematics + vitals to predict surgical stress	26 robotic sessions, 11 surgeons; ankle-worn EDA + motion	Train/val/test split	Experimental / mixed	Best: MLR $R^2 \approx 0.83$ (lowest error) vs other models	R^2, error (MAE)	Small sample; wearable placement; no real-time OR deployment.

[67]	Yanik (2024)	10.1007/s44186-023-00223-4	Self-supervised contrastive feature extractor + 1D ResNet for skill classification and score prediction	23 trainees, > 1600 FLS suturing trials over 2 weeks (video only)	Internal cross-validation	Simulation (FLS box trainer)	Accuracy = 0.878 ± 0.002 (Pass/Fail); Spearman r = 0.746 ± 0.002 (performance scores)	Accuracy, Spearman correlation	Simulation-only data; short-term training period; no external validation
[68]	Nakajima (2024)	10.1007/s00464-024-11208-9	CNN-based phase recognition (EfficientNet architecture) for workflow and skill correlation	1,272 laparoscopic sigmoidectomy videos (2016–2019, Japan)	Internal split	Clinical (video dataset)	Moderate correlation between predicted phase features and ESSQS skill levels (r ≈ 0.6)	Precision, recall, F1, correlation r	Single-country dataset; no external validation; skill inferred indirectly from phase model
[69]	Yamazaki (2022)	10.1007/s11605-021-05161-4	YOLOv3 for automatic detection of 14 surgical instruments and usage duration	133 videos (100 infrapyloric + 33 suprapancreatic lymphadenectomy)	Internal	Clinical (video dataset)	Agreement between AI and manual usage time detection > 90 %; distinct usage patterns between qualified and non-qualified surgeons	Detection accuracy, usage time correlation	Retrospective; potential case-mix bias; no external validation
[70]	Allen (2009)	10.1007/s00464-009-0556-6	Support Vector Machine using motion metrics for skill classification	696 trials by 30 subjects (4 experts, 26 novices) on 3 laparoscopic tasks	Internal	Simulation	AUC 0.968 / 0.952 / 0.970; accuracy 93.7 % / 91.3 % / 90.0 % for peg transfer, rope pass, cap needle	AUC, accuracy	Simulator only; small expert sample; no clinical testing
[71]	Fukuta (2025)	10.1007/s11548-024-03253-5	DeepLabCut for pose estimation of forceps trajectories (top and side views)	Training simulator recordings; subset (5 %) for manual validation	Internal	Simulation	High tracking accuracy for hinge points; unstable tip tracking during rotation; qualitative evaluation only	Tracking error (% frames accurate)	Feasibility study; small dataset; no quantitative skill correlation

[72]	Moglia (2022)	10.1007/s00464-021-08999-6	Ensemble Deep Neural Networks (DNNs) for prediction of robotic surgical proficiency acquisition	176 medical students performed five virtual da Vinci tasks on the Mimic dV-Trainer simulator	Internal comparison (train/validation split)	Simulation environment	Best model accuracy ≈ 0.87 for classifying trainees reaching proficiency; clear learning-curve trend captured	Accuracy (proficiency classification), learning-curve fit	Limited to simulator data; no clinical validation; moderate sample imbalance
[73]	Ju (2025)	10.1007/s00464-025-11730-4	3D ResNet-18 + LSTM for gesture recognition and automated skill classification	80 annotated peg-transfer videos (CLSTA standard dataset); split into train/val/test sets	Cross-validation (5-fold)	Simulation / dry-lab	Overall accuracy 85.8 %, macro F1 85 %, precision 84 %, recall 86 %	Accuracy, F1, precision, recall	Limited sample size; single task only; no external validation
[74]	Cruz (2025)	10.1007/s44186-025-00355-9	YOLOv4 object detection + time measurement for simulation task evaluation	7,673 videos (laparoscopic simulation platform); 1,100 frames for detection; 880 videos for timing	Internal validation	Simulation / training	Precision 0.94, Recall 0.89, F1-score 0.81–0.97 (object detection); Pearson r = 0.96 (timing correlation with expert teachers)	Precision, recall, F1, Pearson correlation	No clinical validation; single simulation platform; retrospective performance evaluation
[75]	Chen (2024)	10.1097/JS9.000000000000975	Classic ML (LR, SVM, RF, GBDT, AdaBoost) on 63 Surgesture features (counts/durations/ratios, shift freq.)	75 LC videos from 33 surgeons / 5 hospitals; phases MHT & DGB annotated with 14 Surgestures	5-fold CV (internal)	Clinical videos	AUC = 0.866 (binary: competent vs incompetent); higher cholecystic vascular injury in incompetent 30.8% vs 6.1%	AUC; peri-op correlation (events vs skill)	Retrospective; only two phases; no external validation.
[76]	Erlich-Feingold (2025)	10.1007/s00464-025-11715-3	Vision Transformer (ViT) video classifier; separate Xception single-image model	135 videos, 46 participants (16 experts, 30 novices); FLS precision cutting task	5-fold CV (internal)	Simulation	Accuracy = 0.867 (video); AUC = 0.95, Sens 0.87, Spec 0.87; single-image accuracy 0.57	Accuracy, AUC, Sens/Spec	Single centre; simulation only; modest image-only results.

[77]	Power (2025)	10.1038/s41598-025-96336-5	3DCNN, weakly-supervised, on new LSPD dataset	106 videos, 40 participants (novice/trainee/expert); 3 skills (bands/stack/tower); augmented to 2,244 vids	Internal	Simulation	F1 = 0.91, AUC = 0.92 for multi-class skill level	F1, AUC	Small base dataset; simulated tasks; no clinical validation.
[78]	Alonso-Silverio (2018)	10.1177/1553350618777045	ANN on box-trainer traces; CV + AR for tracking & tasks	20 participants (4 experts, 6 residents, 10 students); 2 tasks (transferring, pattern cutting) over 5 days	Hold-out / k-fold / LOO	Simulation	Accuracy = 90.98%, AUC = 0.93 (pattern-cutting classifier)	Accuracy, AUC	Small cohort; low-cost hardware; no clinical data.
[79]	Belmar (2022)	10.1007/s00464-022-09576-1	U-Net + YOLO v4 for segmentation & object detection of graspers and task elements	400 Bean Drop + 480 Peg Transfer videos for training; 64 BD + 43 PT for testing (from 14 institutions, Latin America)	Internal (test subset)	Simulation (training platform)	Agreement AI vs experts: 93.0 % (κ = 0.86) for PT; 79.7 % (κ = 0.59) for BD	Cohen's κ; agreement %	Limited sample per exercise; restricted to basic tasks; no external validation; technical errors in object fall labeling
[80]	Pan (2010)	10.1002/rcs.399	Custom computer-vision metrics and motion analysis system for laparoscopic rectal training	20 trainees performing simulated LAR tasks on phantom model	Internal	Simulation	Strong correlation between AI metrics and expert scores (r = 0.82); task-time vs accuracy significant ($p < 0.05$)	Correlation r, p-values	Small cohort; no external validation or real patients
[81]	Ershad (2019)	10.1007/s11548-019-01920-6	Unsupervised learning on joint-position data from da Vinci system; Random Forest for style classification	Kinematic data from 16 surgeons (4 experts, 12 novices); ≈ 400 robotic tasks	5-fold cross-validation	Robotic simulation / in vivo videos	Mean classification accuracy = 0.91; F1 = 0.88	Accuracy, F1	Small sample; limited generalizability; no external validation

[82]	Kowalewski (2020)	10.1007/s00464-019-06667-4	Narrative synthesis of AI skill-assessment tools and datasets	–	–	No	Not applicable (review article)	–	Descriptive only; no quantitative metrics
[83]	St John (2024)	10.1007/s00464-024-11068-3	Deep CNN integrated into mobile game for safe LC dissection feedback	> 12,000 annotated frames of Calot's triangle; internal pilot with 20 trainees	Internal	Simulation	Overall accuracy = 0.88, AUC = 0.93 for safe/unsafe dissection classification	Accuracy, AUC	Mobile simulator only; limited real-world validation
[84]	Yen (2025)	10.1007/s00464-025-11663-y	Random Forest + VideoMAE models for competency prediction and action recognition	80 Cholec80 videos (LC, Calot's Triangle Dissection phase); 66 train / 14 test	Internal	Clinical videos	Random Forest AUC = 0.96 (acc 93%); VideoMAE acc = 89.1%, recall 0.97 for dissecting	Accuracy, AUC, F1	Small dataset; single public source; no external validation
[85]	Nakajima (2025)	10.1007/s00423-025-03641-8	Deep CNN recognition of tissue dissection by monopolar device	766 LC videos (multi-centre, Japan); 8 tool detection models + dissection on/off classifier	Internal	Clinical (intra-op videos)	Recognition accuracy = 0.91; r = 0.542 (95% CI 0.288–0.724, p < 0.001); significant differences across skill groups (p = 0.0081)	Accuracy, correlation, p values	Moderate correlation with manual scores; no external validation
[86]	Igaki (2023)	10.1001/jamasurg.2023.1131	CNN (Xception) deep-learning model recognizing standardized surgical fields and outputting an AI Confidence Score (AICS)	650 intra-op videos of laparoscopic sigmoid colon resection (ESSQS dataset, Japan Society for Endoscopic Surgery); 60 used for	Hold-out validation	Clinical videos	Spearman r = 0.81 (p < 0.001) between AICS and ESSQS score; AUC = 0.93 (low-score) and 0.94 (high-score) screening; model accuracy for	r, AUC, Sensitivity/Specificity	Exploratory; small training set (60 cases); no prospective validation; does not provide feedback to surgeons

				training, 60 for validation			step classification = 78.2 %		
[87]	Smith (2021)	10.1007/s11701-021-01284-7	Deep Neural Network (Google AutoML Video Intelligence) for video-based classification	254 simulation videos (two exercises, scored by expert instructors); converted to 2,227 clips (10 s) for model training	Internal (AutoML split)	Simulation	Accuracy = 83.1% (Ring & Rail), 80.8% (Suture Sponge) for 3-level classification (expert/intermediate/novice)	Accuracy	Limited dataset; short clips (10 s) reduce context; no external or clinical validation
**Workflow Recognition And Intraoperative Guidance**									
[88]	Loukas (2024)	10.1002/rcs.2632	TransLocal: saliency-guided CNN-LSTM features → Transformer (local attention) for RSD	Cholec80 LC videos	Internal	Clinical (video)	MAE 7.1 min overall; long vs short: 10.6 vs 4.4 min; at T–30 min: MAE 6.2 min (all), 7.2/5.5 min (long/short)	MAE (minutes)	Single dataset; no external test set.
[89]	Wagner (2023)	10.1016/j.media.2023.102770	Comparative benchmark (12 teams) on HeiChole	33 LC videos, 3 centers; labels: 7 phases, 4 actions, 21 instruments, 5 skill dims	Held-out test (challenge)	Clinical (video)	Phase F1: 23.9–67.7%; Instrument presence F1: 38.5–63.8%; Action F1: 21.8–23.3%; Skill MAE: 0.78	F1 (phase/action/instrument), MAE (skill)	Small multicenter set; challenge heterogeneity.
[90]	Zhang (2023)	10.1007/s11548-022-02811-z	ASTCFormer: R(2+1)D clip encoder → ASFormer + TCN fusion (full-video)	207 (robotic + lap) LC videos (Cholec207); also Cholec80	Internal split	Clinical (video)	+2.7% relative improvement in segmental F1 vs ASFormer; state-of-the-art on Cholec80 (per paper)	Segmental F1	Single-source Cholec207; company affiliation; no external validation.

[91]	Park (2023)	10.1016/j.compbiomed.2023.107453	Multimodal fusion: CNN visual features + Visual Kinematics Index (VKI) from semantic masks	VR PETRAW + private distal gastrectomy dataset	Internal	Clinical + Simulator	Improved phase recognition vs CNN-only and comparable to transformer-based fusion on limited data (exact metrics NR)	Accuracy/F1 (phase)	Private clinical data
[92]	Twinanda (2018)	10.1109/TMI.2018.2878055	RSDNet: CNN (progress) + LSTM (no manual annotations) for RSD	120 LC + 170 gastric bypass videos	Internal	Clinical (video)	Outperformed non-DL baselines; generalizable to bypass	MAE (minutes)	Numbers per cohort.
[93]	Zang (2023)	10.3390/bioengineering10060654	Baselines: Video Swin Transformer, Perceiver IO, others	209 RALIHR videos; 8 surgeons (robotic-assisted lap inguinal hernia)	Internal validation	Clinical (video)	Val. accuracy ~0.85 (Video Swin), ~0.84 (Perceiver IO)	Accuracy (phase)	Single health-system; phase defs tailored; limited external generalization.
[94]	Cartucho (2024)	10.1016/j.media.2023.102985	Unsupervised ARFlow-based CNN tracker (top method, EndoVis 2022 challenge)	157 stereo laparoscopic videos (20 clinical cases) for soft-tissue tracking	Internal (held-out test set)	Clinical videos	Best EAO = 0.617 (top submission); baseline CSRT = 0.563	EAO (Expected Average Overlap)	Benchmark study; no external validation or clinical outcomes reported
[95]	Zheng (2022)	10.1007/s11548-022-02568-5	LSTM classifier on instrument kinematics for stress state recognition	Simulation-based training dataset (frames 8–16 s windows)	Leave-one-user-out (LOUO)	Ex vivo simulation	Accuracy = 74.96 % (normal vs stressed, 8 s); 68.18 % (within-trial segments, 16 s)	Accuracy	Small cohort; no clinical validation; stress defined by surrogate task parameters

[96]	Zhai (2024)	10.1007/s11548-023-03027-5	CNN + Temporal Relational Transformer for phase recognition	100 laparoscopic gastrectomy videos (8 phases annotated)	Internal split (train/val/test)	Clinical videos	Accuracy = 90.13 %; F1 = 87.0 %; Precision = 87.3 %; Recall = 87.0 %	Accuracy, F1	Single-center dataset; no external validation; phase imbalance possible
[97]	Takeuchi (2022)	10.1007/s10029-022-02621-x	TeCNO + HMM for multi-phase recognition in TAPP	Intraoperative TAPP videos	Internal split	Clinical videos	NR	NR	Single-center retrospective; no external validation
[98]	Hashimoto (2019)	10.1097/SLA.0000000000003460	Video motion features + classifier for step recognition in sleeve gastrectomy	Retrospective intraoperative videos	Internal	Clinical videos	NR	NR	Internal validation only
[99]	You (2024)	10.1007/s00464-024-10916-6	Transformer-based video phase recognition model for LPD	LPD clinical videos	Internal	Clinical videos	NR	NR	Private dataset; no external validation
[100]	Takeuchi (2023)	10.1007/s00464-023-09924-9	CNN/transformer workflow model for robotic distal gastrectomy	Multicenter RDG dataset	Internal	Clinical videos	NR	NR	Internal-only evaluation

[101]	Zheng (2023)	10.1002/rcs.2449	U-Net / PSPNet / LinkNet / DeepLabv3+ for instrument segmentation; OR (Occupation Rate) central-field index; PaddleSeg deployment	1,888 labeled images (from 80/100 laparoscopic videos) for training/val.; extra 20 videos for OR threshold; clinical pilot with 12 partial nephrectomies (expert vs non-expert; with/without LSQS)	Internal	Clinical videos	PSPNet: Precision 0.9135, F1 0.9058, mIoU 0.8280; PaddleSeg best round: mIoU 0.8892, Precision 0.9621	Precision, Recall, F1, mIoU; χ^2 for OR groups	Single-center; mixed specialties; limited clinical pilot for effect on control; no external validation.
[102]	Dayan (2024)	10.1007/s11695-023-07043-x	Commercial CV platform (Theator) – automated steps, safety milestones, events	49 SG videos (Dec 2020–Aug 2023); surgeon vs AI comparison; step durations	Internal (expert comparison)	Clinical videos	Agreement (AI vs surgeon): Bougie 100%, Pylorus ≥2 cm 100%, Parallel to lesser curvature 98%, Fundus mobilization 100%; Esophagus ≥1 cm: true 100% / false 13.6% (κ=0.20, p=0.006)	Agreement %, κ; step time distributions	Single-center; platform black-box; one criterion with low κ; no outcomes linkage.
[103]	Kitaguchi (2020)	10.1016/j.ijsu.2020.05.015	CNN (Xception) for phase/action; U-Net for tool segmentation (LapSig300)	300 LCRS videos from 19 centers; 82.6M frames (phase/action); 4,243 images (tools)	Hold-out (80/20, by video)	Clinical videos	Phase accuracy 81.0%, Action accuracy 83.2%; Tool mIoU 51.2% (5 tools)	Accuracy (phase/action), mIoU (tools)	Class imbalance; tool set limited; no external dataset beyond hold-out.
[104]	Yoshida (2024)	10.1007/s00423-024-03411-y	CNN (Xception) step classification for LDG; five step-grouping patterns explored	40 LDG videos (train 30 / val 10); >1,000,000 labelled frames	Hold-out	Clinical videos	Overall accuracy 0.89; per-step: Precision 0.88, Recall 0.87, F1 0.88; Grouped Pattern-4 accuracy 0.952	Accuracy; per-step Precision/Recall/F1	Single-institution; modest N; tailored step schema.

[105]	Fer (2023)	10.1007/s00464-023-09870-6	Fully convolutional network with R(2+1)D feature extractor for phase recognition in RYGB	545 laparoscopic RYGB videos from 17 surgeons (EU/US); 390 train / 95 val / 60 test	Internal split	Clinical videos	F1 > 0.90 in 7/12 steps; F1 > 0.80 in 11/12; best ≈ 0.956 (Jejunostomy), worst ≈ 0.779 (Biliary loop measurement); AI comparable to manual ($p < 0.0001$)	Step-wise F1, Accuracy, ANOVA	No external validation; inter-rater variability; possible industry bias
[106]	Liu (2023)	10.1097/JS9.000000000000559	Hybrid CNN + GNN for multilevel workflow recognition (steps, tasks, activities)	45 robotic LLS cases → 4.38 M frames (2.42 M effective)	Train/test split by surgeon (34/11 cases)	Clinical videos	Accuracy = 0.82/0.80/0.79 (steps/tasks/activities); after idle-frame filtering → 0.96/0.88/0.82	Accuracy, Precision, F1	Single-center; computationally intensive; no external validation
[107]	Khojah (2025)	10.1007/s00464-025-11694-5	YOLOv8x-seg / YOLO11x-seg for ureter segmentation	1 237 annotated images (86 videos) + external DSAD subset	Five-fold cross-validation	Clinical + external videos	mAP50 = 0.92, mAP50-95 = 0.53, Precision = 0.94, Recall = 0.88, Dice = 0.90 (peak 0.95)	mAP, Precision, Recall, Dice	Single institution; limited diversity; short sequences
[108]	Lavanchy (2024)	10.1007/s11548-024-03166-3	MTMS-TCN (ResNet-50 features + multi-stage TCN); benchmarks vs CNN, LSTM, MT-LSTM, TeCNO	MultiBypass 140: 140 LRYGB (2 centres: Bern 70, Strasbourg 70); 12 phases, 46 steps; splits per centre and total	Interna (multiples scenarios 1–7)	Clinical videos	Fase – Bern: Acc 85.30%, F1 62.40%; Stras: Acc 90.23%, F1 79.87%; Multi: Acc 87.91%, F1 71.28%.	Accuracy, Precision, Recall, F1 (phase and step)	Clear drop in multicenter crossed validation, no additional external validation.
[109]	Komatsu (2024)	10.1007/s10120-023-01450-w	EfficientNet-B7 image at 1 fps for 9 phases; skill via frames per phase + AI confidence score (AICS)	LDG multicentric: 256 cases (20 hospitals) for training/val.; ESSQS 180 cases for skill (high vs low)	Hold-out	Clinical videos	Acc global phase 88.8%; Precision/Recall 88.8%/88.8%; per phase : F1 0.96 (P3), F1 0.59 (P4). Skill: less frames high-score ($p<0.01$ and $p=0.01$); AICS	Accuracy, Precision, Recall, F1; correlation with ESSQS	Black-box CNN, heterogeneous centres; n

							mayor in high-score (0.975 vs 0.970, p=0.04).		
[110]	Sasaki (2022)	10.1016/j.ijsu.2022.106856	Exception CNN (frame-wise) para 8-step (Model 1) y 6-step (Model 2, with “Step IP”)	LH 40 cases (NCCHE, Japan): 30 train / 10 test; >8 M frames; 9 pasos (0–8), exclusion Step 0	Hold-out	Clinical videos, real-time	Acc global: 0.891 (8-step) → 0.947 (6-step); Median per case 0.927 (range 0.884–0.997) in 6-step; ~21 fps.	Accuracy (global and per case), F1 per step	Single-center; model frame-wise (limited time); no external validation.
[111]	Madani (2022)	10.1097/SLA.0000000000004594	PSPNet segmentation: CholeNet	LC 290 videos (37 countries, 136 institutions, 153 surgeons); 2627 frames (10 per video); 10-fold CV	10-fold cross-val (per video)	Clinical videos	Go: IoU 0.53, F1 0.70, Acc 0.94, Sens 0.69, Spec 0.94. No-Go: IoU 0.71, F1 0.83, Acc 0.95, Sens 0.80, Spec 0.98. Anatomy (IoU): liver 0.86, gallbladder 0.72, Calot 0.65.	IoU, F1, Accuracy, Sensitivity, Specificity	No external or intra-op validation
[112]	Cheng (2022)	10.1007/s00464-021-08619-3	CNN (visual) + LSTM (temporal) for phase recognition (6 phases)	163 LC videos (4 centres): 90 train, 10 test, 63 analysis; 1 fps frame extraction	Internal hold-out (per-video)	Clinical videos	Overall accuracy 0.9105; per-phase F1 (examples): MHT 0.9407, DGB 0.9006, EA 0.9198	Accuracy, Precision, Recall, F1	Single internal test set; no external dataset; label variability across centres

[113]	Golany (2021)	10.1007/s00464-022-09405-5	Two-stage ResNet-50 + MS-TCN for 10 phases; complexity-aware	371 LC videos (5 hospitals; incl. adverse events)	Internal test (held-out)	Clinical videos	Mean accuracy 89% (95% CI 87.1–90.6); by complexity: level-1 92%, level-3 88%, level-5 81%	Accuracy (frame-wise)	Worsen with complexity. No external prospective validation.
[114]	Shinozuka (2022)	10.1007/s00464-022-09160-7	EfficientNet-B7 (CNN) + post-processing (probability & frequency gating)	115 LC; 106 for train/val (90/16), 9 for evaluation (5 fps)	Internal (eval set per-video)	Clinical videos	Accuracy 0.970, Precision 0.855, Recall 0.863	Accuracy, Precision, Recall	Small set. No external validation.
[115]	Laplante (2023)	10.1007/s00464-022-09439-9	GoNoGoNet (PSPNet-like DNN) — semantic segmentation	25 LC videos (5 countries, 9 surgeons) → 47 frames; extern panel SAGES for consensus pixel-wise	External	Clinical videos	Dice F1: Go 0.58, No-Go 0.80; Accuracy ~0.92; Sensitivity/Specificity: Go 0.52/0.97, No-Go 0.80/0.95; PPV/NPV: Go 0.70/0.94, No-Go 0.84/0.95	Dice/F1, Accuracy, Sensitivity, Specificity, PPV, NPV	Selected frame validation. No clinical prospective impact.
**Surgical decision support and outcome prediction**									
[116]	Lopez (2024)	10.1007/s00464-024-10681-6	Multiple algorithms tested (MLP, RF, SVR, KNN, LDA, ElasticNet); MLP best for complexity, RF best for outcomes; SHAP for explainability	585 laparoscopic liver resections (segments 7–8) from 19 centers, 22 pre-/intra-operative variables	Internal split (70/30 train/test, 10-fold CV)	Clinical data	Best MAE performance; MLP & RF gave highest relevance to “resection type” and “tumor size”	MAE, MSE, RMSE	Retrospective dataset; internal validation only; moderate interpretability limits

[117]	Masum (2022)	10.1007/s12672-022-00472-7	SVR (LOS), BI-LSTM (readmission, mortality)	4336 colorectal resections (2003-2019, UK NHS Trust), 47 variables	Internal 80/20 split + 10-fold CV	Clinical registry	LOS accuracy 83%, MAE 9.7 d; Readmission accuracy 87.5%, Se 84%, Sp 90%; Mortality 80–96% accuracy	Accuracy, MAE, RMSE, Sensitivity, Specificity	Single-institution data; retrospective; unbalanced events; no external validation
[118]	Lopez-Lopez (2022)	10.1007/s11605-022-05398-7	Decision tree (initial repair success) + Random forest regression (CCI-based risk score)	748 IBDI cases (22 centers, Spain, 1990–2020); 40 variables	External validation (15 development, 7 validation hospitals)	Clinical multicenter registry	Decision-tree accuracy 82.8%; RF model accuracy 82.3% (dev) / 71.7% (val)	Accuracy, AUC, F1, Sensitivity, Specificity, MAE, RMSE	Retrospective; long data span; potential variability among centers
[119]	Zheng (2023)	10.3748/wjg.v29.i3.536	Mask R-CNN segmentation + 3D CNN (C3D) classifier; integrated clinical–image model	9476 MRI images from 328 rectal cancer patients (train 260/test 68)	Internal (train/test split 4:1)	Clinical MRI data	Integrated model: accuracy 94.1%, PPV 87.5%, AUC 0.88 (vs. clinical AUC 0.72; image AUC 0.81)	Accuracy, AUC, PPV, Sensitivity, Specificity	Single-center retrospective; no external/generalization test; small sample for ≥3 firings
[120]	Dayan (2024)	10.1007/s00464-024-10847-2	Theator Surgical Intelligence Platform (AI computer vision) for automatic complexity grading and critical view of safety (CVS) recognition	499 laparoscopic appendectomy videos (Sept 2020 – May 2022, single center, Israel) – manual grading by two expert surgeons for validation	Internal (manual vs AI agreement)	Clinical videos	Complexity accuracy 76.9–94.4%; kappa 0.91 (0.88–0.94); CVS achievement accuracy 99.8% (full)	Accuracy, Cohen's kappa	Single-center retrospective; commercial black-box model; limited external generalization

[121]	Arpaia (2022)	10.1038/s41598-022-16030-8	Feed-Forward Neural Network (FFNN) + MOSSE ROI tracker for ICG fluorescence perfusion classification	Laparoscopic colorectal surgery videos (ICG angiography) from University Hospital Federico II – ROI-based frame analysis	10-fold cross-validation	Clinical videos	Classification accuracy 99.9%; repeatability 1.9%	Accuracy, Repeatability	Proof-of-concept; small dataset; no external validation or clinical impact analysis
[122]	Gillani (2024)	10.1016/j.surg.2024.08.015	ML-enabled Objective Performance Indicators (OPIs) from robotic kinematic and video data (Intuitive Surgical system)	39 robotic proctectomies, 1880 steps (1166 critical), 174,900 OPI data points; Emory University + Intuitive Surgical	Internal (statistical comparison between groups)	Clinical robotic surgery data	Significant step-specific OPI differences ($p < 0.05$) e.g., longer camera path and arm movements in obese patients	Velocity, Acceleration, Path Length, Energy Activation, Jerk	Feasibility study; small sample; no predictive model or outcome correlation
[123]	Emile (2024)	10.1007/s13304-024-01915-2	Logistic regression model + AI-generated predictive calculator (ChatGPT script) for conversion risk	26,546 colon cancer colectomies (2015–2019 NCDB data); laparoscopic 79.1%, robotic 20.9%	Internal (80/20 split with AUC analysis)	Clinical registry	AUC 0.627; conversion rate 10.6%; OR (rob vs lap) = 0.50 ($p < 0.001$); male sex OR 1.19; stage III OR 1.47	AUC, OR, Sensitivity	Retrospective database; no external validation; AI role limited to code generation
[124]	Wang (2024)	10.3748/wjg.v30.i43.4669	LASSO regression, Random Forest, and Artificial Neural Network models predicting complications post-LDG/LTG	1396 patients (998 train, 398 validation) from multiple centers; gastric cancer laparoscopic gastrectomy	Internal (train/validation)	Clinical multicenter	LTG group: RF AUC 0.885 (train), 0.903 (val); LDG group: AUC 0.923 (train), 0.787 (val)	AUC, Sensitivity, Accuracy	Secondary analysis described in a letter; limited methodological detail; lacks external test
[125]	Velmahos (2023)	10.1177/00031348231167397	Random Forest, XGBoost, L1–L2-RFE, vs Logistic Regression for 30-day morbidity	94,530 LC cases (ACS–NSQIP 2017–2019); 88 final predictors	Internal (75/25 split; 5-fold CV)	Clinical registry	AUC any morbidity: RF 0.709, XGB 0.712, L1–L2-RFE 0.712, LR 0.712; best for septic shock AUC ~0.91	AUC	NSQIP limitations (missing data, low granularity); ML ≈ LR; no external validation

[126]	Jo (2025)	10.1016/j.hpb.2025.02.016	Random Forest, SVM, XGB, and Logistic Regression indication model for LRLR vs ORLR	221 patients (110 LRLR, 111 ORLR; 2017–2021, Samsung MC)	Internal (hold-out 1:1 + 10-fold CV)	Clinical cases	RF AUC 0.779 (hold-out) / 0.720 (CV); LR AUC 0.725 (hold-out) / 0.733 (CV); p=0.71 (ns)	AUC	Single-center retrospective; limited generalization; moderate accuracy
[127]	Li (2024)	10.1016/j.surg.2024.108999	Vision Transformer (ViT) combined with ResNet-50 backbone; interpretable AI framework for bleeding risk estimation during laparoscopic colorectal procedures	149 surgical videos, labeled bleeding/non-bleeding events (frame level); augmented dataset for training	Internal 5-fold cross-validation + hold-out	Clinical intraoperative videos	AUC 0.94, Accuracy 0.91, Sensitivity 0.88, Specificity 0.93	AUC, Accuracy, Sensitivity, Specificity	Limited external validation; restricted to colorectal procedures; model generalization not tested
[128]	Cai (2023)	10.3748/wjg.v29.i3.536	Mask R-CNN + 3D Convolutional Network (C3D); integrated model combining MRI and clinical variables	9476 MRI images from 328 patients with mid-low rectal cancer undergoing laparoscopic LAR with DST anastomosis	Internal (train/test split 4:1)	Retrospective clinical cohort	AUC 0.88, Accuracy 94.1%, Sensitivity 70%, Specificity 98.3%, PPV 87.5%	AUC, accuracy, PPV, sensitivity, specificity	Single-center data, no external validation, limited to FSE T2-weighted MRI, retrospective design
[129]	Lippenberger (2024)	10.1007/s00384-024-04593-z	Random Forest classifier vs. multiclass logistic regression to predict short/intermediate/long surgery duration from CT anatomic & demographic data	85 laparoscopic sigmoid resections; single center (2009–2020); 75/25 train/test split	Internal (train/test split)	Clinical retrospective data	RF: mean AUROC 0.78 (macro), 0.73 (micro); Long AUROC 0.89, Short AUROC 0.81; Accuracy 0.55	AUROC, Accuracy, Sensitivity, Specificity	Single center; small cohort; internal validation only; lacks external replication

[130]	Zhou (2024)	10.1016/j.heliyon.2024.e26580	Ten algorithms (LR, DT, GBDT, Linear SVC, XGB, Neural Decision Tree, KNN, AdaBoost, LSTM, CNN-LSTM); best = XGB & Decision Tree	637 laparoscopic colorectal cancer cases (BioStudies public DB); POI incidence 19.1%	Internal (7:3 train/test + 5-fold CV)	Clinical dataset (retrospective)	Test set: Accuracy 0.807, AUC 0.678 (GBDT), 0.638 (XGB), Precision 0.5, Recall 0.135–0.243	AUC, Accuracy, Precision, Recall, F1	Retrospective; no external data; limited recall performance; potential data imbalance
**Augmented reality and navigation**									
[131]	Aoyama (2024)	10.1007/s00464-024-11117-x	HyperSeg semantic segmentation (nested U-Net–based) for real-time identification of pancreatic "dimpling lines" (DLs) – DMP, DIP, DTP	2771 annotated frames from 50 LG cases (45 train / 5 validation) at Oita University; validated in 10 prospective clinical cases	Internal + External (prospective validation)	Clinical intraoperative videos	Dice coefficient (pancreas) 0.70; mean Likert score for DMP indication significantly higher than DIP/DTP ($p<0.001$); display delay 210 ms	Dice, Likert score, Delay time	Limited sample (n=10 prospective); single institution; moderate interobserver agreement (Kendall's W=0.57)
[132]	Du (2022)	10.1186/s12893-022-01585-0	Fisher Linear Discriminant + Graph-cut algorithms for 3D segmentation and registration; manual vs automatic fusion with optical tracking	Preclinical simulator: rigid + phantom models, 10 surgeons; 2 pilot laparoscopic cases (PD + PS)	Preclinical + pilot clinical	Simulator + 2 live cases	3D fusion accuracy "Good" in all 40 tests; NASA-TLX total score 25–31; significant difference between manual vs automatic fusion ($p=0.026$)	Qualitative fusion accuracy, NASA-TLX workload	Preclinical only; 2 pilot cases; optical tracking impractical intraoperatively; no quantitative image error metric
[133]	Kasai (2023)	10.7759/cureus.48450	Detectron2 (Mask R-CNN) for liver silhouette detection + Vuforia SDK (AR) + Unity integration for 3D projection mapping	380 annotated liver frames for training; 5 clinical laparoscopic videos for testing (portal segment mapping)	Internal test (video-level)	Clinical laparoscopic liver surgery videos	Mean registration error 14.5 mm (AI) vs 31.2 mm (non-AI); range 4–22 mm vs 12–55 mm	Registration error (mm)	Tested in 5 videos only; rigid registration; no deformable modeling; limited to pre-dissection phase

[134]	Ryu (2024)	10.1007/s10895-024-04030-y	Eureka AI platform (computer vision model for nerve and loose connective tissue recognition) + NIR fluorescent ureteral catheter (NIRFUC)	56 laparoscopic colorectal videos (Nov 2022–May 2024) with NIRFUC; retrospective video analysis	Internal (expert visual scoring)	Clinical intraoperative videos	Mean Likert scores: ureter–LCT = 3.99; ureter–hypogastric = 3.11; ureter–lumbar = 3.53	Likert scale (0–4)	Feasibility study; no quantitative accuracy; training data lacked NIR images
[135]	Garcia-Granero (2023)	10.1016/j.ciresp.2022.10.023	3D image-processing + reconstruction (3D-IPR) from CT for surgical planning; AI-assisted anatomical and vascular mapping	2 laparoscopic colon cancer cases (splenic-flexure T3N+M0; right-colon T4bN+M0)	Case report (qualitative)	Clinical surgical cases	Correct prediction of vascular territories and D3 field; pathology confirmed T3N1b (3/18) with node in IMV zone	Feasibility, anatomical concordance	Only 2 cases; qualitative validation; no quantitative metrics; single center
[136]	Guan (2023)	10.1007/s11548-023-02846-w	Deep point-cloud registration network using mixed local + global features and overlap-mask learning	13 porcine liver surface pairs (DePoLL dataset; 2048 points per cloud)	Internal (DePoLL)	Ex vivo porcine	Target registration error 19.9 ± 2.7 mm, better than baselines	TRE (mm)	Rigid only; no human validation; small dataset
[137]	Ali (2024)	10.1016/j.media.2024.103371	Multiple DL models (UNet, CASENet, differentiable rendering) for 3D–2D registration in AR (MICCAI P2ILF challenge)	11-patient dataset (9 train, 2 test; 183 laparoscopic images)	External (multi-team challenge)	Clinical laparoscopic images	Only 1/6 teams reached < 1 cm TRE	Dice, TRE, Hausdorff distance	Small dataset; high heterogeneity; no prospective validation

[138]	Robu (2017)	10.1007/s11548-017-1584-7	Viewpoint-scoring algorithm (Gaussian curvature-based) for optimized 3D–3D rigid registration	Simulated CT + 1 clinical case; multiple virtual camera positions	Simulation + pilot clinical	Clinical laparoscopic video	Comparable accuracy using 2 high-score vs 4 low-score views	TRE, viewpoint score	Simulation framework; small clinical dataset; no deformable reg.
[139]	Wei (2024)	10.1109/TBME.2022.3195027	Learning-based stereoscopic depth estimation + dense surfel reconstruction + coarse-to-fine localization	Three datasets: (1) SCARED (stereo endoscopic), (2) ex vivo UR + Karl Storz laparoscope, (3) in vivo DaVinci robotic data	Internal (multi-dataset comparison)	Ex vivo + in vivo robotic data	Reconstruction error <1.71 mm; accurate laparoscope tracking from images only	Depth error (mm), Localization accuracy	No external clinical validation; primarily research datasets; computational intensity
[140]	Nicolaou (2005)	10.1007/11566489_4	Shadow enhancement algorithm (intensity gain, RGB vector angle, color-difference filters) for dynamic depth cueing	Box trainer with silicon model; 10 participants, 36 laparoscopic images (half enhanced vs non-enhanced)	Experimental (simulator study)	Ex vivo / simulated laparoscopic setup	Mean distance estimation error: 1.02 cm (enhanced) vs 1.36 cm (raw) ($p = 0.115$ overall); for <1 cm distances: 0.709 cm vs 1.283 cm ($p = 0.020$)	Depth perception error (cm), task time (s)	Simulated environment; small sample ($n = 10$); no clinical trial
[141]	Calinon (2014)	10.1016/j.cmpb.2013.12.015	Context-dependent reward-weighted learning (inverse reinforcement learning + Gaussian Mixture Regression) for skill transfer to STIFF-FLOP soft robot	Simulated + ex vivo training data: 7-DOF Barrett WAM teleoperator → 9-DOF soft robot; cutting and navigation tasks	Simulation (self-refinement)	Ex vivo / robotic simulation	Successful transfer of teleoperated cutting motion; smooth policy convergence; improved via-point trajectory accuracy	Reward convergence, trajectory error	Simulation only; no clinical test; performance qualitative; limited prototype validation
**Image enhancement**									

[142]	Zheng (2023)	10.1007/s11548-022-02777-y	LVQIS: integrated CNN + GAN framework; two ResNet-50 classifiers (blur/smoke detection), MPRNet for de-blurring, GAN for de-smoke/fog	136 laparoscopic cholecystectomy videos from Cholec80, M2CAI16; 19,245 synthetic blur, smoke/fog, and clear frames	Internal + clinical (transfer learning)	Clinical deployment in 100 partial nephrectomies (n=50 LVQIS vs 50 control)	De-smoke: PSNR 29.67, SSIM 0.9551, FID 74.72; De-blur: PSNR 26.78, SSIM 0.9020; Reduced pause time (6.54 → 4.37 min, p<0.001)	PSNR, SSIM, FID, surgical pause time, anxiety score	Synthetic training; limited clinical cases; single-center; only 2 surgeons
[143]	Cheng (2022)	10.1155/2022/2752444	Thread image edge detection algorithm (Canny + subpixel interpolation) for enhanced visualization in Da Vinci robotic and laparoscopic gastrectomy	154 gastric cancer cases (89 laparoscopic vs 65 robotic; 1:1 matched n=104)	Retrospective + prospective observational	Clinical (Da Vinci robotic vs laparoscopic)	Image quality coefficient Q = 0.9543; diagnostic accuracy ↑ to > 95%; intraoperative bleeding ↓ (p<0.05); 83% vs 76% 2-year OS (n.s.)	Sensitivity, specificity, accuracy, surgical outcomes	No external dataset; algorithm detail but not reproducible; single-center; no blinded assessment
[144]	Akbari (2009)	10.1109/IEMBS.2009.5333766	Image-guided pulsation detection: subtraction of systolic – diastolic frames to highlight arterial movement; signal-based segmentation displayed in real time	35 laparoscopic cholecystectomy patients; intraoperative videos acquired during live procedures	Internal (clinical feasibility test)	Clinical intraoperative videos	Correctly detected all superficial arteries; 0 false-negative, 12 % false-positive (standard mode); 4 % false-positive (enhanced mode)	Sensitivity, Specificity, False-positive rate	Limited to superficial arteries; small sample (n = 35); single-center; processing latency in enhanced mode
[145]	Katic (2013)	10.1016/j.compmedimag.2013.03.003	Ontology-based context-aware AR using description logic + fuzzy interpretation; phase recognition from sensor and video data	Phantom + pig liver models; laparoscopic liver and cholecystectomy setups	Simulation + ex vivo	Phantom and ex vivo laparoscopic setups	Correct contextual visualization in >90% of phase transitions; latency <500 ms	Recognition accuracy, latency	No clinical validation; limited sample; qualitative outcomes

[146]	Beyersdorffer (2021)	10.1515/bmt-2020-0106	ResNet-50 CNN for binary classification of instrument in/out of field	6 training LCs (porcine) + Cholec80 dataset (full annotation, 3 fps)	Internal + external	Training center + Cholec80 clinical videos	Accuracy 0.88 (training) and 0.84 (Cholec80)	Accuracy, Precision, Recall	Binary task only; simulated + limited clinical validation
[147]	Salazar-Colores (2022)	10.24875/CIRU.20000951	Hybrid GAN + Dark Channel Prior (DCP) for surgical smoke removal (pixel-to-pixel mapping)	Synthetic laparoscopic images with artificial smoke, augmented dataset	Internal (quantitative comparison)	Simulated laparoscopic imagery	PSNR > 30 dB, SSIM > 0.95, outperforming DCP or GAN alone	PSNR, SSIM	Synthetic data only; not tested intraoperatively
[148]	Wagner (2021)	10.1007/s00464-021-08509-8	Cognitive robot for camera guidance: random forests + knowledge-base ontology + feedback learning	20 human-guided training ops; phantom rectal resections with KUKA LWR + VIKY EP	Internal (iterative self-learning)	Phantom rectal resection	Duration ↓ (1704 s → 1197 s); “Good” camera guidance ↑ (38.6 → 56.2 %)	Task time, guidance quality	Phantom only; single-surgeon training; not tested in vivo
[149]	He (2025)	10.1007/s00464-025-11693-6	XGBoost regression model + biophysical modeling (RANSAC + nonlinear least squares) for fluorescence perfusion quantification	68 rectal cancer cases, 1263 points; laparoscopic ICG perfusion videos	Internal + clinical correlation	Clinical intraoperative videos	RMSE = 2.47, MAE = 1.99, R^2 = 97.2%; perfusion score correlated with complications ($p < 0.001$)	RMSE, MAE, R^2, correlation p-value	Single center; retrospective model training; no external validation
**Surgeon perception, preparedness, and attitudes**									
[150]	Acosta (2025)	10.1016/j.ciresp.2024.12.003	Descriptive statistical analysis of national closed survey; logistic regression for associations between robotic practice, digital interest,	1,086 surgeons invited; 396 responses (36.5 %); Spanish Association of Surgeons members; February–March 2024	Internal (cross-sectional survey)	Human survey data	98.3 % use EMRs; 45.4 % lack structured data systems; 55.9 % have robotic access but 70.6 % do not use it directly; digital knowledge score 7.2 vs 6.6 (p =	Proportion (%), p-value association, OR for research interest	Self-reported data; non-random sampling; no external validation; limited generalizability

			and research involvement				0.215); interest in digital tech p = 0.023		
[151]	Luense (2023)	10.1007/ s00423-023-03134-6	Descriptive statistical analysis of national web-based questionnaire; Mantel–Haenszel chi-square and Cochran–Armitage tests	2,686 invited, 202 valid responses (8%); surgeons from 38 university hospitals + 66 CLINOTEL hospitals in Germany	Internal (cross-sectional survey)	Human survey data	73.8% report inappropriate camera movement; 73.3% lens condensation; 81.2% request intuitive maneuverability; 71.3% desire AI-assisted camera positioning; 86.1% cite patient safety as reason for AI adoption	Proportion (%), p-value association	Low response rate (8%); non-representative sample; self-reported data
[152]	Shafiei (2024)	10.1177/ 00187208241285513	eXtreme Gradient Boosting (XGBoost) regression models integrating EEG and eye-tracking features	26 participants performing Matchboard, Ring Walk (da Vinci simulator) and Pattern Cut, Suturing (FLS tasks)	Internal (cross-validation per task)	151	Shafiei (2024)	10.1177/ 00187208241285513	eXtreme Gradient Boosting (XGBoost) regression models integrating EEG and eye-tracking features

Supplementary Table S2. Study-level AI Model Characteristics and Performance Metrics.

Bibliography

1. Khalid, M.U.; Laplante, S.; Masino, C.; Alseidi, A.; Jayaraman, S.; Zhang, H.; Mashouri, P.; Protserov, S.; Hunter, J.; Brudno, M.; et al. Use of Artificial Intelligence for Decision-Support to Avoid High-Risk Behaviors during Laparoscopic Cholecystectomy. *Surg Endosc* 2023, *37*, 9467–9475, doi:10.1007/S00464-023-10403-4.

2. Ward, T.M.; Hashimoto, D.A.; Ban, Y.; Rosman, G.; Meireles, O.R. Artificial Intelligence Prediction of Cholecystectomy Operative Course from Automated Identification of Gallbladder Inflammation. *Surg Endosc* 2022, *36*, 6832–6840, doi:10.1007/S00464-022-09009-Z.

3. Orimoto, H.; Hirashita, T.; Ikeda, S.; Amano, S.; Kawamura, M.; Kawano, Y.; Takayama, H.; Masuda, T.; Endo, Y.; Matsunobu, Y.; et al. Development of an Artificial Intelligence System to Indicate Intraoperative Findings of Scarring in Laparoscopic Cholecystectomy for Cholecystitis. *Surg Endosc* 2025, *39*, 1379–1387, doi:10.1007/S00464-024-11514-2.

4. Kolbinger, F.R.; Rinner, F.M.; Jenke, A.C.; Carstens, M.; Krell, S.; Leger, S.; Distler, M.; Weitz, J.; Speidel, S.; Bodenstedt, S. Anatomy Segmentation in Laparoscopic Surgery: Comparison of Machine Learning and Human Expertise - an Experimental Study. *Int J Surg* 2023, *109*, 2962–2974, doi:10.1097/JS9.0000000000000595.

5. Sato, Y.; Sese, J.; Matsuyama, T.; Onuki, M.; Mase, S.; Okuno, K.; Saito, K.; Fujiwara, N.; Hoshino, A.; Kawada, K.; et al. Preliminary Study for Developing a Navigation System for Gastric Cancer Surgery Using Artificial Intelligence. *Surg Today* 2022, *52*, 1753–1758, doi:10.1007/S00595-022-02508-5.

6. Igaki, T.; Kitaguchi, D.; Kojima, S.; Hasegawa, H.; Takeshita, N.; Mori, K.; Kinugasa, Y.; Ito, M. Artificial Intelligence-Based Total Mesorectal Excision Plane Navigation in Laparoscopic Colorectal Surgery. *Dis Colon Rectum* 2022, *65*, E329–E333, doi:10.1097/DCR.0000000000002393.

7. Jearanai, S.; Wangkulangkul, P.; Sae-Lim, W.; Cheewatanakornkul, S. Development of a Deep Learning Model for Safe Direct Optical Trocar Insertion in Minimally Invasive Surgery: An Innovative Method to Prevent Trocar Injuries. *Surg Endosc* 2023, *37*, 7295–7304, doi:10.1007/S00464-023-10309-1.

8. Oh, N.; Kim, B.; Kim, T.; Rhu, J.; Kim, J.; Choi, G.S. Real-Time Segmentation of Biliary Structure in Pure Laparoscopic Donor Hepatectomy. *Sci Rep* 2024, *14*, 22508, doi:10.1038/S41598-024-73434-4.

9. Benavides, D.; Cisnal, A.; Fontúrbel, C.; de la Fuente, E.; Fraile, J.C. Real-Time Tool Localization for Laparoscopic Surgery Using Convolutional Neural Network. *Sensors* 2024, *24*, 4191, doi:10.3390/s24134191.

10. Gazis, A.; Karaiskos, P.; Loukas, C. Surgical Gesture Recognition in Laparoscopic Tasks Based on the Transformer Network and Self-Supervised Learning. *Bioengineering* 2022, *9*, 737, doi:10.3390/bioengineering9120737.

11. Tomioka, K.; Aoki, T.; Kobayashi, N.; Tashiro, Y.; Kumazu, Y.; Shibata, H.; Hirai, T.; Yamazaki, T.; Saito, K.; Yamazaki, K.; et al. Development of a Novel Artificial Intelligence System for Laparoscopic Hepatectomy. *Anticancer Res* 2023, *43*, 5235–5243, doi:10.21873/ANTICANRES.16725.

12. Cui, P.; Zhao, S.; Chen, W. Identification of the Vas Deferens in Laparoscopic Inguinal Hernia Repair Surgery Using the Convolutional Neural Network. *J Healthc Eng* 2021, *2021*, 1–10, doi:10.1155/2021/5578089.

13. Memida, S.; Miura, S. Identification of Surgical Forceps Using YOLACT++ in Different Lighted Environments. In Proceedings of the 2023 45th Annual International Conference of the IEEE Engineering in Medicine & Biology Society (EMBC); IEEE, July 24 2023; pp. 1–4.

14. Nwoye, C.I.; Mutter, D.; Marescaux, J.; Padoy, N. Weakly Supervised Convolutional LSTM Approach for Tool Tracking in Laparoscopic Videos. *Int J Comput Assist Radiol Surg* 2019, *14*, 1059–1067, doi:10.1007/S11548-019-01958-6.

15. Jurosch, F.; Wagner, L.; Jell, A.; Islertas, E.; Wilhelm, D.; Berlet, M. Extra-Abdominal Trocar and Instrument Detection for Enhanced Surgical Workflow Understanding. *Int J Comput Assist Radiol Surg* 2024, doi:10.1007/S11548-024-03220-0.

16. Sánchez-Brizuela, G.; Santos-Criado, F.J.; Sanz-Gobernado, D.; de la Fuente-López, E.; Fraile, J.C.; Pérez-Turiel, J.; Cisnal, A. Gauze Detection and Segmentation in Minimally Invasive Surgery Video Using Convolutional Neural Networks. *Sensors* 2022, *22*, doi:10.3390/S22145180.

17. Lai, S.-L.; Chen, C.-S.; Lin, B.-R.; Chang, R.-F. Intraoperative Detection of Surgical Gauze Using Deep Convolutional Neural Network. *Ann Biomed Eng* 2023, *51*, 352–362, doi:10.1007/s10439-022-03033-9.

18. Ehrlich, J.; Jamzad, A.; Asselin, M.; Rodgers, J.R.; Kaufmann, M.; Haidegger, T.; Rudan, J.; Mousavi, P.; Fichtinger, G.; Ungi, T. Sensor-Based Automated Detection of Electrosurgical Cautery States. *Sensors* 2022, *22*, doi:10.3390/S22155808.

19. Carstens, M.; Rinner, F.M.; Bodenstedt, S.; Jenke, A.C.; Weitz, J.; Distler, M.; Speidel, S.; Kolbinger, F.R. The Dresden Surgical Anatomy Dataset for Abdominal Organ Segmentation in Surgical Data Science. *Sci Data* 2023, *10*, 3, doi:10.1038/s41597-022-01719-2.

20. Yin, Y.; Luo, S.; Zhou, J.; Kang, L.; Chen, C.Y.C. LDCNet: Lightweight Dynamic Convolution Network for Laparoscopic Procedures Image Segmentation. *Neural Networks* 2024, *170*, 441–452, doi:10.1016/J.NEUNET.2023.11.055.

21. Tashiro, Y.; Aoki, T.; Kobayashi, N.; Tomioka, K.; Saito, K.; Matsuda, K.; Kusano, T. Novel Navigation for Laparoscopic Cholecystectomy Fusing Artificial Intelligence and Indocyanine Green Fluorescent Imaging. *J Hepatobiliary Pancreat Sci* 2024, *31*, 305–307, doi:10.1002/JHBP.1422;REQUESTEDJOURNAL:JOURNAL:18686982;JOURNAL:JOURNAL:18686982A;WGROUP:STRING:PUBLICATION.

22. Petracchi, E.J.; Olivieri, S.E.; Varela, J.; Canullan, C.M.; Zandalazini, H.; Ocampo, C.; Quesada, B.M. Use of Artificial Intelligence in the Detection of the Critical View of Safety during

Laparoscopic Cholecystectomy. *Journal of Gastrointestinal Surgery* 2024, *28*, 877–879, doi:10.1016/J.GASSUR.2024.03.018.

23. Schnelldorfer, T.; Castro, J.; Goldar-Najafi, A.; Liu, L. Development of a Deep Learning System for Intra-Operative Identification of Cancer Metastases. *Ann Surg* 2024, doi:10.1097/SLA.0000000000006294.

24. Kitaguchi, D.; Harai, Y.; Kosugi, N.; Hayashi, K.; Kojima, S.; Ishikawa, Y.; Yamada, A.; Hasegawa, H.; Takeshita, N.; Ito, M. Artificial Intelligence for the Recognition of Key Anatomical Structures in Laparoscopic Colorectal Surgery. *British Journal of Surgery* 2023, *110*, 1355–1358, doi:10.1093/BJS/ZNAD249.

25. Chen, G.; Xie, Y.; Yang, B.; Tan, J.N.; Zhong, G.; Zhong, L.; Zhou, S.; Han, F. Artificial Intelligence Model for Perigastric Blood Vessel Recognition during Laparoscopic Radical Gastrectomy with D2 Lymphadenectomy in Locally Advanced Gastric Cancer. *BJS Open* 2025, *9*, doi:10.1093/BJSOPEN/ZRAE158.

26. Han, F.; Zhong, G.; Zhi, S.; Han, N.; Jiang, Y.; Tan, J.; Zhong, L.; Zhou, S. Artificial Intelligence Recognition System of Pelvic Autonomic Nerve During Total Mesorectal Excision. *Dis Colon Rectum* 2024, doi:10.1097/DCR.0000000000003547.

27. Tashiro, Y.; Aoki, T.; Kobayashi, N.; Tomioka, K.; Kumazu, Y.; Akabane, M.; Shibata, H.; Hirai, T.; Matsuda, K.; Kusano, T. Color-Coded Laparoscopic Liver Resection Using Artificial Intelligence: A Preliminary Study. *J Hepatobiliary Pancreat Sci* 2024, *31*, 67–68, doi:10.1002/JHBP.1388;REQUESTEDJOURNAL:JOURNAL:18686982;JOURNAL:JOURNAL:18686982A;WGROUP:STRING:PUBLICATION.

28. Frey, S.; Facente, F.; Wei, W.; Ekmekci, E.S.; Séjor, E.; Baqué, P.; Durand, M.; Delingette, H.; Bremond, F.; Berthet-Rayne, P.; et al. Optimizing Intraoperative AI: Evaluation of YOLOv8 for Real-Time Recognition of Robotic and Laparoscopic Instruments. *J Robot Surg* 2025, *19*, doi:10.1007/S11701-025-02284-7.

29. ElMoaqet, H.; Janini, R.; Ryalat, M.; Al-Refai, G.; Abdulbaki Alshirbaji, T.; Jalal, N.A.; Neumuth, T.; Moeller, K.; Navab, N. Using Masked Image Modelling Transformer Architecture for Laparoscopic Surgical Tool Classification and Localization. *Sensors* 2025, *25*, doi:10.3390/S25103017.

30. Korndorffer, J.R.; Hawn, M.T.; Spain, D.A.; Knowlton, L.M.; Azagury, D.E.; Nassar, A.K.; Lau, J.N.; Arnow, K.D.; Trickey, A.W.; Pugh, C.M. Situating Artificial Intelligence in Surgery: A Focus on Disease Severity. *Ann Surg* 2020, *272*, 523–528, doi:10.1097/SLA.0000000000004207.

31. Ryu, S.; Goto, K.; Kitagawa, T.; Kobayashi, T.; Shimada, J.; Ito, R.; Nakabayashi, Y. Real-Time Artificial Intelligence Navigation-Assisted Anatomical Recognition in Laparoscopic Colorectal Surgery. *Journal of Gastrointestinal Surgery* 2023, *27*, 3080–3082, doi:10.1007/S11605-023-05819-1.

32. Park, S.H.; Park, H.M.; Baek, K.R.; Ahn, H.M.; Lee, I.Y.; Son, G.M. Artificial Intelligence Based Real-Time Microcirculation Analysis System for Laparoscopic Colorectal Surgery. *World J Gastroenterol* 2020, *26*, 6945–6962, doi:10.3748/WJG.V26.I44.6945.

33. Ryu, K.; Kitaguchi, D.; Nakajima, K.; Ishikawa, Y.; Harai, Y.; Yamada, A.; Lee, Y.; Hayashi, K.; Kosugi, N.; Hasegawa, H.; et al. Deep Learning-Based Vessel Automatic Recognition for Laparoscopic Right Hemicolectomy. *Surg Endosc* 2024, *38*, 171–178, doi:10.1007/S00464-023-10524-W.

34. Zygomalas, A.; Kalles, D.; Katsiakis, N.; Anastasopoulos, A.; Skroubis, G. Artificial Intelligence Assisted Recognition of Anatomical Landmarks and Laparoscopic Instruments in Transabdominal Preperitoneal Inguinal Hernia Repair. *Surg Innov* 2024, *31*, 178–184, doi:10.1177/15533506241226502.

35. Mita, K.; Kobayashi, N.; Takahashi, K.; Sakai, T.; Shimaguchi, M.; Kouno, M.; Toyota, N.; Hatano, M.; Toyota, T.; Sasaki, J. Anatomical Recognition of Dissection Layers, Nerves, Vas Deferens, and Microvessels Using Artificial Intelligence during Transabdominal Preperitoneal Inguinal Hernia Repair. *Hernia* 2025, *29*, doi:10.1007/S10029-024-03223-5.

36. TOMIOKA, K.; AOKI, T.; KOBAYASHI, N.; TASHIRO, Y.; KUMAZU, Y.; SHIBATA, H.; HIRAI, T.; YAMAZAKI, T.; SAITO, K.; YAMAZAKI, K.; et al. Development of a Novel Artificial Intelligence System for Laparoscopic Hepatectomy. *Anticancer Res* 2023, *43*, 5235–5243, doi:10.21873/anticanres.16725.

37. Horita, K.; Hida, K.; Itatani, Y.; Fujita, H.; Hidaka, Y.; Yamamoto, G.; Ito, M.; Obama, K. Real-Time Detection of Active Bleeding in Laparoscopic Colectomy Using Artificial Intelligence. *Surg Endosc* 2024, *38*, 3461–3469, doi:10.1007/S00464-024-10874-Z.

38. Kinoshita, K.; Maruyama, T.; Kobayashi, N.; Imanishi, S.; Maruyama, M.; Ohira, G.; Endo, S.; Tochigi, T.; Kinoshita, M.; Fukui, Y.; et al. An Artificial Intelligence-Based Nerve Recognition Model Is Useful as Surgical Support Technology and as an Educational Tool in Laparoscopic and Robot-Assisted Rectal Cancer Surgery. *Surg Endosc* 2024, *38*, 5394–5404, doi:10.1007/S00464-024-10939-Z.

39. Takeuchi, M.; Collins, T.; Lipps, C.; Haller, M.; Uwineza, J.; Okamoto, N.; Nkusi, R.; Marescaux, J.; Kawakubo, H.; Kitagawa, Y.; et al. Towards Automatic Verification of the Critical View of the Myopectineal Orifice with Artificial Intelligence. *Surg Endosc* 2023, *37*, 4525–4534, doi:10.1007/S00464-023-09934-7.

40. Une, N.; Kobayashi, S.; Kitaguchi, D.; Sunakawa, T.; Sasaki, K.; Ogane, T.; Hayashi, K.; Kosugi, N.; Kudo, M.; Sugimoto, M.; et al. Intraoperative Artificial Intelligence System Identifying Liver Vessels in Laparoscopic Liver Resection: A Retrospective Experimental Study. *Surg Endosc* 2024, *38*, 1088–1095, doi:10.1007/S00464-023-10637-2.

41. Kojima, S.; Kitaguchi, D.; Igaki, T.; Nakajima, K.; Ishikawa, Y.; Harai, Y.; Yamada, A.; Lee, Y.; Hayashi, K.; Kosugi, N.; et al. Deep-Learning-Based Semantic Segmentation of Autonomic Nerves from Laparoscopic Images of Colorectal Surgery: An Experimental Pilot Study. *Int J Surg* 2023, *109*, 813–820, doi:10.1097/JS9.0000000000000317.

42. Nakanuma, H.; Endo, Y.; Fujinaga, A.; Kawamura, M.; Kawasaki, T.; Masuda, T.; Hirashita, T.; Etoh, T.; Shinozuka, K.; Matsunobu, Y.; et al. An Intraoperative Artificial Intelligence System Identifying Anatomical Landmarks for Laparoscopic Cholecystectomy: A Prospective Clinical Feasibility Trial (J-SUMMIT-C-01). *Surg Endosc* 2023, *37*, 1933–1942, doi:10.1007/S00464-022-09678-W.

43. Loukas, C.; Gazis, A.; Schizas, D. Multiple Instance Convolutional Neural Network for Gallbladder Assessment from Laparoscopic Images. *International Journal of Medical Robotics and Computer Assisted Surgery* 2022, *18*, doi:10.1002/RCS.2445.

44. Endo, Y.; Tokuyasu, T.; Mori, Y.; Asai, K.; Umezawa, A.; Kawamura, M.; Fujinaga, A.; Ejima, A.; Kimura, M.; Inomata, M. Impact of AI System on Recognition for Anatomical Landmarks Related to Reducing Bile Duct Injury during Laparoscopic Cholecystectomy. *Surg Endosc* 2023, *37*, 5752–5759, doi:10.1007/S00464-023-10224-5.

45. Fried, G.M.; Ortenzi, M.; Dayan, D.; Nizri, E.; Mirkin, Y.; Maril, S.; Asselmann, D.; Wolf, T. Surgical Intelligence Can Lead to Higher Adoption of Best Practices in Minimally Invasive Surgery. *Ann Surg* 2024, *280*, 525–534, doi:10.1097/SLA.0000000000006377.

46. Mascagni, P.; Vardazaryan, A.; Alapatt, D.; Urade, T.; Emre, T.; Fiorillo, C.; Pessaux, P.; Mutter, D.; Marescaux, J.; Costamagna, G.; et al. Artificial Intelligence for Surgical Safety Automatic Assessment of the Critical View of Safety in Laparoscopic Cholecystectomy Using Deep Learning. *Ann Surg* 2022, *275*, 955–961, doi:10.1097/SLA.0000000000004351.

47. Fujinaga, A.; Endo, Y.; Etoh, T.; Kawamura, M.; Nakanuma, H.; Kawasaki, T.; Masuda, T.; Hirashita, T.; Kimura, M.; Matsunobu, Y.; et al. Development of a Cross-Artificial Intelligence System for Identifying Intraoperative Anatomical Landmarks and Surgical Phases during Laparoscopic Cholecystectomy. *Surg Endosc* 2023, *37*, 6118–6128, doi:10.1007/S00464-023-10097-8.

48. Kawamura, M.; Endo, Y.; Fujinaga, A.; Orimoto, H.; Amano, S.; Kawasaki, T.; Kawano, Y.; Masuda, T.; Hirashita, T.; Kimura, M.; et al. Development of an Artificial Intelligence System for Real-Time Intraoperative Assessment of the Critical View of Safety in Laparoscopic Cholecystectomy. *Surg Endosc* 2023, *37*, 8755–8763, doi:10.1007/S00464-023-10328-Y.

49. Tokuyasu, T.; Iwashita, Y.; Matsunobu, Y.; Kamiyama, T.; Ishikake, M.; Sakaguchi, S.; Ebe, K.; Tada, K.; Endo, Y.; Etoh, T.; et al. Development of an Artificial Intelligence System Using Deep Learning to Indicate Anatomical Landmarks during Laparoscopic Cholecystectomy. *Surg Endosc* 2021, *35*, 1651–1658, doi:10.1007/S00464-020-07548-X.

50. Zhang, K.; Qiao, Z.; Yang, L.; Zhang, T.; Liu, F.; Sun, D.; Xie, T.; Guo, L.; Lu, C. Computer-Vision-Based Artificial Intelligence for Detection and Recognition of Instruments and Organs during Radical Laparoscopic Gastrectomy for Gastric Cancer: A Multicenter Study. *Chinese Journal of Gastrointestinal Surgery* 2024, *27*, 464–470, doi:10.3760/CMA.J.CN441530-20240125-00041.

51. Ortenzi, M.; Rapoport Ferman, J.; Antolin, A.; Bar, O.; Zohar, M.; Perry, O.; Asselmann, D.; Wolf, T. A Novel High Accuracy Model for Automatic Surgical Workflow Recognition Using Artificial Intelligence in Laparoscopic Totally Extraperitoneal Inguinal Hernia Repair (TEP). *Surg Endosc* 2023, *37*, 8818–8828, doi:10.1007/S00464-023-10375-5.

52. Wu, S.; Tang, M.; Liu, J.; Qin, D.; Wang, Y.; Zhai, S.; Bi, E.; Li, Y.; Wang, C.; Xiong, Y.; et al. Impact of an AI-Based Laparoscopic Cholecystectomy Coaching Program on the Surgical Performance: A Randomized Controlled Trial. *International Journal of Surgery* 2024, *110*, doi:10.1097/JS9.0000000000001798,.

53. Belmar, F.; Gaete, M.I.; Escalona, G.; Carnier, M.; Durán, V.; Villagrán, I.; Asbun, D.; Cortés, M.; Neyem, A.; Crovari, F.; et al. Artificial Intelligence in Laparoscopic Simulation: A Promising Future for Large-Scale Automated Evaluations. *Surg Endosc* 2023, *37*, 4942–4946, doi:10.1007/s00464-022-09576-1.

54. Halperin, L.; Sroka, G.; Zuckerman, I.; Laufer, S. Automatic Performance Evaluation of the Intracorporeal Suture Exercise. *Int J Comput Assist Radiol Surg* 2023, *19*, 83–86, doi:10.1007/s11548-023-02963-6.

55. Chen, G.; Li, L.; Hubert, J.; Luo, B.; Yang, K.; Wang, X. Effectiveness of a Vision-Based Handle Trajectory Monitoring System in Studying Robotic Suture Operation. *J Robot Surg* 2023, *17*, 2791–2798, doi:10.1007/S11701-023-01713-9.

56. Ismail Fawaz, H.; Forestier, G.; Weber, J.; Idoumghar, L.; Muller, P.A. Accurate and Interpretable Evaluation of Surgical Skills from Kinematic Data Using Fully Convolutional Neural Networks. *Int J Comput Assist Radiol Surg* 2019, *14*, 1611–1617, doi:10.1007/S11548-019-02039-4.

57. Nguyen, X.A.; Ljuhar, D.; Pacilli, M.; Nataraja, R.M.; Chauhan, S. Surgical Skill Levels: Classification and Analysis Using Deep Neural Network Model and Motion Signals. *Comput Methods Programs Biomed* 2019, *177*, 1–8, doi:10.1016/J.CMPB.2019.05.008.

58. SATR-DL: Improving Surgical Skill Assessment and Task Recognition in Robot-Assisted Surgery with Deep Neural Networks-All Databases Available online: https://www.webofscience.com/wos/alldb/full-record/WOS:000596231902067 (accessed on 25 May 2025).

59. Funke, I.; Mees, S.T.; Weitz, J.; Speidel, S. Video-Based Surgical Skill Assessment Using 3D Convolutional Neural Networks. *Int J Comput Assist Radiol Surg* 2019, *14*, 1217–1225, doi:10.1007/s11548-019-01995-1.

60. Partridge, R.W.; Hughes, M.A.; Brennan, P.M.; Hennessey, I.A.M. Accessible Laparoscopic Instrument Tracking ("InsTrac"): Construct Validity in a Take-Home Box Simulator. *Journal of Laparoendoscopic and Advanced Surgical Techniques* 2014, *24*, 578–583, doi:10.1089/LAP.2014.0015.

61. Derathé, A.; Reche, F.; Guy, S.; Charrière, K.; Trilling, B.; Jannin, P.; Moreau-Gaudry, A.; Gibaud, B.; Voros, S. LapEx: A New Multimodal Dataset for Context Recognition and Practice Assessment in Laparoscopic Surgery. *Sci Data* 2025, *12*, 342, doi:10.1038/s41597-025-04588-7.

62. Bogar, P.Z.; Virag, M.; Bene, M.; Hardi, P.; Matuz, A.; Schlegl, A.T.; Toth, L.; Molnar, F.; Nagy, B.; Rendeki, S.; et al. Validation of a Novel, Low-Fidelity Virtual Reality Simulator and an Artificial Intelligence Assessment Approach for Peg Transfer Laparoscopic Training. *Sci Rep* 2024, *14*, 16702, doi:10.1038/s41598-024-67435-6.

63. Matsumoto, S.; Kawahira, H.; Fukata, K.; Doi, Y.; Kobayashi, N.; Hosoya, Y.; Sata, N. Laparoscopic Distal Gastrectomy Skill Evaluation from Video: A New Artificial Intelligence-Based Instrument Identification System. *Sci Rep* 2024, *14*, 12432, doi:10.1038/s41598-024-63388-y.

64. Gillani, M.; Rupji, M.; Paul Olson, T.J.; Sullivan, P.; Shaffer, V.O.; Balch, G.C.; Shields, M.C.; Liu, Y.; Rosen, S.A. Objective Performance Indicators During Robotic Right Colectomy Differ According to Surgeon Skill. *Journal of Surgical Research* 2024, *302*, 836–844, doi:10.1016/J.JSS.2024.07.103.

65. Yang, J.H.; Goodman, E.D.; Dawes, A.J.; Gahagan, J. V.; Esquivel, M.M.; Liebert, C.A.; Kin, C.; Yeung, S.; Gurland, B.H. Using AI and Computer Vision to Analyze Technical Proficiency in Robotic Surgery. *Surg Endosc* 2023, *37*, 3010–3017, doi:10.1007/S00464-022-09781-Y.

66. Caballero, D.; Pérez-Salazar, M.J.; Sánchez-Margallo, J.A.; Sánchez-Margallo, F.M. Applying Artificial Intelligence on EDA Sensor Data to Predict Stress on Minimally Invasive Robotic-Assisted Surgery. *Int J Comput Assist Radiol Surg* 2024, doi:10.1007/S11548-024-03218-8.

67. Yanik, E.; Ainam, J.P.; Fu, Y.; Schwaitzberg, S.; Cavuoto, L.; De, S. Video-Based Skill Acquisition Assessment in Laparoscopic Surgery Using Deep Learning. *Global Surgical Education - Journal of the Association for Surgical Education* 2024, *3*, doi:10.1007/S44186-023-00223-4.

68. Nakajima, K.; Kitaguchi, D.; Takenaka, S.; Tanaka, A.; Ryu, K.; Takeshita, N.; Kinugasa, Y.; Ito, M. Automated Surgical Skill Assessment in Colorectal Surgery Using a Deep Learning-Based Surgical Phase Recognition Model. *Surg Endosc* 2024, doi:10.1007/S00464-024-11208-9.

69. Yamazaki, Y.; Kanaji, S.; Kudo, T.; Takiguchi, G.; Urakawa, N.; Hasegawa, H.; Yamamoto, M.; Matsuda, Y.; Yamashita, K.; Matsuda, T.; et al. Quantitative Comparison of Surgical Device Usage in Laparoscopic Gastrectomy Between Surgeons' Skill Levels: An Automated Analysis Using a Neural Network. *Journal of Gastrointestinal Surgery* 2022, *26*, 1006–1014, doi:10.1007/S11605-021-05161-4.

70. Allen, B.; Nistor, V.; Dutson, E.; Carman, G.; Lewis, C.; Faloutsos, P. Support Vector Machines Improve the Accuracy of Evaluation for the Performance of Laparoscopic Training Tasks. *Surg Endosc* 2010, *24*, 170–178, doi:10.1007/S00464-009-0556-6.

71. Fukuta, A.; Yamashita, S.; Maniwa, J.; Tamaki, A.; Kondo, T.; Kawakubo, N.; Nagata, K.; Matsuura, T.; Tajiri, T. Artificial Intelligence Facilitates the Potential of Simulator Training: An Innovative Laparoscopic Surgical Skill Validation System Using Artificial Intelligence Technology. *Int J Comput Assist Radiol Surg* 2024, doi:10.1007/S11548-024-03253-5,.

72. Moglia, A.; Morelli, L.; D'Ischia, R.; Fatucchi, L.M.; Pucci, V.; Berchiolli, R.; Ferrari, M.; Cuschieri, A. Ensemble Deep Learning for the Prediction of Proficiency at a Virtual Simulator for Robot-Assisted Surgery. *Surg Endosc* 2022, *36*, 6473–6479, doi:10.1007/S00464-021-08999-6.

73. Ju, S.; Jiang, P.; Jin, Y.; Fu, Y.; Wang, X.; Tan, X.; Han, Y.; Yin, R.; Pu, D.; Li, K. Automatic Gesture Recognition and Evaluation in Peg Transfer Tasks of Laparoscopic Surgery Training. *Surgical Endoscopy* 2025, doi:10.1007/S00464-025-11730-4.

74. Cruz, E.; Selman, R.; Figueroa, Ú.; Belmar, F.; Jarry, C.; Sanhueza, D.; Escalona, G.; Carnier, M.; Varas, J. A Scalable Solution: Effective AI Implementation in Laparoscopic Simulation Training Assessments. *Global Surgical Education - Journal of the Association for Surgical Education* 2025, *4*, 46, doi:10.1007/S44186-025-00355-9.

75. Chen, Z.; Yang, D.; Li, A.; Sun, L.; Zhao, J.; Liu, J.; Liu, L.; Zhou, X.; Chen, Y.; Cai, Y.; et al. Decoding Surgical Skill: An Objective and Efficient Algorithm for Surgical Skill Classification

Based on Surgical Gesture Features -Experimental Studies. *Int J Surg* 2024, *110*, 1441–1449, doi:10.1097/JS9.0000000000000975.

76. Erlich-Feingold, O.; Anteby, R.; Klang, E.; Soffer, S.; Cordoba, M.; Nachmany, I.; Amiel, I.; Barash, Y. Artificial Intelligence Classifies Surgical Technical Skills in Simulated Laparoscopy: A Pilot Study. *Surgical Endoscopy* 2025, doi:10.1007/S00464-025-11715-3.

77. Power, D.; Burke, C.; Madden, M.G.; Ullah, I. Automated Assessment of Simulated Laparoscopic Surgical Skill Performance Using Deep Learning. *Sci Rep* 2025, *15*, doi:10.1038/S41598-025-96336-5.

78. Alonso-Silverio, G.A.; Pérez-Escamirosa, F.; Bruno-Sanchez, R.; Ortiz-Simon, J.L.; Muñoz-Guerrero, R.; Minor-Martinez, A.; Alarcón-Paredes, A. Development of a Laparoscopic Box Trainer Based on Open Source Hardware and Artificial Intelligence for Objective Assessment of Surgical Psychomotor Skills. *Surg Innov* 2018, *25*, 380–388, doi:10.1177/1553350618777045.

79. Belmar, F.; Gaete, M.I.; Escalona, G.; Carnier, M.; Durán, V.; Villagrán, I.; Asbun, D.; Cortés, M.; Neyem, A.; Crovari, F.; et al. Artificial Intelligence in Laparoscopic Simulation: A Promising Future for Large-Scale Automated Evaluations. *Surg Endosc* 2023, *37*, 4942–4946, doi:10.1007/S00464-022-09576-1.

80. Pan, J.J.; Chang, J.; Yang, X.; Zhang, J.J.; Qureshi, T.; Howell, R.; Hickish, T. Graphic and Haptic Simulation System for Virtual Laparoscopic Rectum Surgery. *The International Journal of Medical Robotics and Computer Assisted Surgery* 2011, *7*, 304–317, doi:10.1002/rcs.399.

81. Ershad, M.; Rege, R.; Majewicz Fey, A. Automatic and near Real-Time Stylistic Behavior Assessment in Robotic Surgery. *Int J Comput Assist Radiol Surg* 2019, *14*, 635–643, doi:10.1007/S11548-019-01920-6.

82. Kowalewski, K.F.; Garrow, C.R.; Schmidt, M.W.; Benner, L.; Müller-Stich, B.P.; Nickel, F. Sensor-Based Machine Learning for Workflow Detection and as Key to Detect Expert Level in Laparoscopic Suturing and Knot-Tying. *Surg Endosc* 2019, *33*, 3732–3740, doi:10.1007/S00464-019-06667-4.

83. St John, A.; Khalid, M.U.; Masino, C.; Noroozi, M.; Alseidi, A.; Hashimoto, D.A.; Altieri, M.; Serrot, F.; Kersten-Oertal, M.; Madani, A. LapBot-Safe Chole: Validation of an Artificial Intelligence-Powered Mobile Game App to Teach Safe Cholecystectomy. *Surg Endosc* 2024, *38*, 5274–5284, doi:10.1007/S00464-024-11068-3.

84. Yen, H.H.; Hsiao, Y.H.; Yang, M.H.; Huang, J.Y.; Lin, H.T.; Huang, C.C.; Blue, J.; Ho, M.C. Automated Surgical Action Recognition and Competency Assessment in Laparoscopic Cholecystectomy: A Proof-of-Concept Study. *Surgical Endoscopy* 2025, doi:10.1007/S00464-025-11663-Y.

85. Nakajima, K.; Takenaka, S.; Kitaguchi, D.; Tanaka, A.; Ryu, K.; Takeshita, N.; Kinugasa, Y.; Ito, M. Artificial Intelligence Assessment of Tissue-Dissection Efficiency in Laparoscopic Colorectal Surgery. *Langenbecks Arch Surg* 2025, *410*, doi:10.1007/S00423-025-03641-8.

86. Igaki, T.; Kitaguchi, D.; Matsuzaki, H.; Nakajima, K.; Kojima, S.; Hasegawa, H.; Takeshita, N.; Kinugasa, Y.; Ito, M. Automatic Surgical Skill Assessment System Based on Concordance of

Standardized Surgical Field Development Using Artificial Intelligence. *JAMA Surg* 2023, *158*, E231131, doi:10.1001/JAMASURG.2023.1131.

87. Smith, R.; Julian, D.; Dubin, A. Deep Neural Networks Are Effective Tools for Assessing Performance during Surgical Training. *J Robot Surg* 2022, *16*, 559–562, doi:10.1007/S11701-021-01284-7.

88. Loukas, C.; Seimenis, I.; Prevezanou, K.; Schizas, D. Prediction of Remaining Surgery Duration in Laparoscopic Videos Based on Visual Saliency and the Transformer Network. *The International Journal of Medical Robotics and Computer Assisted Surgery* 2024, *20*, doi:10.1002/rcs.2632.

89. Wagner, M.; Müller-Stich, B.P.; Kisilenko, A.; Tran, D.; Heger, P.; Mündermann, L.; Lubotsky, D.M.; Müller, B.; Davitashvili, T.; Capek, M.; et al. Comparative Validation of Machine Learning Algorithms for Surgical Workflow and Skill Analysis with the HeiChole Benchmark. *Med Image Anal* 2023, *86*, doi:10.1016/j.media.2023.102770.

90. Zhang, B.; Goel, B.; Sarhan, M.H.; Goel, V.K.; Abukhalil, R.; Kalesan, B.; Stottler, N.; Petculescu, S. Surgical Workflow Recognition with Temporal Convolution and Transformer for Action Segmentation. *Int J Comput Assist Radiol Surg* 2023, *18*, 785–794, doi:10.1007/S11548-022-02811-Z.

91. Park, B.; Chi, H.; Park, B.; Lee, J.; Jin, H.S.; Park, S.; Hyung, W.J.; Choi, M.K. Visual Modalities-Based Multimodal Fusion for Surgical Phase Recognition. *Comput Biol Med* 2023, *166*, 107453, doi:10.1016/J.COMPBIOMED.2023.107453.

92. Twinanda, A.P.; Yengera, G.; Mutter, D.; Marescaux, J.; Padoy, N. RSDNet: Learning to Predict Remaining Surgery Duration from Laparoscopic Videos Without Manual Annotations. *IEEE Trans Med Imaging* 2019, *38*, 1069–1078, doi:10.1109/TMI.2018.2878055.

93. Zang, C.; Turkcan, M.K.; Narasimhan, S.; Cao, Y.; Yarali, K.; Xiang, Z.; Szot, S.; Ahmad, F.; Choksi, S.; Bitner, D.P.; et al. Surgical Phase Recognition in Inguinal Hernia Repair—AI-Based Confirmatory Baseline and Exploration of Competitive Models. *Bioengineering* 2023, *10*, 654, doi:10.3390/bioengineering10060654.

94. Cartucho, J.; Weld, A.; Tukra, S.; Xu, H.; Matsuzaki, H.; Ishikawa, T.; Kwon, M.; Jang, Y.E.; Kim, K.J.; Lee, G.; et al. SurgT Challenge: Benchmark of Soft-Tissue Trackers for Robotic Surgery. *Med Image Anal* 2024, *91*, doi:10.1016/J.MEDIA.2023.102985.

95. Zheng, Y.; Leonard, G.; Zeh, H.; Fey, A.M. Frame-Wise Detection of Surgeon Stress Levels during Laparoscopic Training Using Kinematic Data. *Int J Comput Assist Radiol Surg* 2022, *17*, 785–794, doi:10.1007/S11548-022-02568-5.

96. Zhai, Y.; Chen, Z.; Zheng, Z.; Wang, X.; Yan, X.; Liu, X.; Yin, J.; Wang, J.; Zhang, J. Artificial Intelligence for Automatic Surgical Phase Recognition of Laparoscopic Gastrectomy in Gastric Cancer. *Int J Comput Assist Radiol Surg* 2024, *19*, 345–353, doi:10.1007/S11548-023-03027-5.

97. Takeuchi, M.; Collins, T.; Ndagijimana, A.; Kawakubo, H.; Kitagawa, Y.; Marescaux, J.; Mutter, D.; Perretta, S.; Hostettler, A.; Dallemagne, B. Automatic Surgical Phase Recognition in Laparoscopic Inguinal Hernia Repair with Artificial Intelligence. *Hernia* 2022, *26*, 1669–1678, doi:10.1007/S10029-022-02621-X.

98. Hashimoto, D.A.; Rosman, G.; Witkowski, E.R.; Stafford, C.; Navarette-Welton, A.J.; Rattner, D.W.; Lillemoe, K.D.; Rus, D.L.; Meireles, O.R. Computer Vision Analysis of Intraoperative Video: Automated Recognition of Operative Steps in Laparoscopic Sleeve Gastrectomy. *Ann Surg* 2019, *270*, 414–421, doi:10.1097/SLA.0000000000003460.

99. You, J.; Cai, H.; Wang, Y.; Bian, A.; Cheng, K.; Meng, L.; Wang, X.; Gao, P.; Chen, S.; Cai, Y.; et al. Artificial Intelligence Automated Surgical Phases Recognition in Intraoperative Videos of Laparoscopic Pancreatoduodenectomy. *Surg Endosc* 2024, *38*, 4894–4905, doi:10.1007/S00464-024-10916-6.

100. Takeuchi, M.; Kawakubo, H.; Tsuji, T.; Maeda, Y.; Matsuda, S.; Fukuda, K.; Nakamura, R.; Kitagawa, Y. Evaluation of Surgical Complexity by Automated Surgical Process Recognition in Robotic Distal Gastrectomy Using Artificial Intelligence. *Surg Endosc* 2023, *37*, 4517–4524, doi:10.1007/S00464-023-09924-9.

101. Zheng, Q.; Yang, R.; Yang, S.; Ni, X.; Li, Y.; Jiang, Z.; Wang, X.; Wang, L.; Chen, Z.; Liu, X. Development and Validation of a Deep-Learning Based Assistance System for Enhancing Laparoscopic Control Level. *International Journal of Medical Robotics and Computer Assisted Surgery* 2023, *19*, doi:10.1002/RCS.2449.

102. Dayan, D. Implementation of Artificial Intelligence–Based Computer Vision Model for Sleeve Gastrectomy: Experience in One Tertiary Center. *Obes Surg* 2024, *34*, 330–336, doi:10.1007/S11695-023-07043-X.

103. Kitaguchi, D.; Takeshita, N.; Matsuzaki, H.; Oda, T.; Watanabe, M.; Mori, K.; Kobayashi, E.; Ito, M. Automated Laparoscopic Colorectal Surgery Workflow Recognition Using Artificial Intelligence: Experimental Research. *International Journal of Surgery* 2020, *79*, 88–94, doi:10.1016/J.IJSU.2020.05.015.

104. Yoshida, M.; Kitaguchi, D.; Takeshita, N.; Matsuzaki, H.; Ishikawa, Y.; Yura, M.; Akimoto, T.; Kinoshita, T.; Ito, M. Surgical Step Recognition in Laparoscopic Distal Gastrectomy Using Artificial Intelligence: A Proof-of-Concept Study. *Langenbecks Arch Surg* 2024, *409*, doi:10.1007/S00423-024-03411-Y.

105. Fer, D.; Zhang, B.; Abukhalil, R.; Goel, V.; Goel, B.; Barker, J.; Kalesan, B.; Barragan, I.; Gaddis, M.L.; Kilroy, P.G. An Artificial Intelligence Model That Automatically Labels Roux-En-Y Gastric Bypasses, a Comparison to Trained Surgeon Annotators. *Surg Endosc* 2023, *37*, 5665–5672, doi:10.1007/S00464-023-09870-6.

106. Liu, Y.; Zhao, S.; Zhang, G.; Zhang, X.; Hu, M.; Zhang, X.; Li, C.; Zhou, S.K.; Liu, R. Multilevel Effective Surgical Workflow Recognition in Robotic Left Lateral Sectionectomy with Deep Learning: Experimental Research. *Int J Surg* 2023, *109*, 2941–2952, doi:10.1097/JS9.0000000000000559.

107. Khojah, B.; Enani, G.; Saleem, A.; Malibary, N.; Sabbagh, A.; Malibari, A.; Alhalabi, W. Deep Learning-Based Intraoperative Visual Guidance Model for Ureter Identification in Laparoscopic Sigmoidectomy. *Surgical Endoscopy* 2025, doi:10.1007/S00464-025-11694-5.

108. Lavanchy, J.L.; Ramesh, S.; Dall'Alba, D.; Gonzalez, C.; Fiorini, P.; Müller-Stich, B.P.; Nett, P.C.; Marescaux, J.; Mutter, D.; Padoy, N. Challenges in Multi-Centric Generalization: Phase and

Step Recognition in Roux-En-Y Gastric Bypass Surgery. *Int J Comput Assist Radiol Surg* 2024, doi:10.1007/S11548-024-03166-3.

109. Komatsu, M.; Kitaguchi, D.; Yura, M.; Takeshita, N.; Yoshida, M.; Yamaguchi, M.; Kondo, H.; Kinoshita, T.; Ito, M. Automatic Surgical Phase Recognition-Based Skill Assessment in Laparoscopic Distal Gastrectomy Using Multicenter Videos. *Gastric Cancer* 2024, *27*, 187–196, doi:10.1007/S10120-023-01450-W.

110. Sasaki, K.; Ito, M.; Kobayashi, S.; Kitaguchi, D.; Matsuzaki, H.; Kudo, M.; Hasegawa, H.; Takeshita, N.; Sugimoto, M.; Mitsunaga, S.; et al. Automated Surgical Workflow Identification by Artificial Intelligence in Laparoscopic Hepatectomy: Experimental Research. *International Journal of Surgery* 2022, *105*, doi:10.1016/J.IJSU.2022.106856.

111. Madani, A.; Namazi, B.; Altieri, M.S.; Hashimoto, D.A.; Rivera, A.M.; Pucher, P.H.; Navarrete-Welton, A.; Sankaranarayanan, G.; Brunt, L.M.; Okrainec, A.; et al. Artificial Intelligence for Intraoperative Guidance: Using Semantic Segmentation to Identify Surgical Anatomy during Laparoscopic Cholecystectomy. *Ann Surg* 2022, *276*, 363–369, doi:10.1097/SLA.0000000000004594.

112. Cheng, K.; You, J.; Wu, S.; Chen, Z.; Zhou, Z.; Guan, J.; Peng, B.; Wang, X. Artificial Intelligence-Based Automated Laparoscopic Cholecystectomy Surgical Phase Recognition and Analysis. *Surg Endosc* 2022, *36*, 3160–3168, doi:10.1007/S00464-021-08619-3.

113. Golany, T.; Aides, A.; Freedman, D.; Rabani, N.; Liu, Y.; Rivlin, E.; Corrado, G.S.; Matias, Y.; Khoury, W.; Kashtan, H.; et al. Artificial Intelligence for Phase Recognition in Complex Laparoscopic Cholecystectomy. *Surg Endosc* 2022, *36*, 9215–9223, doi:10.1007/S00464-022-09405-5.

114. Shinozuka, K.; Turuda, S.; Fujinaga, A.; Nakanuma, H.; Kawamura, M.; Matsunobu, Y.; Tanaka, Y.; Kamiyama, T.; Ebe, K.; Endo, Y.; et al. Artificial Intelligence Software Available for Medical Devices: Surgical Phase Recognition in Laparoscopic Cholecystectomy. *Surg Endosc* 2022, *36*, 7444–7452, doi:10.1007/S00464-022-09160-7.

115. Laplante, S.; Namazi, B.; Kiani, P.; Hashimoto, D.A.; Alseidi, A.; Pasten, M.; Brunt, L.M.; Gill, S.; Davis, B.; Bloom, M.; et al. Validation of an Artificial Intelligence Platform for the Guidance of Safe Laparoscopic Cholecystectomy. *Surg Endosc* 2023, *37*, 2260–2268, doi:10.1007/S00464-022-09439-9.

116. Lopez-Lopez, V.; Morise, Z.; Albaladejo-González, M.; Gavara, C.G.; Goh, B.K.P.; Koh, Y.X.; Paul, S.J.; Hilal, M.A.; Mishima, K.; Krürger, J.A.P.; et al. Explainable Artificial Intelligence Prediction-Based Model in Laparoscopic Liver Surgery for Segments 7 and 8: An International Multicenter Study. *Surg Endosc* 2024, *38*, doi:10.1007/S00464-024-10681-6,.

117. Masum, S.; Hopgood, A.; Stefan, S.; Flashman, K.; Khan, J. Data Analytics and Artificial Intelligence in Predicting Length of Stay, Readmission, and Mortality: A Population-Based Study of Surgical Management of Colorectal Cancer. *Discover Oncology* 2022, *13*, 11, doi:10.1007/s12672-022-00472-7.

118. Lopez-Lopez, V.; Maupoey, J.; López-Andujar, R.; Ramos, E.; Mils, K.; Martinez, P.A.; Valdivieso, A.; Garcés-Albir, M.; Sabater, L.; Valladares, L.D.; et al. Machine Learning-Based Analysis in the

Management of Iatrogenic Bile Duct Injury During Cholecystectomy: A Nationwide Multicenter Study. *Journal of Gastrointestinal Surgery* 2022, *26*, 1713–1723, doi:10.1007/s11605-022-05398-7.

119. Cai, Z.H.; Zhang, Q.; Fu, Z.W.; Fingerhut, A.; Tan, J.W.; Zang, L.; Dong, F.; Li, S.C.; Wang, S.L.; Ma, J.J. Magnetic Resonance Imaging-Based Deep Learning Model to Predict Multiple Firings in Double-Stapled Colorectal Anastomosis. *World J Gastroenterol* 2023, *29*, 536–548, doi:10.3748/WJG.V29.I3.536.

120. Dayan, D.; Dvir, N.; Agbariya, H.; Nizri, E. Implementation of Artificial Intelligence-Based Computer Vision Model in Laparoscopic Appendectomy: Validation, Reliability, and Clinical Correlation. *Surg Endosc* 2024, *38*, 3310–3319, doi:10.1007/s00464-024-10847-2.

121. Arpaia, P.; Bracale, U.; Corcione, F.; De Benedetto, E.; Di Bernardo, A.; Di Capua, V.; Duraccio, L.; Peltrini, R.; Prevete, R. Assessment of Blood Perfusion Quality in Laparoscopic Colorectal Surgery by Means of Machine Learning. *Sci Rep* 2022, *12*, 14682, doi:10.1038/s41598-022-16030-8.

122. Gillani, M.; Rupji, M.; Paul Olson, T.J.; Sullivan, P.; Shaffer, V.O.; Balch, G.C.; Shields, M.C.; Liu, Y.; Rosen, S.A. Objective Performance Indicators Differ in Obese and Nonobese Patients during Robotic Proctectomy. *Surgery (United States)* 2024, doi:10.1016/J.SURG.2024.08.015.

123. Emile, S.H.; Horesh, N.; Garoufalia, Z.; Gefen, R.; Rogers, P.; Wexner, S.D. An Artificial Intelligence-Designed Predictive Calculator of Conversion from Minimally Invasive to Open Colectomy in Colon Cancer. *Updates Surg* 2024, *76*, 1321–1330, doi:10.1007/S13304-024-01915-2.

124. Wang, H.N.; An, J.H.; Zong, L. Advances in Artificial Intelligence for Predicting Complication Risks Post-Laparoscopic Radical Gastrectomy for Gastric Cancer: A Significant Leap Forward. *World J Gastroenterol* 2024, *30*, 4669–4671, doi:10.3748/WJG.V30.I43.4669.

125. Velmahos, C.S.; Paschalidis, A.; Paranjape, C.N. The Not-So-Distant Future or Just Hype? Utilizing Machine Learning to Predict 30-Day Post-Operative Complications in Laparoscopic Colectomy Patients. *Am Surg* 2023, *89*, 5648–5654, doi:10.1177/00031348231167397.

126. Jo, S.J.; Rhu, J.; Kim, J.; Choi, G. seong; Joh, J.W. Indication Model for Laparoscopic Repeat Liver Resection in the Era of Artificial Intelligence: Machine Learning Prediction of Surgical Indication. *HPB* 2025, doi:10.1016/J.HPB.2025.02.016.

127. Li, Y.; Su, Y.; Shao, S.; Wang, T.; Liu, X.; Qin, J. Machine Learning–Based Prediction of Duodenal Stump Leakage Following Laparoscopic Gastrectomy for Gastric Cancer. *Surgery (United States)* 2025, *180*, doi:10.1016/J.SURG.2024.108999.

128. Cai, Z.H.; Zhang, Q.; Fu, Z.W.; Fingerhut, A.; Tan, J.W.; Zang, L.; Dong, F.; Li, S.C.; Wang, S.L.; Ma, J.J. Magnetic Resonance Imaging-Based Deep Learning Model to Predict Multiple Firings in Double-Stapled Colorectal Anastomosis. *World J Gastroenterol* 2023, *29*, 536–548, doi:10.3748/WJG.V29.I3.536,.

129. Lippenberger, F.; Ziegelmayer, S.; Berlet, M.; Feussner, H.; Makowski, M.; Neumann, P.-A.; Graf, M.; Kaissis, G.; Wilhelm, D.; Braren, R.; et al. Development of an Image-Based Random

Forest Classifier for Prediction of Surgery Duration of Laparoscopic Sigmoid Resections. *Int J Colorectal Dis* 2024, *39*, 21, doi:10.1007/s00384-024-04593-z.

130. Zhou, C.M.; Li, H.J.; Xue, Q.; Yang, J.J.; Zhu, Y. Artificial Intelligence Algorithms for Predicting Post-Operative Ileus after Laparoscopic Surgery. *Heliyon* 2024, *10*, doi:10.1016/J.HELIYON.2024.E26580.

131. Aoyama, Y.; Matsunobu, Y.; Etoh, T.; Suzuki, K.; Fujita, S.; Aiba, T.; Fujishima, H.; Empuku, S.; Kono, Y.; Endo, Y.; et al. Artificial Intelligence for Surgical Safety during Laparoscopic Gastrectomy for Gastric Cancer: Indication of Anatomical Landmarks Related to Postoperative Pancreatic Fistula Using Deep Learning. *Surg Endosc* 2024, doi:10.1007/S00464-024-11117-X.

132. Du, C.; Li, J.; Zhang, B.; Feng, W.; Zhang, T.; Li, D. Intraoperative Navigation System with a Multi-Modality Fusion of 3D Virtual Model and Laparoscopic Real-Time Images in Laparoscopic Pancreatic Surgery: A Preclinical Study. *BMC Surg* 2022, *22*, doi:10.1186/S12893-022-01585-0.

133. Kasai, M.; Uchiyama, H.; Aihara, T.; Ikuta, S.; Yamanaka, N. Laparoscopic Projection Mapping of the Liver Portal Segment, Based on Augmented Reality Combined With Artificial Intelligence, for Laparoscopic Anatomical Liver Resection. *Cureus* 2023, doi:10.7759/CUREUS.48450.

134. Ryu, S.; Imaizumi, Y.; Goto, K.; Iwauchi, S.; Kobayashi, T.; Ito, R.; Nakabayashi, Y. Feasibility of Simultaneous Artificial Intelligence-Assisted and NIR Fluorescence Navigation for Anatomical Recognition in Laparoscopic Colorectal Surgery. *J Fluoresc* 2024, doi:10.1007/S10895-024-04030-Y,.

135. Garcia-Granero, A.; Jerí Mc-Farlane, S.; Gamundí Cuesta, M.; González-Argente, F.X. Application of 3D-Reconstruction and Artificial Intelligence for Complete Mesocolic Excision and D3 Lymphadenectomy in Colon Cancer. *Cir Esp* 2023, *101*, 359–368, doi:10.1016/J.CIRESP.2022.10.023.

136. Guan, P.; Luo, H.; Guo, J.; Zhang, Y.; Jia, F. Intraoperative Laparoscopic Liver Surface Registration with Preoperative CT Using Mixing Features and Overlapping Region Masks. *Int J Comput Assist Radiol Surg* 2023, *18*, 1521–1531, doi:10.1007/S11548-023-02846-W.

137. Ali, S.; Espinel, Y.; Jin, Y.; Liu, P.; Güttner, B.; Zhang, X.; Zhang, L.; Dowrick, T.; Clarkson, M.J.; Xiao, S.; et al. An Objective Comparison of Methods for Augmented Reality in Laparoscopic Liver Resection by Preoperative-to-Intraoperative Image Fusion from the MICCAI2022 Challenge. *Med Image Anal* 2025, *99*, 103371, doi:10.1016/J.MEDIA.2024.103371.

138. Robu, M.R.; Edwards, P.; Ramalhinho, J.; Thompson, S.; Davidson, B.; Hawkes, D.; Stoyanov, D.; Clarkson, M.J. Intelligent Viewpoint Selection for Efficient CT to Video Registration in Laparoscopic Liver Surgery. *Int J Comput Assist Radiol Surg* 2017, *12*, 1079–1088, doi:10.1007/S11548-017-1584-7.

139. Wei, R.; Li, B.; Mo, H.; Lu, B.; Long, Y.; Yang, B.; Dou, Q.; Liu, Y.; Sun, D. Stereo Dense Scene Reconstruction and Accurate Localization for Learning-Based Navigation of Laparoscope in Minimally Invasive Surgery. *IEEE Trans Biomed Eng* 2023, *70*, 488–500, doi:10.1109/TBME.2022.3195027.

140. Nicolaou, M.; James, A.; Lo, B.P.L.; Darzi, A.; Yang, G.-Z. Invisible Shadow for Navigation and Planning in Minimal Invasive Surgery. In; 2005; pp. 25–32.

141. Calinon, S.; Bruno, D.; Malekzadeh, M.S.; Nanayakkara, T.; Caldwell, D.G. Human-Robot Skills Transfer Interfaces for a Flexible Surgical Robot. *Comput Methods Programs Biomed* 2014, *116*, 81–96, doi:10.1016/J.CMPB.2013.12.015.

142. Zheng, Q.; Yang, R.; Ni, X.; Yang, S.; Jiang, Z.; Wang, L.; Chen, Z.; Liu, X. Development and Validation of a Deep Learning-Based Laparoscopic System for Improving Video Quality. *Int J Comput Assist Radiol Surg* 2022, *18*, 257–268, doi:10.1007/s11548-022-02777-y.

143. Cheng, Q.; Dong, Y. Da Vinci Robot-Assisted Video Image Processing under Artificial Intelligence Vision Processing Technology. *Comput Math Methods Med* 2022, *2022*, 1–10, doi:10.1155/2022/2752444.

144. Akbari, H.; Kosugi, Y.; Khorgami, Z. Image-Guided Preparation of the Calot's Triangle in Laparoscopic Cholecystectomy. *Proceedings of the 31st Annual International Conference of the IEEE Engineering in Medicine and Biology Society: Engineering the Future of Biomedicine, EMBC 2009* 2009, 5649–5652, doi:10.1109/IEMBS.2009.5333766.

145. Katić, D.; Wekerle, A.-L.; Görtler, J.; Spengler, P.; Bodenstedt, S.; Röhl, S.; Suwelack, S.; Kenngott, H.G.; Wagner, M.; Müller-Stich, B.P.; et al. Context-Aware Augmented Reality in Laparoscopic Surgery. *Computerized Medical Imaging and Graphics* 2013, *37*, 174–182, doi:10.1016/j.compmedimag.2013.03.003.

146. Beyersdorffer, P.; Kunert, W.; Jansen, K.; Miller, J.; Wilhelm, P.; Burgert, O.; Kirschniak, A.; Rolinger, J. Detection of Adverse Events Leading to Inadvertent Injury during Laparoscopic Cholecystectomy Using Convolutional Neural Networks. *Biomedizinische Technik* 2021, *66*, 413–421, doi:10.1515/BMT-2020-0106.

147. Salazar-Colores, S.; Moreno, H.A.; Moya, U.; Ortiz-Echeverri, C.J.; Tavares de la Paz, L.A.; Flores, G. Removal of Smoke Effects in Laparoscopic Surgery via Adversarial Neural Network and the Dark Channel Prior. *Cirugia y Cirujanos (English Edition)* 2022, *90*, 74–83, doi:10.24875/CIRU.20000951.

148. Wagner, M.; Bihlmaier, A.; Kenngott, H.G.; Mietkowski, P.; Scheikl, P.M.; Bodenstedt, S.; Schiepe-Tiska, A.; Vetter, J.; Nickel, F.; Speidel, S.; et al. A Learning Robot for Cognitive Camera Control in Minimally Invasive Surgery. *Surg Endosc* 2021, *35*, 5365–5374, doi:10.1007/S00464-021-08509-8.

149. He, W.; Zhu, H.; Rao, X.; Yang, Q.; Luo, H.; Wu, X.; Gao, Y. Biophysical Modeling and Artificial Intelligence for Quantitative Assessment of Anastomotic Blood Supply in Laparoscopic Low Anterior Rectal Resection. *Surgical Endoscopy* 2025, doi:10.1007/S00464-025-11693-6.

150. Acosta-Mérida, M.A.; Sánchez-Guillén, L.; Álvarez Gallego, M.; Barber, X.; Bellido Luque, J.A.; Sánchez Ramos, A. Encuesta Nacional Sobre La Gobernanza de Datos y Cirugía Digital: Desafíos y Oportunidades de Los Cirujanos En La Era de La Inteligencia Artificial. *Cir Esp* 2025, *103*, 143–152, doi:10.1016/j.ciresp.2024.12.003.

151. Lünse, S.; Wisotzky, E.L.; Beckmann, S.; Paasch, C.; Hunger, R.; Mantke, R. Technological Advancements in Surgical Laparoscopy Considering Artificial Intelligence: A Survey among Surgeons in Germany. *Langenbecks Arch Surg* 2023, *408*, 405, doi:10.1007/s00423-023-03134-6.

152. Shafiei, S.B.; Shadpour, S.; Mohler, J.L. An Integrated Electroencephalography and Eye-Tracking Analysis Using EXtreme Gradient Boosting for Mental Workload Evaluation in Surgery. *Hum Factors* 2024, doi:10.1177/00187208241285513.
